# Characteristics and Evolution of sill-driven off-axis hydrothermalism in Guaymas Basin – the Ringvent site

**DOI:** 10.1038/s41598-019-50200-5

**Published:** 2019-09-25

**Authors:** Andreas Teske, Luke J. McKay, Ana Christina Ravelo, Ivano Aiello, Carlos Mortera, Fernando Núñez-Useche, Carles Canet, Jeffrey P. Chanton, Benjamin Brunner, Christian Hensen, Gustavo A. Ramírez, Ryan J. Sibert, Tiffany Turner, Dylan White, Christopher R. Chambers, Andrew Buckley, Samantha B. Joye, S. Adam Soule, Daniel Lizarralde

**Affiliations:** 1 University of North Carolina at Chapel Hill, Department of Marine Sciences, Chapel Hill, USA; 2 Montana State University, Center for Biofilm Engineering, Bozeman, USA; 3 University of California at Santa Cruz, Department of Ocean Sciences, Santa Cruz, USA; 4 San Jose State University, Moss Landing Marine Laboratory, Moss Landing, USA; 5 Universidad Nacional Autónoma de México, Instituto de Geofísica, Mexico City, Mexico; 6 Universidad Nacional Autónoma de México, Instituto de Geología, Mexico City, Mexico; 7 Universidad Nacional Autónoma de México, Centro de Ciencias de la Atmósfera, Mexico City, Mexico; 8 Florida State University, Department of Earth, Ocean and Atmospheric Sciences, Tallahassee, USA; 9 The University of Texas at El Paso, Department of Geological Sciences, El Paso, USA; 10 GEOMAR Helmholtz Centre for Ocean Research, Kiel, Germany; 11 University of Georgia, Department of Marine Sciences, Athens, USA; 12 Woods Hole Oceanographic Institution, Geology & Geophysics Department, Woods Hole, USA

**Keywords:** Carbon cycle, Marine chemistry, Environmental microbiology

## Abstract

The Guaymas Basin spreading center, at 2000 m depth in the Gulf of California, is overlain by a thick sedimentary cover. Across the basin, localized temperature anomalies, with active methane venting and seep fauna exist in response to magma emplacement into sediments. These sites evolve over thousands of years as magma freezes into doleritic sills and the system cools. Although several cool sites resembling cold seeps have been characterized, the hydrothermally active stage of an off-axis site was lacking good examples. Here, we present a multidisciplinary characterization of Ringvent, an ~1 km wide circular mound where hydrothermal activity persists ~28 km northwest of the spreading center. Ringvent provides a new type of intermediate-stage hydrothermal system where off-axis hydrothermal activity has attenuated since its formation, but remains evident in thermal anomalies, hydrothermal biota coexisting with seep fauna, and porewater biogeochemical signatures indicative of hydrothermal circulation. Due to their broad potential distribution, small size and limited life span, such sites are hard to find and characterize, but they provide critical missing links to understand the complex evolution of hydrothermal systems.

## Introduction

The Guaymas Basin in the Gulf of California is a young marginal seafloor spreading system where new igneous crust forms in an environment of rapid sedimentation^1,2^. As a result, shallow magmatic emplacement in Guaymas Basin occurs as doleritic sill intrusions into organic-rich, predominately diatomaceous sediments, as opposed to the extrusive basaltic flows commonly observed at mid-ocean ridges^3^. The intruded magmas drive hydrothermal flow, and the resulting thermal and chemical gradients create dynamic bio-geochemical environments. These environments vary both temporally, as sill-driven systems have a life cycle related to cooling of the host sill, and spatially, where on- and off-axis systems represent key end-member environments^4,5^.

The axes of the two (northern and southern) overlapping spreading segments within Guaymas Basin are defined by ~200-m-deep axial troughs (Supplementary Fig. 1). Until recently, nearly all Guaymas Basin hydrothermal observations have been obtained from the southern trough, where hydrothermal sediments, mounds, chimneys, microbial mats and vent fauna form a complex hydrothermal seafloor landscape that appears to coincide with buried sills and elevated heat flow^5,6^. On-axis systems might be driven by heat from both shallow sills and deeper crustal magma that maintains a persistently high heat flow over timescales much longer than the life cycle of individual sills^7^. The extensional faulting that maintains the axial troughs is likely to be also responsible for permeable pathways that promote robust, deep fluid circulation. For example, a large hydrothermal mound characterized by strong, channelized flow of extremely hot fluids through axial-parallel faults was recently discovered on the southwestern edge of the northern trough^8^.

Off-axis sites with gas seepage, fluid flow, elevated temperatures and seafloor seep communities have been found up to 20 to 30 km distant from the spreading center^9^. Igneous sills are observed beneath those sites where coincident seismic data are available, and sedimentary structures indicate that those sills are significantly younger than the plate age, implying that they were intruded off-axis^9^. The physical, chemical and biological evolution of off-axis systems is likely to differ significantly from those on-axis. Since off-axis sills are intruded into thicker sediments, tectonic permeability does not affect their cooling, and a deep-crustal, plate-boundary source of heat is absent. The Guaymas Basin off-axis systems may be quite similar, however, to sills intruded into sedimentary basins during the early stages of oceanic large igneous province emplacement^10,11^. As such, the Guaymas Basin off-axis sites can be considered a model system for investigating sill-driven sediment alteration, mineral precipitation/dissolution, and carbon mobilization that reintroduces buried carbon and other elements to the hydrosphere and potentially atmosphere, processes believed to link large igneous events to major shifts in global climate and mass-extinction events throughout Earth history^10–12^.

Here we describe results from detailed mapping, submersible sampling and coring of Ringvent, a large off-axis circular hydrothermal formation overlying a buried saucer-shaped sill. Our results indicate that this site is evolving from active, sill-driven hydrothermalism – characterized by the emergence of hot, reducing fluids - towards a cooler phase of localized fluid circulation dominated by methane flow. The identification of hydrothermalism at Ringvent required a sequence of observations at increasingly smaller spatial scales, beginning with deep-towed seafloor mapping that identified numerous sites of likely seafloor fluid flow and followed by site-specific coring, ROV mapping, and submersible sampling at Ringvent. Such hydrothermally active sites may be intrinsically hard to identify, given the expected short duration (<10 K years)^13^ of active sill-driven hydrothermal flow and the likelihood that these sites evolve into cold seeps through time. Previous surveys concluded that carbon mobilization at off-axis sites in this region occurs predominantly as cold methane seepage and that off-axis hydrothermal flow has to be estimated with care considering the lifespan of sill induced systems^4^. Evidently, resolving and mapping the extent of hydrothermalism versus cold seepage in the greater Guaymas Basin system requires site-specific studies.

## Results

### Mapping Ringvent

**Figure 1:**
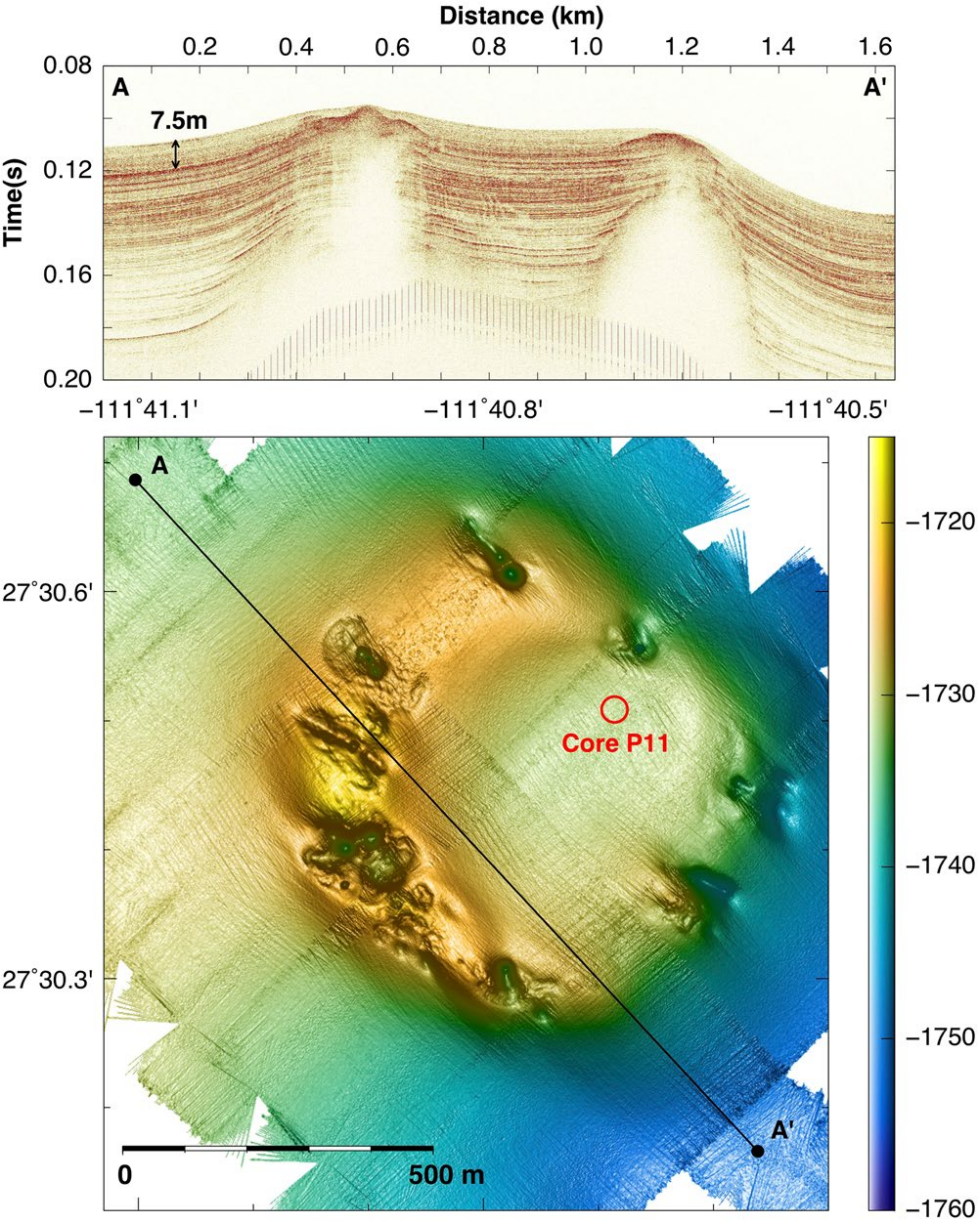
Seismic and bathymetric survey of Ringvent. (**A**) The shallow subsurface seismic profile obtained by AUV *Sentry* shows that continuously visible sediment stratification is obliterated in dome-shaped whiteout zones that represent gas intrusions; flares of gas and fluid flow emerge from the tops of these gas intrusions. The profile is crossing Ringvent from the Northwest [A, -186.140640/27.5108089] to the Southeast [A’; -186.126694/27.5027676]. (**B**) Contour-shaded *Sentry* bathymetry of Ringvent, with seismic profile line inserted. A donut-shaped topographical high characterized by abundant tubeworm colonies and carbonate outcrops, and incised by gullies and pockmark-like depressions, rises ca. 20 meter above the seafloor, and surrounds a central sedimented bowl. The seafloor is gradually rising westwards.

Ringvent is located 28.5 km northwest of the northern Guaymas Basin spreading center, in the central Gulf of California (Supplementary Fig. 1). A seismic transect across the site shows a sedimented basin surrounded by a ring-shaped mound (Fig. 1). The bathymetric map shows that the ring-shaped mound rises to ca. 15 to 20 meters above the surrounding seafloor, with greater topographical highs in the western portion. Subbottom seismic imaging of the Ringvent area shows shallow reflective sedimentary layers on the outside of the ring structure and within the sedimented basin; specific reflectors intensify and approach the sediment surface near the topographical high of the ring structure. Underneath the ring, seismic energy is absorbed by free gas present in seismically blanked dome-shaped intrusions; the tops of these gas intrusions underlie the crests of the ring. The extent of shallow gas and the up-dip brightening of reflectivity towards the blanked zones deeper in the subsurface suggest ongoing gas and fluid transport (Fig. 1).

**Figure 2:**
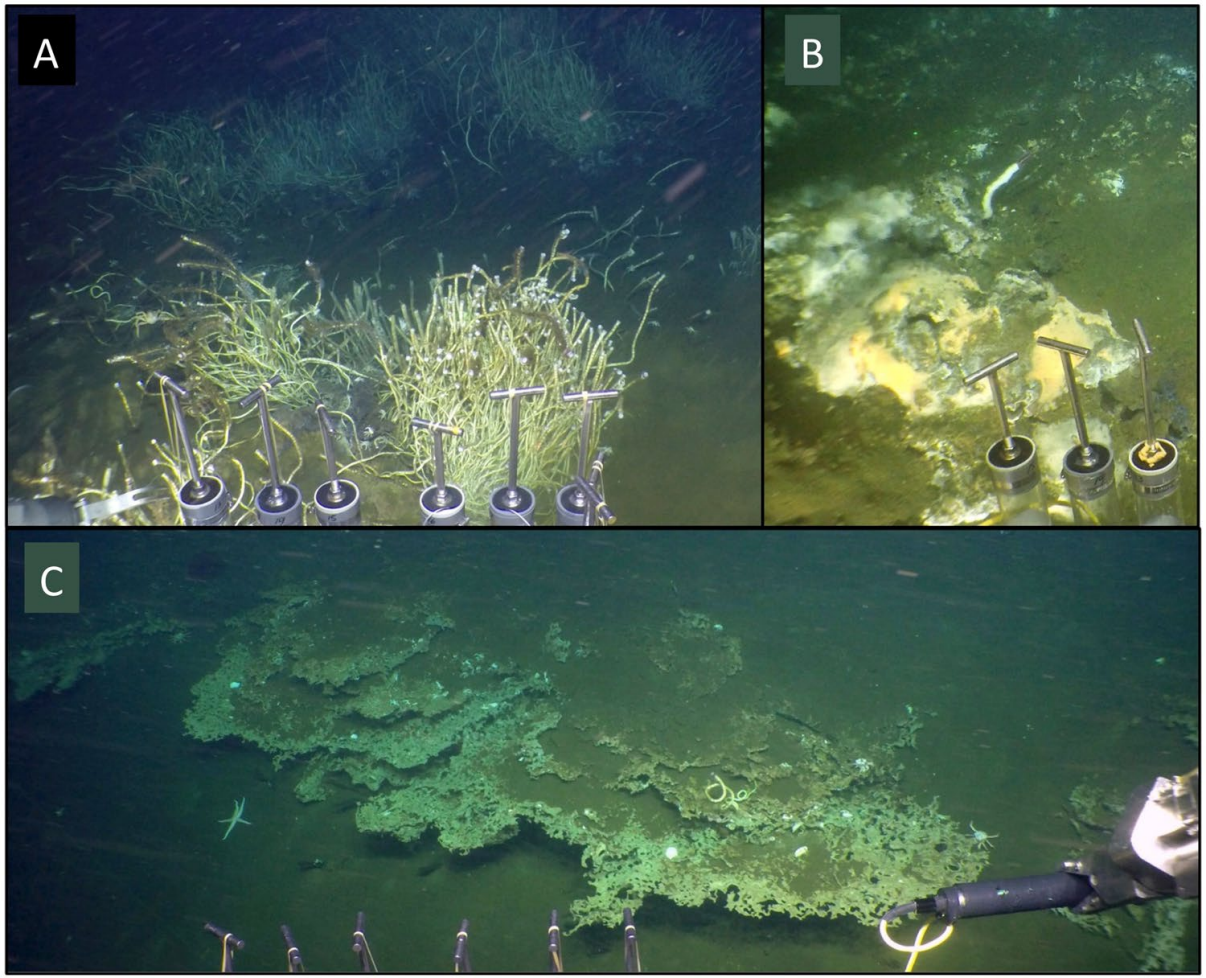
*In-situ* observations of seafloor minerals and fauna. (**A**) Tubeworm colonies resembling *Lamellibrachia* occurring on the Ringvent mound (ORP sampling site, *Alvin* dive 4864). (**B**) A single sulfur-oxidizing *Riftia* tubeworm growing on a small mat-covered mound that provides warm fluids of ca. 10–20 °C (Table 2), visible as shimmering optical distortions in the warm water plume (near ORP site, *Alvin* dive 4864). (**C**) Lace-like authigenic minerals emerging from sediment cover at incised gully (near heatflow site 5, *Alvin* dive 4865).

Bathymetric mapping by AUV *Sentry* shows large 5- to 10-m deep gullies incised laterally into the ring crest, and similar sedimented hollows on top of the crest (Supplementary Fig. 2). These locations are characterized by elevated heat flow, water column redox and thermal anomalies (Supplementary Fig. 3), exposed authigenic mineral concretions, widespread *Lamellibrachia* tubeworm colonies, localized venting of warm fluids, and mats of chemosynthetic sulfur-oxidizing bacteria and individual sulfur-dependent *Riftia* tubeworms that resemble their counterparts in the Guaymas Basin spreading center (Fig. 2). Photomosaic surveys by *Sentry* of the western portion of the ring-shaped mound reveal small outcrops and rocks overgrown with yellow and white mats of sulfur-oxidizing bacteria^14^, and cracks in the hard substrate that are populated with galatheid crabs, and tubeworms resembling *Lamellibrachia* (Supplementary Fig. 4). Thin sediments of a few centimeters on the ring structure accumulate locally in sedimented hollows and incised gullies, and allow coring and heatflow measurements (Mound 1 and 2 site, ORP site; Supplementary Fig. 2). Thermal gradients in these surficial sediments measured with *Alvin’s* heat flow probe reach approximately 5 °C/m, exceeding thermal gradients in seafloor sediments at the outer perimeter of the ring (0.6–0.7 °C/m) by an order of magnitude (Table 1, and Supplementary Fig. 5). The moderately warm surficial sediments in sedimented spots on the Ringvent mound contain porewater sulfide at concentrations of several hundred micromolar (Supplemental Data 1). Localized hydrothermal fluid flow with significantly elevated temperatures (ca. 20 °C to 70 °C, Tables 1 and 2) coincides with white and yellow-orange mats of large, sulfur-oxidizing filamentous bacteria of the family *Beggiatoaceae* – a microbial indicator of sulfide-rich, reducing hydrothermal flow^14^ – and individual *Riftia* tubeworms surrounded by shimmering warm water of ca. 20 °C (Fig. 2). *Riftia* require turbulent mixing of sulfidic vent fluids with oxic seawater to sustain their sulfur-oxidizing, CO_2_-fixing bacterial endobionts^15^; in Guaymas Basin they may also be able to use thiosulfate emerging from the sediments as a sulfur source^16^.

**Table 1:** Thermal gradients [°C] in Ringvent sediments, measured by *Alvin* heatflow probe during *Alvin* dives 4864 and 4865, above and below the sediment/water interface.

	ORP1	ORP 2	ORP3	Mound1	*Riftia*	HF1	HF2	HF3	HF4	HF5
20 cm	---	2.90	----	---	---	---	---	---	---	---
10 cm	---	---	---	2.90	---	---	---	---	---	---
5 cm	---	---	---	---	3.15	---	---	---	---	---
0 cm	---	2.90	---	---	---	---	---	---	---	27.71
10 cm	3.21		2.90	3.39	---	2.94	2.94	2.95	2.99	---
15 cm	---	---	---	---	9.42					
20 cm	---	4.85	---	---	---	---	---	---	---	74.53
30 cm	4.45	---	3.64	4.64	---	3.08	3.07	3.09	3.40	---
35 cm	---	---	---	---	12.94	---	---	---	---	---
40 cm	---	5.38	---	---	---	---	---	---	---	75.21
50 cm	5.41	---	4.65	5.88	---	3.29	3.28	3.32	3.99	---
55 cm	---	---	---	---	11.15	---	---	---	---	---
60 cm	---	---	---	---	---	---	---	---	---	70.76
70 cm	6.00	----	5.56	7.14	---	3.4	3.42	3.47	4.50	---
75 cm	---	---	---	---	12.66	---	---	---	---	---
80 cm	---	---	---	---	---	---	---	---	---	72.44
90 cm	6.70	---	6.50	---	---	3.51	3.54	3.58	5.00	---

The thermal data in the *Riftia* column average five fluctuating temperature readings at a porous mineral mound colonized by a *Riftia*. Locations and *in*-*situ* context are shown in Supplementary Figs 2 and 5.

**Table 2:** Five fluctuating temperature readings [°C] were obtained over 10 minutes (timepoints given in Greenwich Mean Time, GMT) during *Alvin* dive 4864 at a porous mineral mound colonized by a *Riftia*, near the ORP site (Supplementary Fig. 5).

	Reading 1	Reading 2	Reading 3	Reading 4	Reading 5
GMT of thermal reading	21:29	21:31	21:32	21:33	21:38
5 cm in overlying water	3.10	2.92	2.95	3.41	3.37
15 cm below surface	3.63	20.17	14.6	4.89	3.83
35 cm below surface	22.52	10.13	9.41	11.02	11.63
55 cm below surface	15.02	21.41	6.83	6.27	6.21
75 cm below surface	11.23	10.44	12.05	13.86	15.73

The temperature probe could not be fully inserted into the mineral mound, and the top sensor remained ca. 5 cm above the ground.

### Silica mineral concretions

**Figure 3:**
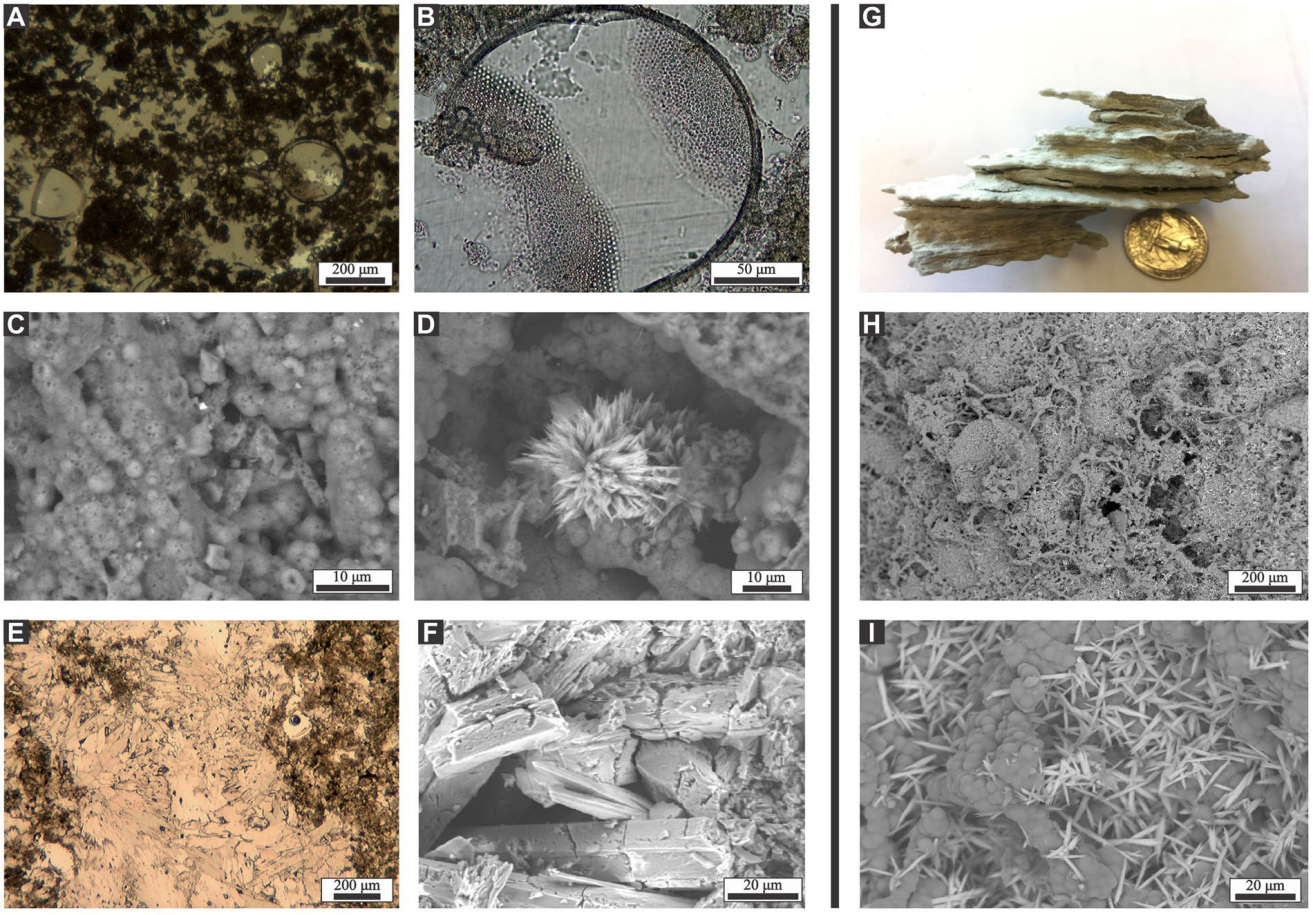
Minerals from the surface and subsurface of Ringvent. **(A**–**F**) Selected images of Ringvent minerals, collected at the seafloor by *Alvin*. (**A**) diatoms embedded in silica matrix; (**B**) diatom frustule showing partial dissolution; (**C**) opal-A microspheres forming the matrix; (**D**) barite rosette within the matrix formed by opal-A microspheres; (**E,F**) fibrous aragonite cement crystals. Images A to D come from a large silicate sample collected at the ORP site; specimens shown in E and F were collected from a biologically active small mound at the Mound 1 location (Supplemental Fig. 6). A and E are photomicrographs of thin sections; B-D and F are SEM microphotographs; analyses were performed by C. Canet and F. Núñez-Useche, UNAM. G-I) Selected images of subsurface silicate. (**G**). Silicate nodule collected at 2.35 m sediment depth in core P11. (**H**), SEM/EDX microphotographs of the nodule surface showing bead-like formations coated with silica at intermediate and (**I**) at high magnification, with barite crystal rosettes on the silica matrix. Analyses were performed by I. Aiello, MLML.

Exposed seafloor mineral deposits were sampled at the ORP and Mound 1 locations for petrographic and compositional analysis (Supplementary Fig. 6). Scanning electron microscopy/energy dispersive X-ray spectroscopy (SEM/EDS) and X-ray diffraction analysis show that these deposits consist predominantly of a silica matrix formed by opal-A microspheres embedding diatom frustules, with subordinate amounts of low magnesium calcite, fibrous aggregates of aragonite, and platy single crystals and rosette aggregates of barite (Fig. 3). These minerals strongly differ from the classical cold seep carbonate deposits, formed through the precipitation of carbonates by microbially-mediated methane oxidation. The dominance of silica in the Ringvent minerals was confirmed by SEM/EDS analyses of Ringvent samples at the Woods Hole Oceanographic Institution (Supplementary Fig. 7).

Silica precipitates were also collected by piston coring from the subsurface sediments in the central lower-lying Ringvent area (Supplementary Fig. 8). A silica nodule from 2.35 m depth, recovered at this location by piston coring, has a bead-like texture of non-crystalline opal-A microspheres (Fig. 3G) that resembles laminated silica-carbonate hydrothermal deposits from intertidal hot springs with temperatures of 54 to 87 °C, at the eastern coast of the Baja California peninsula^17^. The petrographic identification of the opal-A silica was confirmed by XRD analysis (Supplementary Data 2). Very similar laminated structures – microbial filaments coated with amorphous silica – occurring in Guaymas Basin siliceous deposits are interpreted as the result of conductive cooling, without silica dilution by inmixing of seawater^18^. These similarities suggest that the silica-rich deposits of Ringvent form by diffuse flow and conductive cooling of silica-rich hydrothermal solutions.

### Carbonate composition and isotopic signatures

**Figure 4:**
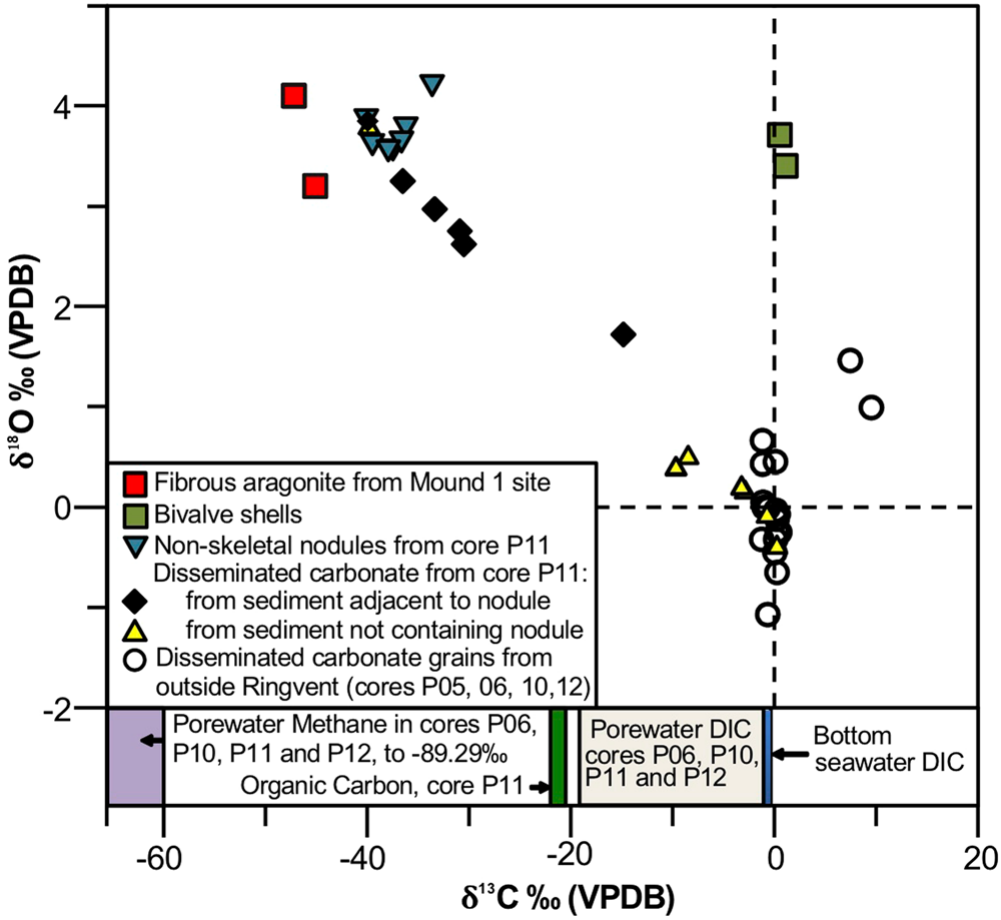
Carbonate oxygen and carbon isotopes. Plot of δ^13^C vs. δ^18^O values in carbonate nodules, sedimentary carbonates, and other samples from *El Puma* sediment cores P5/6, P10, P11 and P12. The stable isotope measurements for sedimentary carbonate were made on bulk sediment after removing organic carbon by roasting, and therefore include all carbonate from both skeletal and authigenic sources. The data are tabulated in Supplementary Data 3.

**Figure 5:**
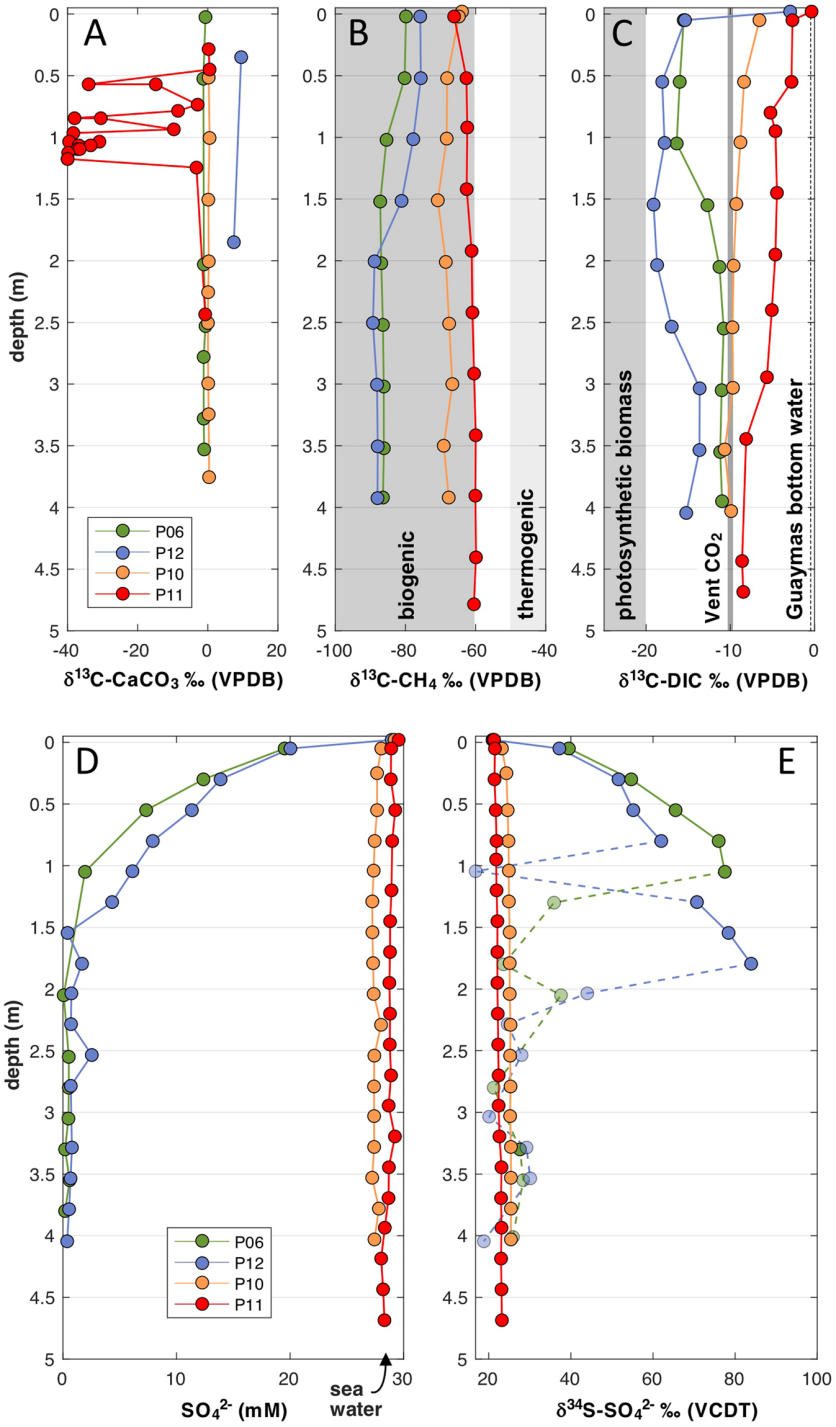
Geochemical profiles of piston-cored sediments. The plots are contrasting the geochemical profiles of Ringvent core P11 against those from nearby control core P10 and Sonora Margin cores P5/6 and P12. (**A**) δ^13^C-isotopic composition for carbonate nodules and sedimentary carbonate. Porewater δ^13^C-isotopic profiles for (**B**) methane and (**C**) DIC. The range of biogenic methane is based on Guaymas Basin sediment data^31,32^; the range for thermogenic methane is based on δ^13^C-isotopic values near −42‰ for surficial hydrothermal sediments^19^, −43.2 to −50.8‰ in hydrothermal fluid^33^, and −40 to −44‰ in hydrothermal DSDP core 477^30^. (**D**) Porewater sulfate concentrations determined by ion chromatography. (**E**) δ^34^S profiles for porewater sulfate. Complete isotopic data for solid-phase carbonates are tabulated in Supplementary Data 3. Porewater DIC and methane data are tabulated in Supplementary Data 5A,B, and sulfate and sulfide data (concentrations only for sulfide) are tabulated in Supplementary Data 5C,D.

Since hydrothermal fluids in Guaymas Basin are rich in methane and dissolved inorganic carbon (DIC)^19^, and methane-derived carbonates are widespread in the northern Gulf of California^20,21^, we checked piston-cored subsurface sediments for indicators of methane cycling and a potential methane contribution to carbonate formation. In core P11 from the central Ringvent area, conspicuous non-skeletal (i.e., not derived from plankton shells) carbonate concretions are found below ca. 50 cm depth, forming a massive layer at ca. 1 to 1.20 m below the seafloor (Supplementary Fig. 8). This layer is unique to core P11; it does not appear in core P10, collected ca. 1 mile further west outside Ringvent, and also not in any other core collected during the 2014 Expedition (details in Methods: Field surveys). XRD examination (Supplemental Data 2) shows that these carbonates consist of magnesian calcite, compositionally similar to cold seep carbonates reported previously for Guaymas Basin^22^. These localized carbonate concretions exhibit light δ^13^C values from −37 to −41‰, whereas smaller, visually inconspicuous carbonate grains and particles in sediment surrounding these nodules show slightly heavier δ^13^C values from −37 to −30‰ (Figs 4 and 5A, and Supplementary Data 3). Carbonate nodules and grains in core P11 are significantly lighter than organic carbon in the same core, between −20.5 and −22‰ (see Methods), and previously analyzed sedimentary organic carbon and chemosynthetic microbial biomass (−21.6 and −22.1‰, respectively) in Guaymas Basin^23^, indicative of contributions from methane-derived carbon sources. In their δ^13^C isotopic composition, the nodules resemble independently collected carbonates (−36.5‰) from surface mud of Ringvent^22^, and differ from cold seep carbonates in Guaymas Basin (−46.6 and −44.7‰^4^; −45.2 and −47.6‰^21^). In contrast, sedimentary carbonates in core P11 above and below the nodules have moderate ^13^C depletion ranging from −15 to 0‰ (Figs 4 and 5A), and approach δ^13^C-DIC values of seawater (−0.6‰) in Guaymas Basin^23^. A clam shell found at 55 cm depth in core P11 has a stable carbon isotopic signature near seawater background (Fig. 4), and in this regard resembles other clam shell specimens found at Guaymas Basin off-axis seep sites^22^. Generally, δ^13^C values for sedimentary carbonate in cores outside Ringvent cluster near seawater background (Figs 4 and 5A).

The lightest δ^13^C values of this Ringvent dataset (−45.1 and −47.2‰; Fig. 4), were not found in the sedimentary carbonates and nodules of core P11, but in fibrous aragonite veins embedded in Ringvent silicate samples (Fig. 3) that were collected at a biologically active location at the Mound 1 site (Supplementary Fig. 6). These findings indicate that anaerobic methane oxidation occurs locally in the silica-dominated matrix of Ringvent, and imply the presence of methane and methane-oxidizing microorganisms.

### Carbonate origins

Potential formation conditions that inform the origins of the solid-phase carbonates in the Ringvent sediments were further constrained by oxygen isotope measurements. In the δ^13^C vs. δ^18^O plot of the carbonate nodules and sedimentary carbonates (Fig. 4), the most ^13^C-depleted carbonates (fibrous aragonite; nodules and grains; clam shells) showed a moderate enrichment in ^18^O (δ^18^O: 1.7 to 4.2‰), practically within the range of the authigenic carbonates (high-Mg calcite) from the Sonora Margin (2.0 to 6.4‰)^20^ and the Consag Basin (1.0 to 4.0‰)^21^. These values also fall in the range of the predicted δ^18^O values (up to 4.7‰) for aragonite precipitated in equilibrium with the current bottom water at *in-situ* temperatures of 2.9 °C (δ^18^O: 0.7‰)^20^, calculated using the experimental equation for this mineral^24^. Notably, δ^13^C and δ^18^O values co-vary strongly in the carbonates from core P11 (*R*: −0.8) indicating a mixing line between two end-members: methane-derived carbonates precipitated in isotopic equilibrium with relatively cold bottom waters, and biogenic carbonates in warmer surface seawater. Indeed, the petrographic analysis of the sediments confirms that biogenic carbonate (coccoliths, benthic and planktonic foraminifera) is the dominant carbonate phase in sediment outside the nodule-bearing interval. Carbonates collected in core P11 outside the nodule-bearing interval, and from cores outside Ringvent, displayed limited variation of δ^18^O, between −1.1 and 1.8‰ (Fig. 4; Supplementary Data 3), similar to typical values of biogenic carbonates in this region^25,26^.

To date the buried silica and carbonate deposits, we determined the local sedimentation rates based on the ^14^C-age of sedimentary organic matter, undistorted by methane or DIC-derived contributions (see Methods), for the piston coring locations of the 2014 cruise (R/V *El Puma*, Supplementary Data 4). The local rate of 0.286 mm/y for core P11 was the slowest sedimentation rate obtained for all *El Puma* cores, and slower than previously published rates of 0.88 to 1.2 mm/yr for the Baja California slopes of Guaymas Basin^27,28^. This low sedimentation rate may result from reduced terrestrial contributions at this central Guaymas Basin location, but input of fossil organic matter into sedimentary organic matter may also play a role. Independently measured sedimentation rates for several Guaymas Basin locations also showed the lowest rates (0.5 mm/yr) at Ringvent^4^.

### Porewater methane and DIC

Since the methane-rich hydrothermal and seep fluids of Guaymas Basin and the Sonora Margin carry the carbon isotopic imprints of different biological and hydrothermal sources, we examined porewater methane at Ringvent and other off-axis locations in Guaymas Basin. Porewater methane concentrations in core P11 range from near 1 to 1.5 mM, and gradually decreased to background levels within the upper 1.5 to 2 m (Supplementary Data 5).

Although decreasing methane concentrations suggest oxidation, the δ^13^C-CH_4_ profile in the core P11 does not indicate a specific, localized sediment horizon where methane oxidation generates heavier δ^13^C-CH_4_ signatures, as seen in the Sonora Margin cores at ca. 1.5 to 2 m depth (Fig. 5B), and possibly in an independently collected short pushcore from Ringvent (termed RingSeep)^4^, where a small methane pool (2 to 36 µM) changes from −57‰ at 20 cm depth towards −44.8‰ at the surface^4^. Instead, the δ^13^C-CH_4_ values remained consistently within a narrow range, from −60 to −66‰ (Fig. 5B), between microbially produced methane (−97.3 to −82‰) in fully reduced, cold subsurface sediments on the nearby Sonora Margin^29^, and in Sonora Margin cores P06 and P12 (−89.1 to −75.8‰), and methane in hot hydrothermal surface sediments (near −42‰)^19^ and hydrothermal subsurface sediments of DSDP hole 477 (−40 to −44‰)^30^ at the southern Guaymas spreading center. The closest matches to Ringvent are the intermediate δ^13^C-CH_4_ values near −60‰ in deep subsurface sediments of Guaymas Basin (DSDP hole 481) that are interpreted as mixtures of hydrothermal methane originating at deep sills and microbially produced methane in the upper sediment column^31,32^, and δ^13^C-CH_4_ values clustering near −55‰ in shallow sediments at several off-axis Guaymas Basin seep locations^4^. Near the top of core P11, residual methane is more strongly depleted in ^13^C, with values near −66‰, indicating the influence of methylotrophic methanogenesis that competes successfully with sulfate reduction in surficial marine sediments^29^. Methylotrophic methanogenesis is also compatible with the isotopic composition of trace methane (5 to 14 µM, Supplementary Data 5) observed throughout sediment core P10, collected outside Ringvent (Fig. 5B).

Although methane oxidation would generate ^13^C-depleted DIC, the δ^13^C-DIC values for core P11 stand out as the heaviest of all cores (Fig. 5C), and overlap with the δ^13^C-DIC range (−6 to +2.7‰) of seawater-impacted hydrothermal fluids^33^ and porewater from shallow hydrothermal sediments in Guaymas Basin^19^. Instead, these results suggest mixing of seawater DIC (−0.6‰) and hydrothermally derived DIC (−9.4‰)^22^. Seawater influence is also reflected by the low porewater DIC concentrations that increase from 2.5 mM, resembling Guaymas bottom water (2.34 mM)^23^, towards 4 mM downcore (Supplementary Data 5). Seawater inmixing and ventilation resolve the apparent contradiction that decreasing methane concentrations towards the surface are not accompanied by carbon-isotopic signatures of porewater methane oxidation.

### Sulfur cycling

Of all sediment cores in this study, core P11 has the highest porewater sulfate concentrations (28–29 mM), similar to seawater, throughout the entire depth range (Fig. 5D). Sulfide porewater concentrations are consistently below the detection limit (ca. 3 µM) within the upper 2.5 m and increase towards 80 µM only in the bottom third of the core. Higher sulfide porewater concentrations characterize surficial sediments on the Ringvent massif, from 105 µM at 22 cm depth^4^ to peaks of 950 µM within 18 cm (Supplementary Data 1). By comparison, core P10 outside Ringvent shows a fluctuating sulfide background of 20 to 100 µM, and the Sonora Margin cores P06 and P12 harbor very high biogenic sulfide concentrations reaching 5 to 10 mM (Supplementary Data 5). The isotopic composition of porewater sulfate was analyzed to obtain additional evidence for microbial sulfur cycling. Microbial sulfate reduction to sulfide discriminates against the heavy sulfur isotope ^34^S, enriching residual sulfate in ^34^S; the effect is reversed by oxidative sulfur cycling which returns isotopically light sulfur to the sulfate pool^34^. In contrast to the surficial sediments in Sonora Margin cores P06 and P12, which show massive biogenic ^34^S enrichment (δ^34^S-SO_4_^2−^ towards ≥80‰), the δ^34^S-SO_4_^2−^ profiles of cores P10 and P11 change only minimally and thus indicate limited sulfate-reducing activity (Fig. 5E, and Supplementary Data 5). In core P10, the surficial sediments show moderate ^34^S-enrichment and a very gradual increase towards δ^34^S of ~25.5‰ at depth, indicating that sulfate-reducing activity is limited to surficial sediments, likely fueled by planktonic organic matter. In contrast, core P11 displays an almost linear transition from isotopic compositions close to δ^34^S values of seawater sulfate (21‰) at the sediment surface towards δ^34^S values near 23‰ at the bottom of the core (Supplementary Data 5), suggesting mixing of surficial seawater and a subsurface sulfate pool. Together, the biogeochemical indicators of sulfur cycling in the central Ringvent sediments indicate a current regime of seawater inmixing, low sulfate-reducing activity, and lack of sulfide accumulation, which contrasts with the localized sulfidic conditions on the Ringvent mound that sustain sulfide-oxidizing microbial mats and symbiont-dependent invertebrates. The lack of sulfide likely inhibits anaerobic methane-oxidizing archaea, which generally require reducing conditions to be active^35^, and is consistent with methane removal predominantly by seawater inmixing and dilution.

### Microbial communities

**Figure 6:**
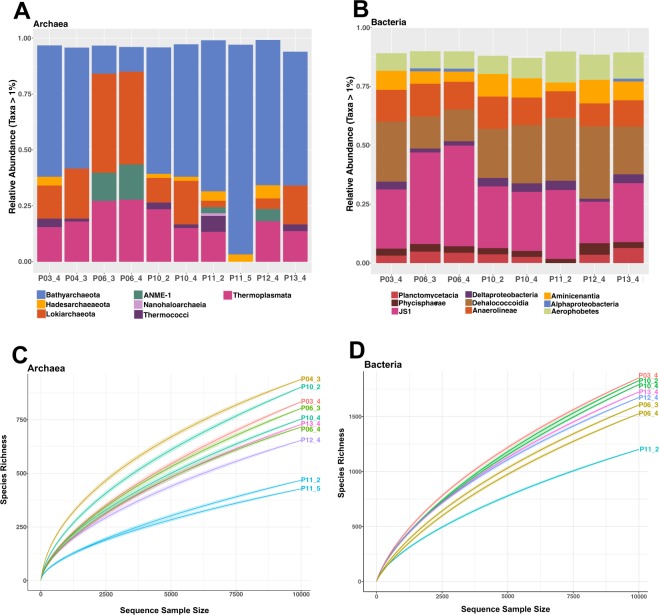
Microbial community composition. (**A**) Archaeal and (**B**) bacterial community summaries for all lineages with >2% sequence abundance, based on 16S rRNA gene amplicons recovered from piston-cored sediments. Sample key: Core P03_4, 2.96–3.01 mbsf; Core P04_3, 2.02–2.07 mbsf; Core P06_3 and P06_4 at 2.25–2.3 and 3.75–3.80 mbsf; core P10_2 and P10_4 at 1.24–1.29 and 3.73–3.78 mbsf; Core P11_2 and P11_5 at 1.15–1.20 and 4.63–4.68 mbsf; Core P12_4 at 3.73–3.78 mbsf; Core P13_4, 3.05–3.10 mbsf. Taxonomic groups are generally shown at the class level, sometimes at the order (ANME-1) or family level (*Phycisphaerae*, *Anaerolineae*). Rarefaction curves for (**C**) archaeal and (**D**) bacterial 16S rRNA amplicons, for the same samples and sequence datasets.

To identify potential microbial catalysts of methane and sulfur cycling, and organic matter remineralization, the bacterial and archaeal communities in sediment samples from cores P10 and P11 were analyzed by high-throughput 16S rRNA gene sequencing (Fig. 6). Lineages of anaerobic heterotrophs dominate these communities; specifically, the bacterial phyla Chloroflexi (*Anaerolineae* and *Dehalococcoidia*) and Atribacteria (OP9/JS1 lineage), and the archaeal phyla Bathyarchaeota and Lokiarchaeota (Fig. 6A,B). Reconstructed genomes of these largely uncultured bacterial and archaeal lineages reveal the diverse, largely heterotrophic metabolisms of these benthic anaerobes^36,37^. Rarefaction analyses show that bacterial and archaeal community richness in the P11 samples are lower relative to all other piston-cored Guaymas sediment sites (Fig. 6C,D).

Archaea and Bacteria central to methane and sulfur cycling in the Ringvent data include methane-oxidizing ANME-1 archaea, and sulfate-reducing Deltaproteobacteria, in particular the family *Desulfobacteraceae*, the *Desulfatiglans* lineage, and an uncultured lineage that taxonomy servers assign to the family *Desulfarculaceae* (Supplementary Figs 9 and 10; Supplementary Data 6). While these methane- and sulfur-cycling anaerobes dominate sequencing surveys in hot hydrothermal sediments of Guaymas Basin^35,38^, they occur here in lower proportions. ANME-1 archaea and deltaproteobacterial sulfate reducers each account for approximately 4% of the sequence dataset in P11, compared to traces (ca. 0.01%) for ANME archaea, and ca. 3% for deltaproteobacterial sulfate reducers in core P10 (Supplementary Data 6). The sulfidic, methane-rich cores P6 and P12 yielded higher percentages of ANMEs, and reduced proportions (0.75 to 1.5%) of deltaproteobacterial sulfate reducers, possibly as a result of sampling below the sulfate-rich zone. The *Desulfobacteraceae* are a physiologically diversified family of sulfate reducers that oxidize low-molecular weight organic acids, acetate, and some aromatic compounds to CO_2_ and occur frequently in marine sediments^39^. In core P11, these sequences include – in small numbers - members of the SEEP-SRB1a cluster^40^, the bacterial syntrophs of ANME methane oxidizers in marine sediments (Supplementary Fig. 10). The *Desulfatiglans* lineage, named for the cultured genus and species *Desulfatiglans anilinii*, includes aromatic-oxidizing isolates and enrichment cultures, and is widespread in marine sediments; subsurface-adapted metabolic capabilities, such as dehalogenation, also characterize this group^41^. The facultatively autotrophic sulfate-reducing family *Desulfarculaceae*^42^ turned out to be unrelated to the Guaymas Basin amplicons that were assigned to this family by web-based taxonomy pipelines; instead, the amplicons formed a distinct phylogenetic lineage with clones from geographically diverse marine subsurface sediments (Supplementary Fig. 10). Interestingly, cultured methanogenic lineages^43^, such as the hydrogenotrophic, autotrophic *Methanocellaceae*, the acetoclastic/methylotrophic *Methanosarcinaceae*, and the methylotrophic *Methermicoccaceae*, account only for small numbers of sequences in all piston-cored Guaymas sediments (Supplementary Data 6), and are unlikely to represent major methane producers in these sediments. Sediment pushcores that were collected by HOV *Alvin* from hydrothermally active sediments at the Ringvent structure (Mound 1 site) and analyzed specifically for methyl coenzyme M reductase (alpha subunit), the key gene of methanogenesis and methane oxidation^44^, yielded ANME-1 sequences (Supplementary Fig. 11), indicating methane-oxidizing microbial populations in the currently active Ringvent mound.

## Discussion

**Figure 7:**
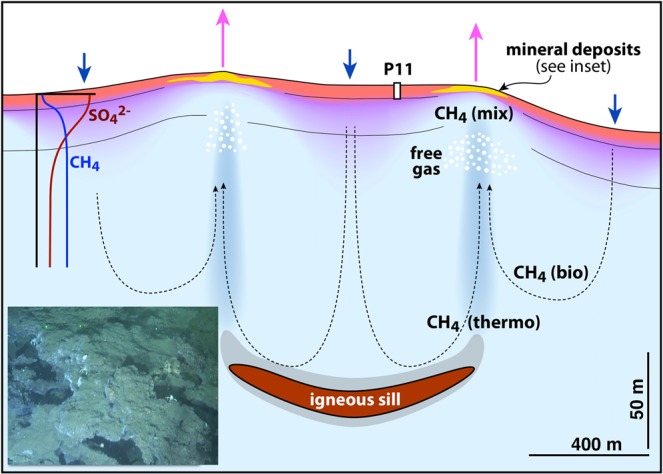
Hydrothermal circulation at Ringvent. Subsurface hydrothermal flow is driven by a gradually cooling but still-active subsurface sill that generates patterns of hydrothermal venting and recharge. Sediments rich in sulfate are indicated in red, methane-rich zones are shown in blue, and sediments with coexisting methane and sulfate are marked in purple. The inset photo shows the porous mineral deposits of the Ringvent mound.

Ringvent provides the first comprehensively characterized example of an active off-axis hydrothermal vent system in Guaymas Basin, at a spreading age of approx. 1.1 million years^1^. Among currently characterized off-axis Guaymas Basin sites, Ringvent provides the clearest example of a sill-associated hydrothermal site that contrasts with seep-like locations on the northern flanking regions^4^. Similar to the Guaymas Basin spreading center^5,6^, seafloor vent features at Ringvent remain spatially congruent with the underlying sill. The gradually cooling sill at ca. 200 m sediment depth sustains subsurface gas accumulation, hydrothermal circulation and upflow focused along the margins of the buried round sill that reaches the seafloor in a ring pattern. Observations of vent fluids of 20–75 °C associated with sulfur-oxidizing microbial mats and sulfur-oxidizing symbiont-dependent *Riftia* tube worms indicate channelized flow of hot hydrothermal fluids with temperatures of at least 75 °C, close to the thermal range that has been measured in deep DSDP boreholes, such as Hole 481 in the northern spreading center of Guaymas Basin^45^. Beyond these localized hot spots, the thermal gradients in sediments within and immediately adjacent to the ring structure are almost an order of magnitude steeper than those in the surrounding seafloor sediments (Supplementary Fig. 2, Table 1). Thus, warm subsurface fluids are likely to impact the entire ring zone (Fig. 7).

In the hydrothermally active southern trough of Guaymas Basin, the mineralogy of active high-temperature and extinct vent structures suggests a paragenetic sequence of mineralization over the life cycle of a vent, with high-temperature deposition of sulfides, followed by moderate-temperature deposition of sulfates, calcite, and barite, and concluding with lower-temperature deposition of amorphous silica^18^. Extinct hydrothermal mounds in southern Guaymas Basin have mineralogical similarities to Ringvent, and analogous silica-dominated mounds and chimneys exist at other hydrothermal sites including the Juan de Fuca Ridge^46^, the Galapagos Ridge^47^, the Central Indian Ridge^48^, and TAG mound in the Trans-Atlantic Geotraverse hydrothermal field^49^. In each of these settings, it is inferred that a silica-rich hydrothermal fluid is conductively cooled, becoming saturated in silica, and variably mixed with seawater followed by precipitation upon further cooling. The temperatures of amorphous silica deposition derived from thermodynamic models and some fluid inclusion studies at analog sites are in the range of ca. 50–150 °C^50^. The consistent recovery of hydrothermal silicates during this and previous sampling at Ringvent^22^ indicates hydrothermal silica mobilization and subsequent precipitation as a characteristic process, with the rapidly deposited diatom-rich sediment of Guaymas Basin as the most likely source^2^. The predominance of silicates, and the absence of hydrothermal sulfides from the available samples constrain the thermal regime within the halo of the sill underlying Ringvent. The maximal temperatures of 315 °C that have been documented for hydrothermal fluids at the southern Guaymas Basin spreading center^51^ are unlikely to occur at Ringvent presently.

The occurrence of silica nodules in subsurface sediments can provide a window into the history of hydrothermal activity, based on the working hypothesis that these nodules were originally deposited near or at the seawater interface, similar to coastal hot spring sinter deposits in Baja California today^17^. The silica nodule at 2.35 m depth in core P11 indicates a previous event of hydrothermal silica mobilization and deposition in the central area of Ringvent. The potential time horizon is rather broad, with a minimum age of 2350 years, based on an approximate sedimentation rate of 1 mm/y for the Sonora Margin slopes just east of Guaymas Basin, or maximally 8217 years ago based on the local ^14^C-derived sedimentation rate for core P11 (Supplementary Data 4). Today, hydrothermal conditions with potential for ongoing silica deposition are found on the ring mound in localized hotspots, sometimes within small depressions and gullies that may have originated during episodes of increased hydrothermal activity.

A history of variable hydrothermal conditions is also evident from the sedimentary carbonates in core P11. Currently, methane with a distinctive δ^13^C isotopic signature between biogenic seep methane and hydrothermal Guaymas Basin methane permeates the sediment column. The oxidant-replete conditions in the piston-cored sediment column indicate that this methane is not produced *in-situ*, but appears to have a subsurface source, consistent with a mixed thermogenic/microbial δ^13^C composition (Fig. 7). This methane flux supports methane-oxidizing archaea that form a minor component within the predominantly heterotrophic sedimentary microbial community (Fig. 6), but do not produce enough methane-derived DIC to be visible in δ^13^C-DIC profiles or in contemporary formation of ^13^C-depleted carbonates at the sediment surface. Yet, strongly ^13^C-depleted, partially methane-derived carbonate nodules appear in the upper sediment column and indicate a relatively recent, strong methane flux, and a larger methane-derived DIC pool. In other words, the ^13^C-depleted carbonates in core P11 preserve a paleosignal of methane oxidation to DIC and subsequent incorporation into carbonates that contrasts with minimally δ^13^C-depleted porewater DIC today. By applying a 1 mm/y sedimentation rate from the Guaymas Basin slopes, the depth of the carbonate nodule layer at 1 to 1.20 m translates into an age of 1000 to 1200 years; local ^14^C-derived sedimentation rates for core P11 (Supplementary Data 4) yield an age between ca. 4500 to 6700 years. Shallower, methane-imprinted nodules persist in core P11 until ca. 0.5 m depth, and indicate that methane flux and methane-derived carbonate precipitation have slowed down gradually. These timelines imply that carbonate nodules should have formed at former sediment surfaces where sulfate would have been available, and not later within the sediment column.

Today, the concentration and isotope profiles of porewater sulfate, sulfide and DIC show that, of all Guaymas Basin sampling locations, the Ringvent core is most strongly influenced by seawater. In hydrothermal areas, seawater inmixing is commonly connected to hydrothermal circulation via recharge. We hypothesize that evolving hydrothermal circulation has changed the central basin of Ringvent from an active hydrothermal area characterized by silica mobilization and methane venting, to a seawater-influenced recharge zone where seawater is mixed into methane-rich subsurface fluids (Fig. 7). We propose an evolutionary sequence starting with hot, freshly emplaced sills driving intense hydrothermal circulation, silica mobilization at 100–200 °C^18^, followed by precipitation of seafloor silicate minerals, gradually cooling conditions that allow for sulfate-dependent microbial methane oxidation within a thermal limit ca. 80 °C^19^, and deposition of methane-imprinted carbonates, as seen in the sediment column of core P11.

The microbial 16S rRNA gene sequencing results show that the key lineages of microbial methane and sulfur cycling, ANME archaea and deltaproteobacterial sulfate reducers, are less conspicuously present in core P11 than in active hydrothermal sediments of Guaymas Basin, where they often dominate the archaeal and bacterial communities^19,38^. Detecting ANME-1 sequences in core P11 indicates potential for sulfate-dependent methane oxidation, but with the caveat that DNA data do not provide a quantitative proxy for current levels of cellular activity and process rates. In a conservative interpretation, these sequence signatures would indicate a minor component of the sedimentary microbial community, potentially a remnant population of a methane-oxidizing and sulfate-reducing hydrothermal community that is now largely replaced by heterotrophic bacteria and archaea that are common in marine subsurface sediments. At present, the non-sulfidic conditions in the P11 sediment column are likely to attenuate anaerobic methane-oxidizing activity. The conspicuously reduced species richness in core P11 (Fig. 6) suggests that disturbances have purged these microbial communities, for example strong methane seepage or hydrothermal activity that commonly reduce microbial diversity in marine sediments^35,52^. So far, species richness in this core has not fully recovered, and appears impoverished in particular by comparison to core P10, obtained nearby but outside of Ringvent.

Drawing on the available evidence to infer evolutionary scenarios for sill-driven hydrothermal activity, Ringvent provides an instructive example for the hydrothermal stage of an off-axis site transitioning into a cold-seep-like system. It is likely that most sill-hosted systems will transition into cold seeps that persist for a considerable time, since high-permeability conduits will continue to advect heat from deep fluids after the host sill is cool and flow is primarily compaction-driven. It is thus not surprising that seep-like sites are more commonly detectable than shorter-lived hydrothermally active sites^4^. In addition, it is possible that sills intruding at great depth within thick sediments, such as those close to the transform fault that separates Guaymas Basin from the Sonora Margin, cool more slowly due to greater insulation and the inefficiency of hydrothermal flow to those depths. These sites, appearing as cold seeps, may have greater longevity than shallow-sill sites and also mine a larger subsurface methane reservoir. Such cold seep sites in Guaymas Basin carry a signature of their hydrothermal origin: δ^13^C-CH_4_ signatures are distinctly heavier than those for biogenic methane on the Sonora Margin, and fall into the mixed thermogenic/biogenic range that is expected for methane in sill-impacted sediments^4^.

This scenario for an evolving Ringvent system should be tested further. For example, subsurface drilling at off-axis sites should uncover geochemical and microbial evidence for hydrothermalism^53^ which increasingly resembles the impact of fully developed venting at spreading centers, as depth and proximity to the heat source increase. Deep drilling at Ringvent should constrain the patterns of hydrothermal circulation and recharge, and allow the development of a well-constrained chronology for hydrothermal activity. A deep-sea drilling cruise to Guaymas Basin (IODP Expedition 385) is targeting Ringvent to comprehensively investigate the subsurface foundations for off-axis hydrothermal venting in Guaymas Basin.

## Methods

### Field surveys

Ringvent was initially imaged with sidescan sonar backscatter and seafloor towcam with MCS profiles, in the context of shallow seismic and photographic seafloor surveys of the Guaymas Basin flanking regions^9^, as detailed in Supplementary Materials. In October 2014, R/V *El Puma* (Chief scientist, Carlos Mortera, Instituto de Geofísica, Universidad Nacional Autónoma de México) performed a detailed bathymetric survey of the extended Guaymas Basin region and collected sediment piston cores of 4 to 5 m length. A 5-m long piston core (P11) was obtained on Oct 21, 2014 from the central basin within the ring (27°N30.5090/111°W40.6860, 1749 m; core length 4.9 m), parallel to a control core (P10) approx. 1 mile to the west of Ringvent (27°N30.5193/111°W42.1722; 1731 m depth, 3.93 m core length) collected on the same day. Core P06 was obtained on Oct. 19 from the lower Sonora Margin, near its boundary with Guaymas Basin (27°N38.8367/111°W36.8595; 1681 m depth, 3.95 m core length); Core P12 was taken on Oct. 22 from the upper Sonora Margin in the oxygen minimum zone, near DSDP site 480 (27°N52.1129/111°W41.5902, 667 m, 4 m core length). Cores P03 and P04 were both collected on Oct. 17 from the northwestern end of Guaymas Basin (27°N37.6759/ 111°W52.5740; 1611 m depth, 3.27 m core length and 27°N39.2740/ 111°W52.9950, 1627 m depth, 3.47 m core length, respectively). Core P13 was obtained on Oct. 22 from the southeastern Guaymas Basin (27°N12.4470/111°W13.7735, 1859 m depth, 3.31 m core length). Geochemically, cores P03, P04 and P13 are similar to core P10. Core positions in Guaymas Basin are plotted in Supplementary Figs 1 and 2.

During expedition AT37–06 to Guaymas Basin (December 7 to 27, 2016). AUV *Sentry* and submersible *Alvin* mapped and sampled Ringvent. Photo coverage of the *Alvin* Ringvent dives 4864 and 4865 is available at the *Alvin* framegrabber site [http://4dgeo.whoi.edu/Alvin]. The CHIRP subbottom and bathymetry survey by *Sentry* was run at a survey height of ca. 65–70 meters above bottom during *Sentry* dive 410; the seafloor photomosaic survey was performed during *Sentry* dive 411 ca. 6 meters above bottom. Redox anomalies in the water column were recorded with Sentry’s ORP [δorp/δt] sensor, which measures the time derivative of oxygen reduction, a proxy for the presence of chemically active substances that react with oxygen. The ORP tool was developed at NOAA-PMEL for the mapr instrument (https://www.pmel.noaa.gov/eoi/PlumeStudies/mapr/).

During the R/V *Atlantis* survey in December 2016, thermal profiles were measured in the surficial sediment using *Alvin’s* 1-meter heatflow probe (https://ndsf.whoi.edu/alvin/using-alvin/sampling-equipment/) that contains thermal sensors every 20 cm, starting 10 cm under the attached plastic disk that limits probe penetration and rests on the seafloor once the probe was inserted. Thermal readings were taken for approx. 5 to 10 minutes until the temperature readings had stabilized. Seafloor mineral samples and shallow sediment push cores were collected during *Alvin* dives 4864 and 4865 in December 2016. Ringvent observations by *Sentry* were made during dives 410 and 411, on December 15 and 16, 2016.

### Porewater chemistry

Porewater was obtained from freshly collected sediments on RV *El Puma* by centrifuging ca. 40 ml sediment samples in 50 ml conical Falcon tubes for ca. 5 to 10 minutes, using a Centra CL-2 Tabletop centrifuge (Thermo Scientific) at approx. 1000 *g*, until the sediment had settled and produced ca. 8 to 10 ml of porewater. For porewater sulfate measurements, 1 ml subsamples of the overlying porewater were drawn into syringes and injected through 0.45 μm filters into screwcap Eppendorf vials, each acidified with 50 µl of 50% HCl, and then gently bubbled with nitrogen for 4 min to remove sulfide; the samples were then stored at 4 °C before shipping and analysis. These samples were used for barimetric sulfate quantifications and subsequent determinations of δ^34^S isotopic values at the EaSI lab, University of Texas at El Paso. Barium sulfate (BaSO_4_) was collected via the addition of BaCl_2_ and subsequent centrifugation. The BaSO_4_ was transferred into a pre-weighed 2.5 ml sample tube and was twice re-suspended with 2 ml of deionized water followed by centrifugation to remove dissolved salts such as NaCl. The samples were dried, and the weight of the sample was obtained from the difference between tube weight with sample and empty tube weight. For sulfur isotope analysis, ~0.45 mg of BaSO_4_ and associated standards (NBS-127, δ^34^S = +21.1‰, IAEA-SO-5, δ^34^S = +0.49‰, and IAEA-SO-6, δ^34^S = −34.1‰) were weighed into tin capsules with an approximately equal amount of vanadium pentoxide (V_2_O_5_). The samples were analyzed using an Elementar^®^ Pyrocube, connected to a GEOVisION^®^ isotope ratio mass spectrometer. The standard deviation (1σ) for replicate sulfur isotope measurements of standards was 0.1‰. All sulfur isotope values are reported in per mil relative to Vienna Canyon Diablo Troilite (VCDT). For porewater sulfide analysis, 1 ml porewater subsamples were drawn into syringes, filtered immediately through 0.45 μm filters, and placed in Eppendorf sample vials each containing 0.1 ml of 0.1 M zinc acetate solution to preserve the sulfide as zinc sulfide until analyzed. Sulfide was quantified spectrophotometrically at UNC-Chapel Hill using the methylene blue method^54^. Filtered but unamended porewater samples, also stored at 4 °C, were used for quantifying multiple stable ions, including sulfate, by ion chromatography at GEOMAR, Kiel, Germany^55^.

To measure concentrations and δ^13^C signatures of dissolved inorganic carbon (DIC), 2 ml of unamended porewater from each sediment horizon were injected into evacuated serum vials (30 ml) and stored upside down at −20 °C. After the cruise, the samples were acidified with phosphoric acid, and measured by GC-IRMS as described^56^. For combined concentration and δ^13^C analysis of methane, 2 ml sediment subsamples were added to 30 ml serum vials containing 2 ml of 1 M sodium hydroxide solution, sealed with thick butyl rubber stoppers, crimped with aluminum seals and stored at 4 °C. Since cores were retrieved unpressurized, outgassing may have impacted in particular the measurements of methane concentrations near and above saturation, 1.5 mM; however, no gas cavities were observed. After the cruise, the methane samples were analyzed by headspace gas chromatography-flame ionization detection (GC-FID) at Florida State University^57^. Gas samples were analyzed for δ^13^C by injecting 0.1 to 0.5 ml of sample into a gas chromatograph interfaced to a Finnigan MAT Delta S isotope ratio Mass Spectrometer inlet system as previously described^58^. Small amounts of gas were cryo-concentrated before isotopic measurements. Values are reported in the per mil (‰) notation relative to Vienna Pee Dee Belemnite (VPDB).

### Sediment geochemistry

Samples selected for radiocarbon analyses were freeze-dried, homogenized, and acidified to remove CaCO_3_, allowing for the analysis of remaining organic matter, and preventing the distortion of radiocarbon ages by methane-derived carbonates. Acidification was performed on ~200 mg of sample, which was treated with ~5 ml buffered pH 5 acetic acid solution for ~24 hours to dissolve the CaCO_3_. Samples were then rinsed with Milli-Q water 6 to 8 times to remove the acetic acid. Acidified samples were then freeze-dried again, re-homogenized and stored for ^14^C and ^13^C analysis. Radiocarbon dating was performed at Lawrence Livermore National Laboratory Center for Accelerator Mass Spectrometry, and a reservoir age of 406 years was used before conversion to calendar years using CALIB REV7.1.0. [http://calib.org]. The δ^13^C values of all the acidified samples used for radiocarbon analyses were approximately −20 to −22‰, as expected for marine primary producers-derived organic matter. Samples selected for carbonate isotope analyses were freeze-dried, homogenized, and roasted under vacuum to eliminate organic matter. Isotope analyses was performed on ~500 µg of sediment using a Kiel devise coupled with a Thermo MAT 253 gas ratio mass spectrometer at the University of California, Santa Cruz. Values are reported in the per mil (‰) notation relative to Vienna Pee Dee Belemnite (VPDB). Reproducibility of in-house standards is 0.07‰ for δ^18^O and 0.03‰ for δ^13^C.

### Mineral analyses

Ringvent samples collected at the seafloor by *Alvin* were studied at UNAM using a transmitted light microscopy (Olympus BX60) coupled with a Motic camera and a low-vacuum Hitachi TM-1000 table-top scanning electron microscope. Stable isotope geochemistry from fibrous aragonite cement samples, and the bivalve shell from Ringvent core P11, were analyzed at UNAM using a mass spectrometer Thermo Finnigan MAT 253 coupled with Gas Bench II, following published guidelines^59^. Bulk mineralogy of seafloor samples was determined via X-ray diffraction (XRD) using an EMPYREAN diffractometer equipped with a fine focus Cu tube, nickel filter, and PIXCell 3D detector operating at 40 mA and 45 kV at UNAM. For this, samples were ground with an agate pestle and mortar to <75 μm and mounted in back-side aluminum holders. The analyses were carried out following previously published procedures^22^. Phase identification was made with PDF-2 and ICSD databases. Mineral phases from core P11 were analyzed separately with a Rigaku Smart Lab XRD using 0.003° resolution, 5°/minute using a ICDD PDF4+ 2019 database (Ivano Aiello, Moss Landing Marine Laboratory).

### DNA extraction, PCR and Sequencing

DNA was extracted from 0.5 to 1 ml sediment samples of the *El Puma* cores (Core P03–4, 2.96–3.01 mbsf; Core P04–3, 2.02–2.07 mbsf; Core P06–3 and P6–4 at 2.25–2.3 and 3.75–3.80 mbsf; core P10-2 and P10-4 at 1.24–1.29 and 3.73–3.78 mbsf; Core P11-2 and P11-5 at 1.15–1.20 and 4.63–4.68 mbsf; Core P12-4 at 3.73–3.78 mbsf; Core P13-4, 3.05–3.10 mbsf) using the Powersoil DNA extraction kit according to the manufacturer’s instructions (QIAGEN, Carlsbad, CA, USA). The v4v5 regions of the 16S rRNA gene were amplified from the DNA extractions separately for bacteria and archaea, and prepared for Illumina sequencing through the Census of Deep Life at the Marine Biological Laboratory, Woods Hole. Bacterial amplification was carried out as previously described^60^ using forward primer 518F (5′-CCAGCAGCYGCGGTAAN-3′) in combination with three versions of reverse primer 926R (5′-CCGTCAATTCNTTTRAGT-3′, 5′-CCGTCAATTTCTTTGAGT-3′, 5′-CCGTCTATTCCTTTGANT-3′). The archaeal v4v5 16S rRNA gene was targeted by a combination of five variants of forward primer 517F (5′-GCCTAAAGCATCCGTAGC-3′; 5′-GCCTAAARCGTYCGTAGC-3′, 5′-GTCTAAAGGGTCYGTAGC-3′, 5′-GCTTAAAGNGTYCGTAGC-3′, 5′-GTCTAAARCGYYCGTAGC-3′) and a single reverse primer 958R (5′-CCGGCGTTGANTCCAATT-3′)^61^. 16S rRNA amplicon sequencing was performed using an Illumina MiSeq Benchtop sequencer (Illumina, San Diego, CA, USA) at the Marine Biological Laboratory in Woods Hole, MA as described on the Visualization and Analysis of Microbial Population Structures (VAMPSs) website (https://vamps.mbl.edu/resources/primers.php).

### Sequence analyses

To complement taxonomy assignments in VAMPS and to further explore the taxonomic identifications of uncultured groups, archaeal and bacterial sequences were processed with *mothur* v.1.39.5^62^ following the *mothur* Illumina MiSeq Standard operation procedures^62^. Briefly, forward and reverse reads were merged into 1.6 million contigs (746,180 Archaeal and 831,101 Bacterial) and selected based on primer-specific amplicon length and the following parameters: maximum homopolymer length of 6 and no base ambiguities. Subsequently, 698,385 Archaeal and 763,463 Bacterial high-quality sequences of ca. 330 nucleotides length were aligned against the *mothur*-recreated Silva SEED v132 database and pre-clustered at 1% dissimilarity. As previously suggested^63^, spurious sequences are mitigated by abundance ranking and merging of rare sequences based on minimum differences of three base pairs. Chimeras were detected and removed using UCHIME de novo mode^64^. Sequences were then clustered, by generating a distance matrix using the average neighbor method, into operational taxonomic units (OTUs, 97% or greater sequence similarity cutoff). OTU classification was performed on *mothur* using the SILVA v132 database. Community analyses were performed on subsampled datasets (*n* = 64,890 sequences per sample for Archaea and *n* = 82,728 sequences per sample for Bacteria). Community structure visualizations and rarefaction analyses were generated using the *vegan* and *phyloseq* R-packages^65,66^.

To examine pipeline-derived phylogenetic assignments among methanogenic, methane-oxidizing and sulfate-reducing microbial populations, including hard-to-place uncultured lineages, representative sequences were placed into phylogenetic trees. Pairs of sequenced amplicons were merged by the QIIME function Fastq-join^66^. Representative OTUs were populated using the *de novo* method in QIIME with a 97% sequence similarity cutoff^67^. After noting the sequence population for every OTU, representative sequences from OTUs containing at least ten sequences were used for downstream phylogenetic analyses, and are listed in Supplementary Materials for reference (Supplementary Data 7). Singletons and chimeric sequences were excluded from analysis using ChimeraSlayer^68^. The Greengenes rRNA database was used for OTU taxonomic assignment^69^. Phylogenetic trees for representative OTUs were inferred with the program package PAUP4.0, using Maximum likelihood distances, transition and transversion rates estimated for each alignment, and Minimum Evolution as optimality criterion^70^. Branching patterns were checked with 1000 bootstrap reruns.

For functional gene analysis, *mcrA* genes were amplified forward and reverse primers (5′-GACCAGTTGTGGTTCGGAAC-3′; 5′-ATCTCGAATGGCATTCCCTC-3′) for ANME1 and related archaea^43^. The PCR cycle started with initial denaturation at 95 °C for 1 minute, followed by 30 cycles of denaturation at 95 °C, annealing at 55 °C, and extension at 72 °C of 1 minute each, and concluded by a final 5-minute extension at 72 °C. PCR products were purified using the Wizard SV Gel and PCR Cleanup System (Promega Corporation, Madison, WI, USA) and cloned into plasmid vectors using the TOPO TA Cloning Kit (Life Technologies, Carlsbad, CA, USA). One Shot TOP10 *E. coli* cells (Life Technologies, Carlsbad, CA, USA) were transformed with the vectors and plated on selective media. Colonies were picked and incubated overnight in SOC medium. Plasmid DNA was extracted using the GeneJET Plasmid Miniprep Kit (Thermo Fisher Scientific, Waltham, MA, USA), and sequenced at GeneWiz (South Plainfield, NJ, USA). Using the MEGA software package, sequences were aligned using the MUSCLE algorithm and the tree was inferred using the neighbor joining method^71^.

## Supplementary information

Supplementary Figures 1–11 and Datasets 1 to 7

## Data Availability

All partial 16S rRNA sequence data obtained at MBL are publically available on the NCBI SRA repository (BioProject: PRJNA420722; SRA experiment accession entrees SRX3440273-SRX3440290). For easy reference and data recovery, the partial 16S rRNA sequences selected for phylogeny construction are included in Supplementary Materials. The methyl coenzyme M reductase alpha subunit gene sequences are deposited at GenBank under accession numbers MH931022 to MH931084. Geochemical data are available at the BCO-DCO under these reference links: Porewater methane data: https://www.bco-dmo.org/dataset/661750/data Porewater sulfate data: https://www.bco-dmo.org/dataset/661775/data Porewater DIC data: https://www.bco-dmo.org/dataset/661658/data Porewater sulfide: https://www.bco-dmo.org/dataset/661808/data
