# Supplementary material for: Characteristics and Evolution of sill-driven off-axis hydrothermalism in Guaymas Basin – the Ringvent site
Source: Sci Rep. 2019 Sep 25;9:13847. doi: 10.1038/s41598-019-50200-5 (PMC6761151; doi:10.1038/s41598-019-50200-5)
Supplement: Supplementary file 1 — Supplementary Figures 1–11 and Datasets 1 to 7 [file 41598_2019_50200_MOESM1_ESM.pdf]

Supplementary Materials

Characteristics and Evolution of sill-driven off-axis hydrothermalism in Guaymas Basin

– the Ringvent site

Andreas Teske, Luke J. McKay, Ana Christina Ravelo, Ivano Aiello, Carlos Mortera, Fernando Núñez-Useche, Carles Canet, Jeffrey P. Chanton, Benjamin Brunner, Christian Hensen, Gustavo A. Ramírez, Ryan J. Sibert, Tiffany Turner, Dylan White, Christopher R. Chambers, Andrew Buckley, Samantha B. Joye, S. Adam Soule, Daniel Lizarralde

Supplementary Materials

Previous Ringvent surveys. Ringvent was first detected when a 2D, NW-SE trending multi-channel seismic (MCS) profile acquired during R/V *Maurice Ewing* cruise EW0210 in 2002 passed ca. 200 m southwest of the ring structure and showed a shallow reflection, later identified as an out-of-plane reflection from a gas horizon under the ring, overlying the uppermost of several sills that are rising in sequence. During a subsequent cruise in 2009 (R/V *Atlantis*, AT15-54), deep-towed sidescan sonar backscatter revealed a ring-shaped (~800 m diameter) pattern of high backscatter, complemented with near-bottom photography at one location (*Lizarralde et al.,* 2011). Ringvent was further explored with R/V *El Puma* (October 7-27, 2014), R/V *Sonne* (June 23 to July 24 2015) and R/V *Alpha Helix* (May13 to June 3, 2016). In addition to the gravity cores obtained during the El Puma cruise, the RV *Sonne* cruise (Chief Scientist, Christian Berndt, GEOMAR) collected short sediment cores and seafloor grab samples, which recovered silicate (diatomaceous sinter) and carbonate (fine-grained aragonite) minerals at Ringvent (*Núñez-Useche et al.,* 2018). The RV *Alpha Helix* cruise (Chief scientist, Antonio González Fernándo, CICESE) obtained a new set of MCS profiles across the circular seafloor structure; the profiles AH01-02 and AH21-22 running parallel northwest to southeast intersected at right angles with profile AH26-27, with crossing points located just southwest and within the circular structure. These profiles indicated free gas concentrated beneath the ring ~100 m below the seafloor (mbsf) and an underlying sill horizon at ~200 mbsf. The accumulating evidence for sill-driven gas flow at Ringvent prompted *Alvin* dives at this location during R/V *Atlantis* Expedition AT37-06 in December 2016, when extraneous factors disrupted ongoing work at the southern hydrothermal spreading center of Guaymas Basin and alternate dive sites had to be improvised quickly.

Supplementary References

Lizarralde, D., Soule, A., Seewald, J., and Proskurowski, G. Carbon release by off- axis magmatism in a young sedimented spreading centre. *Nature Geoscience* **4**, 50–54 (2011).

Núñez-Useche, F., Canet, C., Liebetrau, V., di Puig, T., Ponciano, A.C., Alsonso, P., Berndt, C., Hensen, C., Mortera-Gutierrez, C., and Rodríguez-Díaz, A. A. Redox conditions and authigenic mineralization related to cold seeps in central Guaymas Basin, Gulf of California. *Marine and Petroleum Geology* **95**, 1-15 (2018).

Supplementary figures

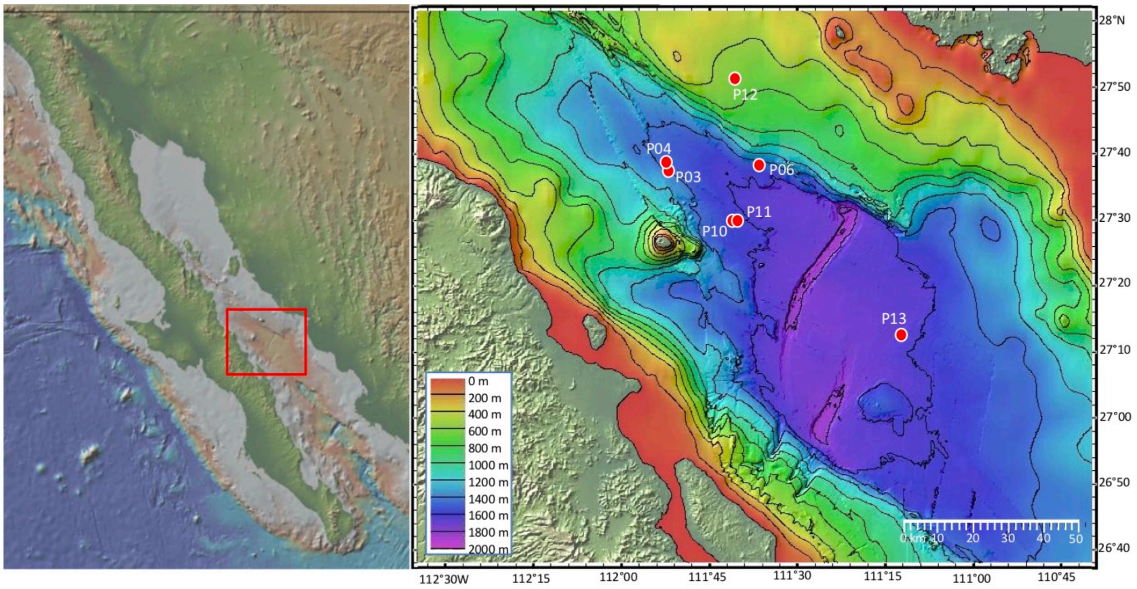

Supplementary Figure 1. Location of Guaymas Basin in the Gulf of California (red rectangle in left panel), and location of El Puma coring sites in the bathymetric map of Guaymas Basin (right panel). The northern and southern spreading centers and their axial troughs reach depths of 2000 m, marked in purple, and are surrounded by extensive ridge flanks in the Northwest and Southeast that gradually rise to ca. 1600 m depth.

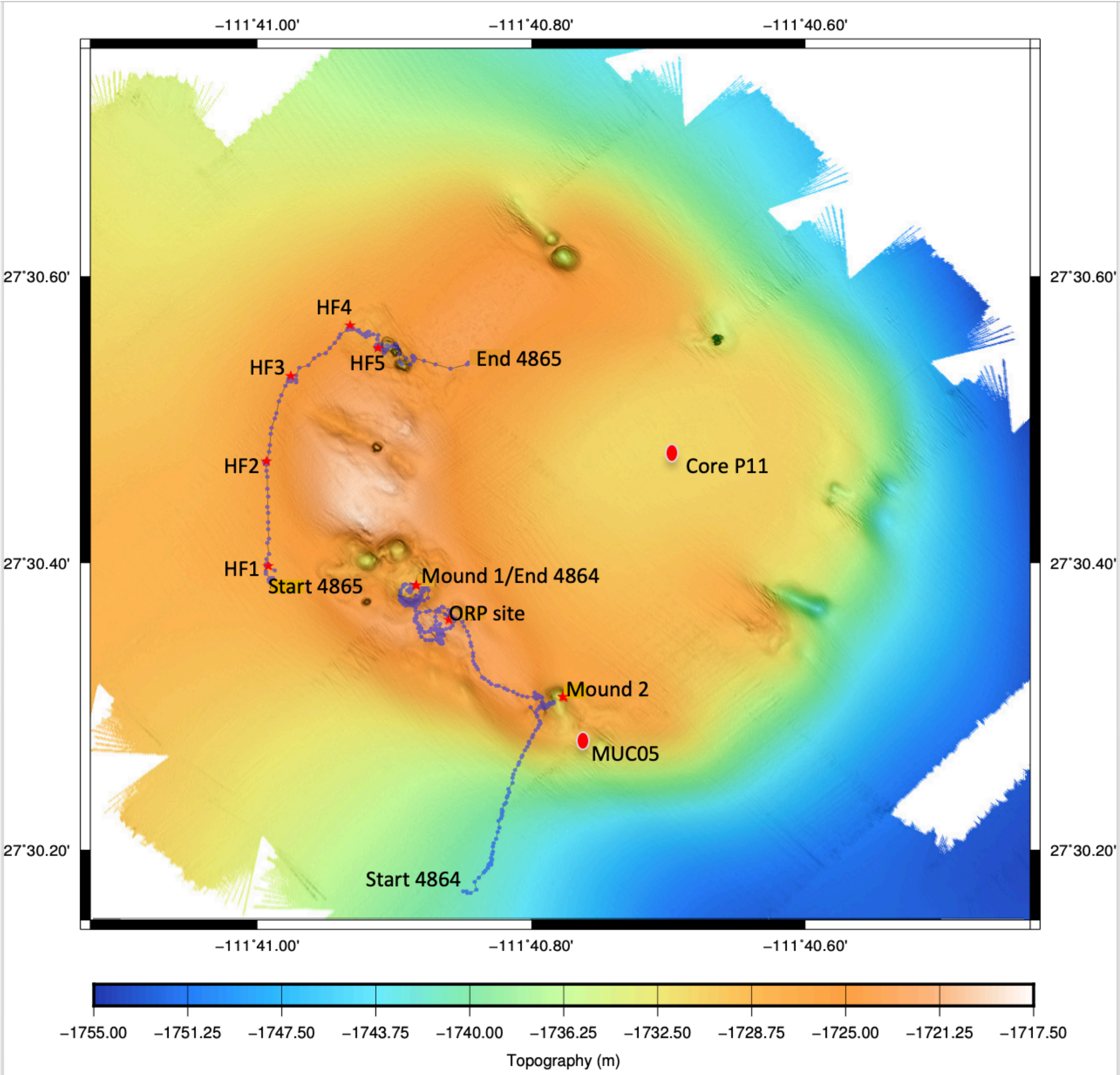

Supplementary Figure 2. Contour-shaded bathymetry from *Sentry* dive 410 annotated with *Alvin* sampling locations, *El Puma* core P11 location, the location of MUC05 (*Geilert et al*. 2018), *Alvin* dive tracks, and thermal profiling locations during *Alvin* dives 4864 and 4865. *Alvin* push cores 4864-10 and 4864-12 were taken at the Mound 1 site. Seafloor mineral samples shown in Figure 3 and Supplemental Figure 6 were collected at the ORP and Mound 1 sites.

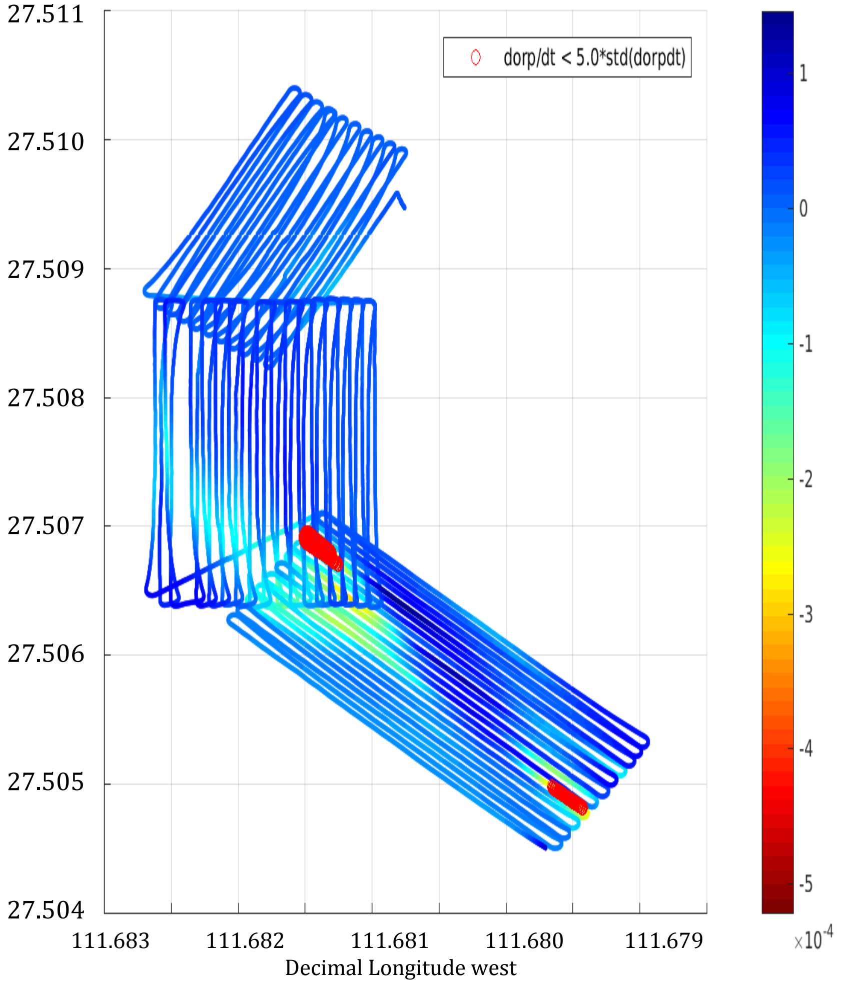

Supplementary Figure 3. ORP sensor data [δorp/δt] measured ca. 6 m above the seafloor during *Sentry* Dive 411. The grid covers the western half of Ringvent. Comparison with the bathymetric map in Supplementary Figure 2 shows that the anomalies are located near the Mound 2 area at the southern edge, and at the ORP site and Mound 1 area on the southwestern ring segment. Grid positions are given in decimal latitude and longitude.

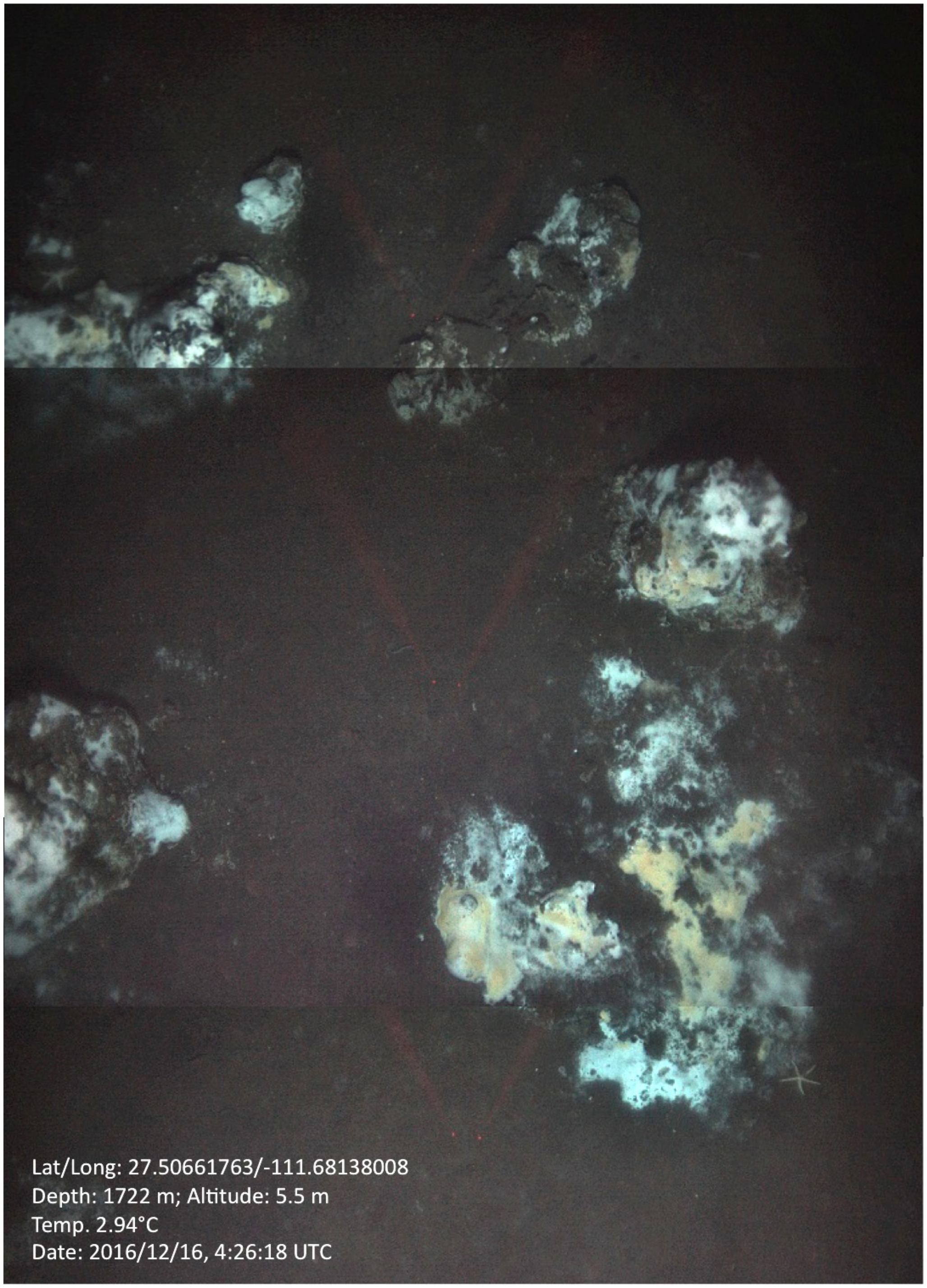

Supplementary Figure 4A. Photomosaic of white and yellow sulfur-oxidizing bacterial mats on sediment and small rocky outcrops, from *Sentry* dive 411 on the western ridge of Ringvent, performed at night between *Alvin* dives 4864 and 4865. Three adjacent overlapping photos were fitted together for this panel.

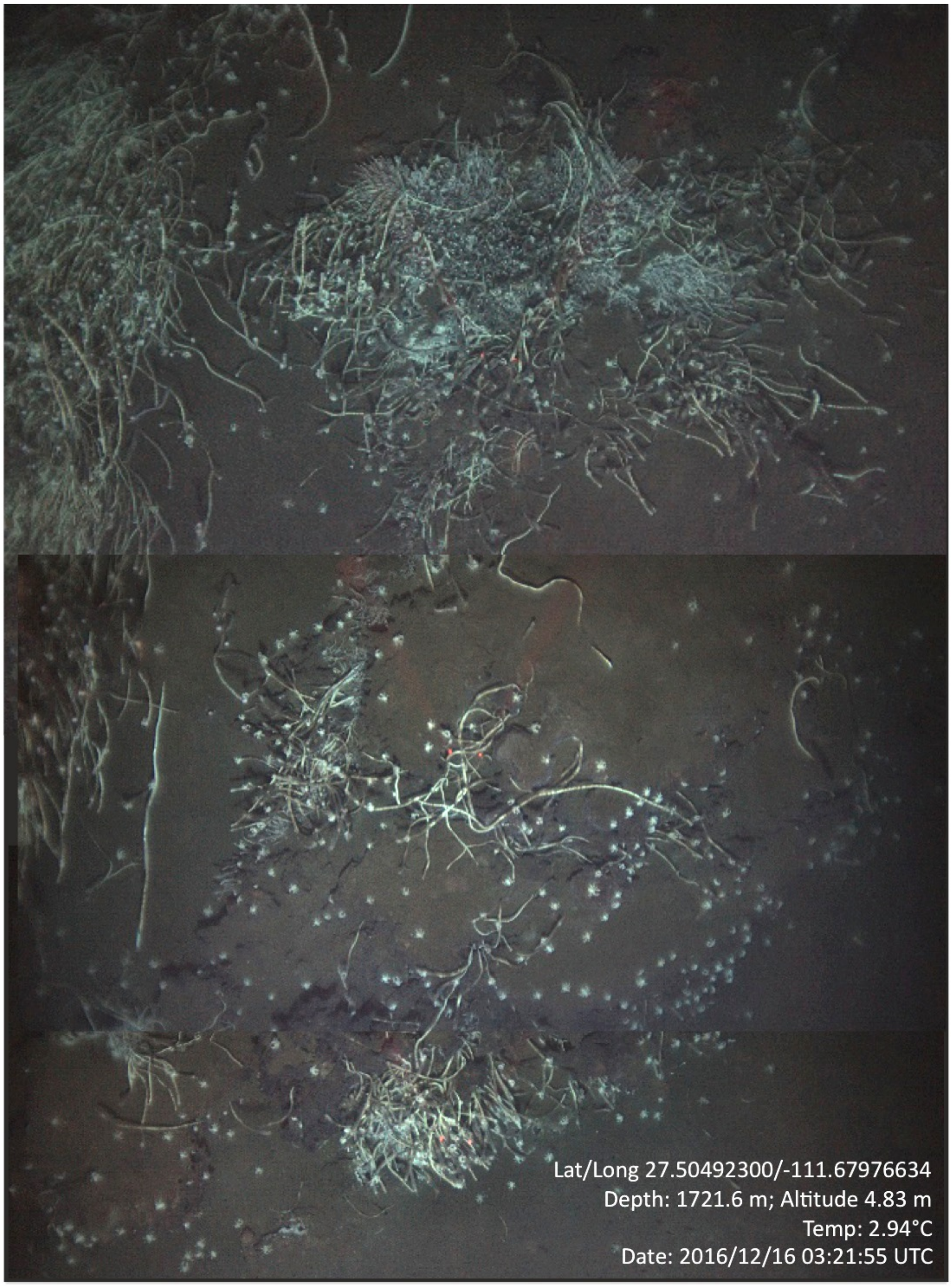

Supplementary Figure 4B. Photomosaic of tubeworms and small crabs associated with cracks in rocky surface, from *Sentry* dive 411 on the western ridge of Ringvent, performed at night between *Alvin* dives 4864 and 4865. Three adjacent overlapping photos were fitted together for this panel.

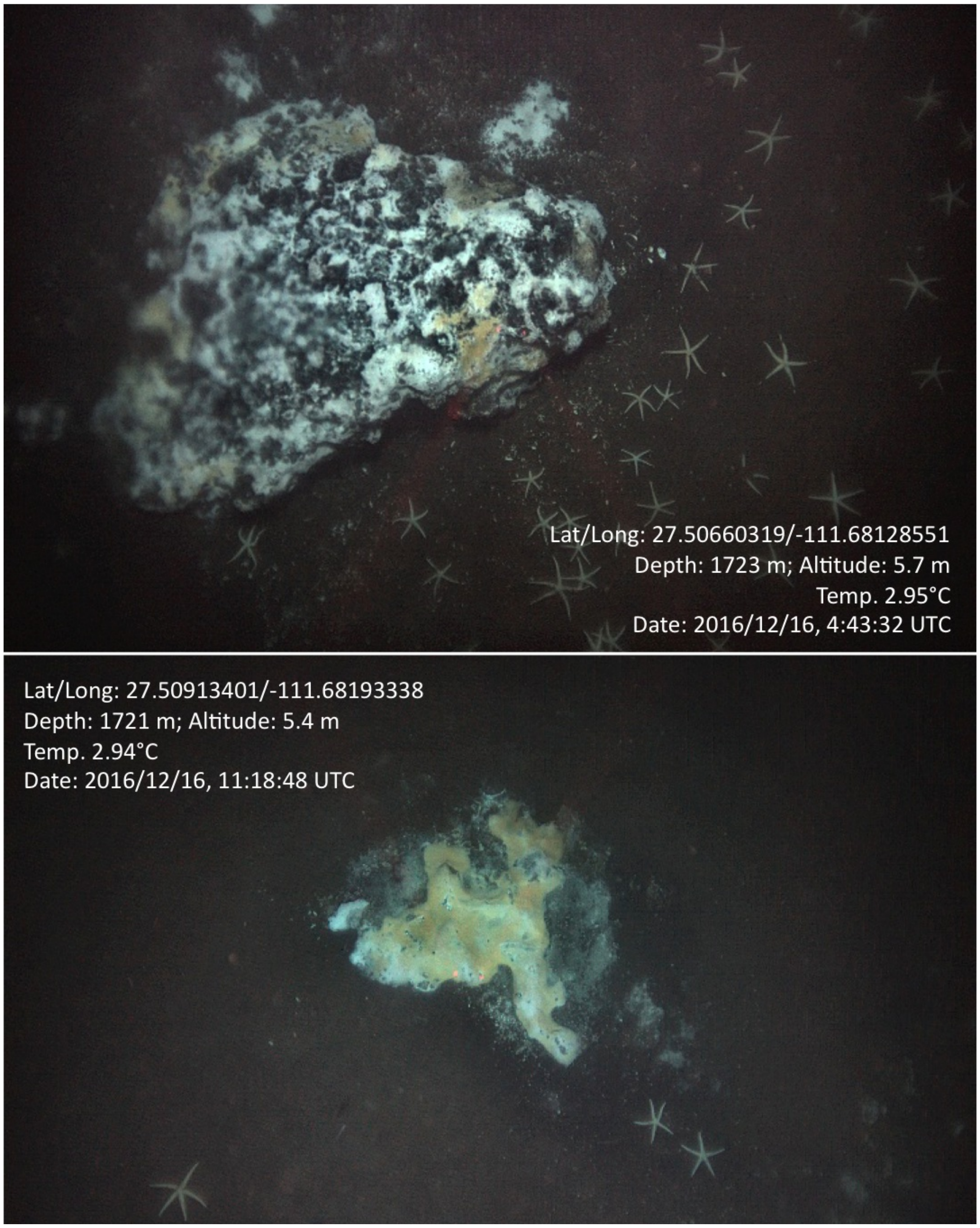

Supplementary Figure 4C. Photos of mat-covered rocky outcrop (top) and sediment (bottom), from *Sentry* dive 411 on the western ridge of Ringvent, performed at night between *Alvin* dives 4864 and 4865. The sediment mat was thermally profiled during *Alvin* dive 4865, showing *in-situ* temperatures near 70°C beginning at ca. 30 cm depth (Temperature profile H5 in Data Table 1).

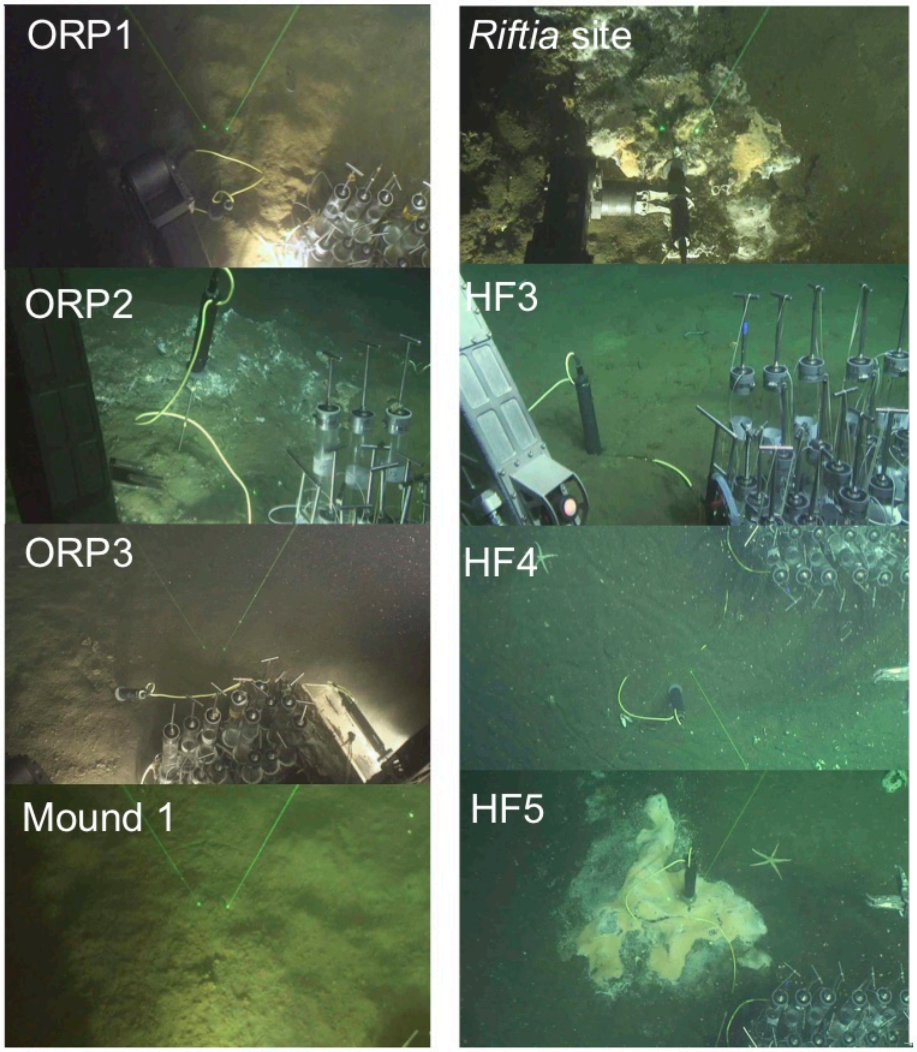

Supplementary Figure 5. In-situ photos and detailed context of heatflow measurements. Thermal readings were taken after equilibration of 5-10 minutes. At ORP 1, the sediment wobbled jello-like with every movement of *Alvin's* heatflow probe and cores, and contained amphetarid polychaetes. At ORP 2, silicate deposits blocked the full insertion of the probe into sediment. *Alvin* backed up ca. 5 meters, until the probe could be fully inserted into soft sediment (ORP3). The Mound 1 profile was measured in a sedimented area with polychaete tubes. At the *Riftia* site near ORP, the probe could not be fully inserted into the brittle underlying mineral substrate, and the top temperature sensor was located ca. 5 cm above the seawater interface. The near-identical profiles HF1 to 3 (Dive 4865) were measured in seafloor sediment on the western periphery of Ringvent; HF4 was obtained in a clam field near a small valley leading into the mound, and HF5 was measured in white-orange *Beggiatoa* mat within this cleft during dive 4865 (Supplementary Figure 4C).

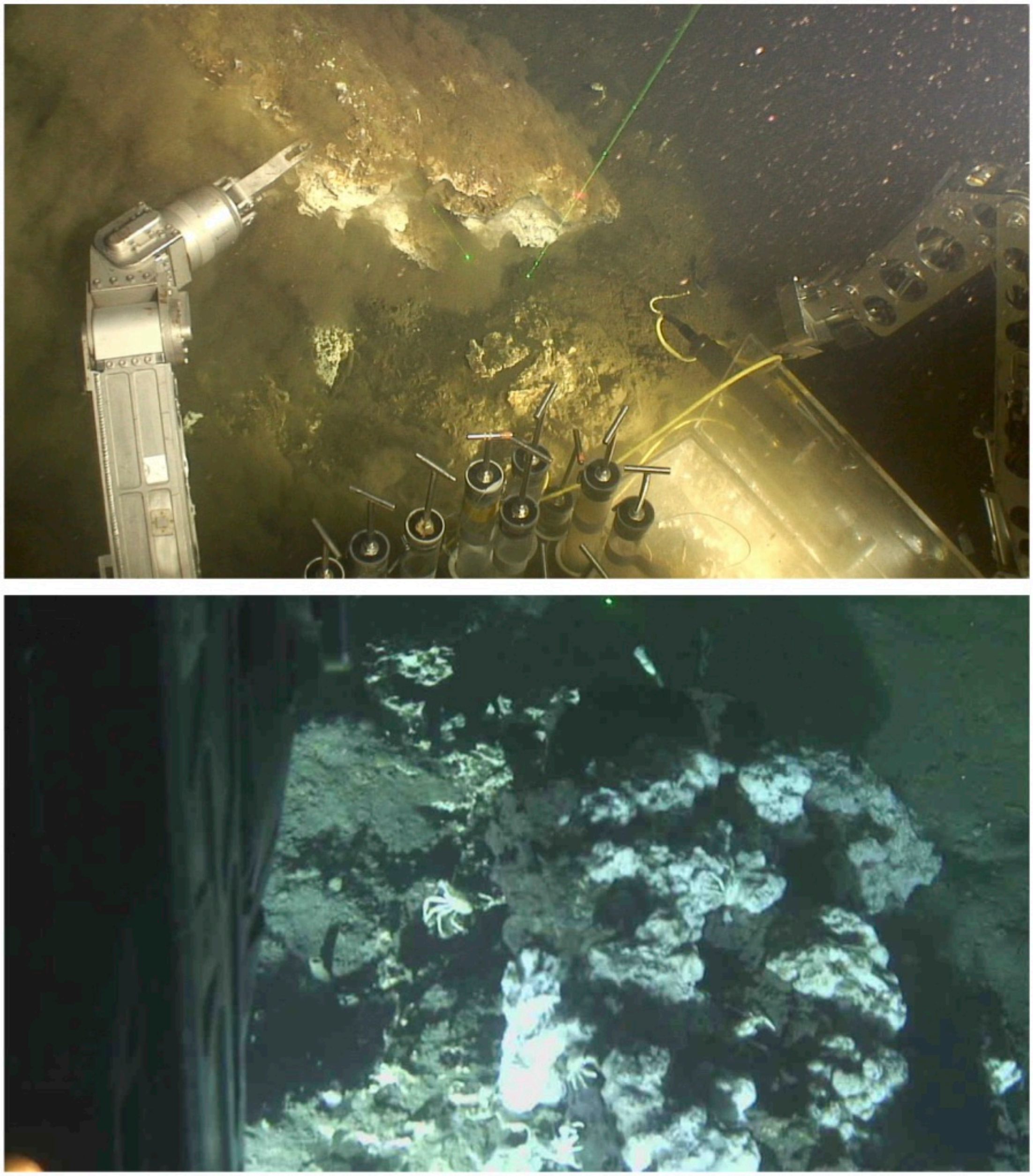

Supplementary Figure 6. Sampling sites for mineral specimens shown in Figure 3. Top, *Alvin* is sampling a large, lightly sediment-covered silicate without visible epifauna at ORP site, Dive 4864. Bottom, pre-sampling view of a conspicuous, craggy outcrop yielding silicates with aragonite veins, with sponge-like white and grey overgrowth and galatheid crabs, rising ca 0.5 m above the surrounding sediment at Mound 1 site, Dive 4864.

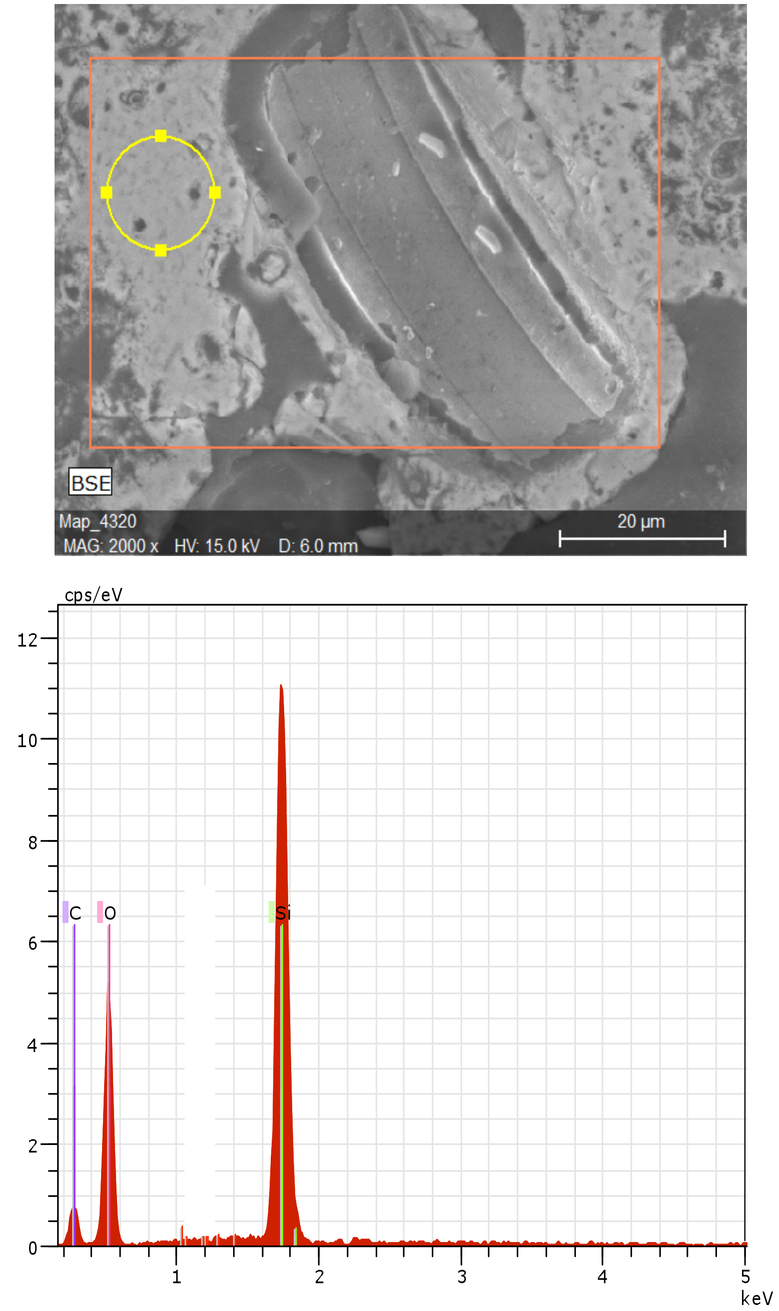

Supplementary Figure 7. Scanning electron microscopy and energy-dispersive X-ray Spectroscopy (SEM-EDS) of seafloor silicate samples from Ringvent, collected at the ORP site during *Alvin* dive 4864. Top, SEM microphotograph of thin section surface, with centric diatom embedded in the silica matrix. Bottom, EDS spectrum of the mineral surface in the area marked by the yellow circle, with dominant Si peak and smaller C and O peaks.

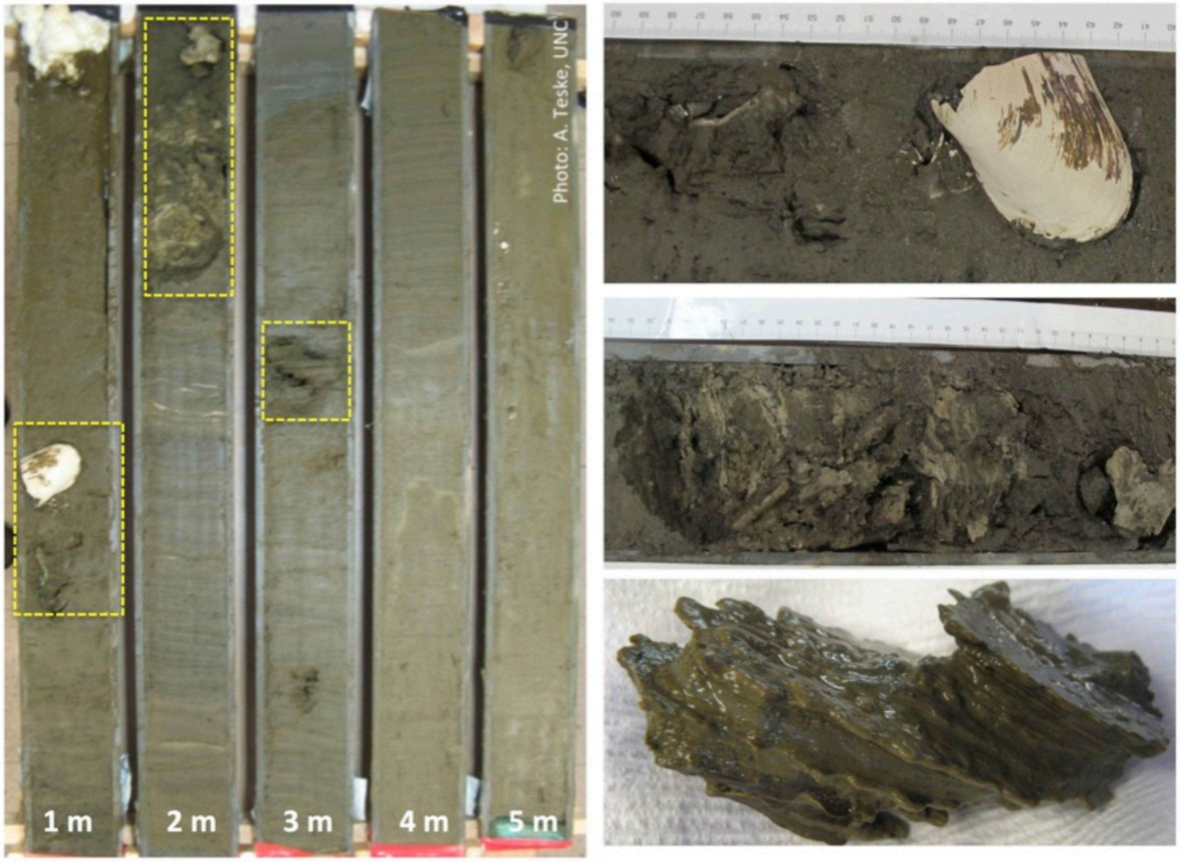

Supplementary Figure 8. Piston core from the central Ringvent area. Core P11 was collected during the RV *El Puma* survey in October 2014 at 27°N30’5090/111°W40’6860 in the central bowl of Ringvent. The core penetrated nearly 5 m into the ochre-brown diatom-ooze sediment (left panel), and recovered a seep clam (probably *Archivesica gigas*) at ca. 0.5 mbsf (top right), carbonate nodules that started at ca. 0.5 m depth and were most abundant at 1.0 to 1.25 mbsf (middle right), and hydrothermally altered and recrystallized silica, including a conspicuous silica nodule at 2.35 mbsf (bottom right).

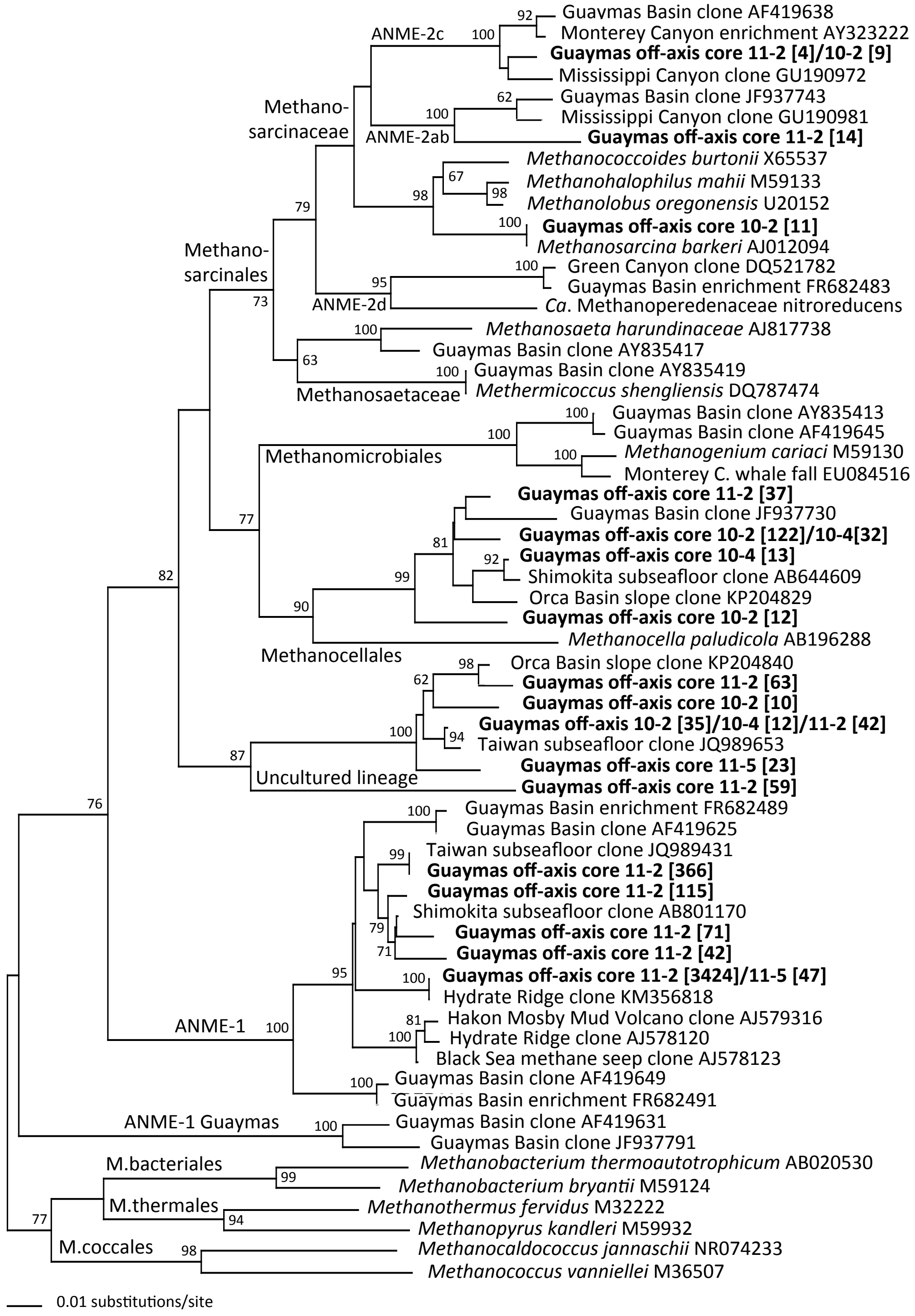

Supplementary Figure 9. Distance tree of representative archaeal partial 16S rRNA gene sequences (*E. coli* positions 517–958) from the Guaymas Basin v4/v5 amplicon dataset, annotated with the number of sequences within each OTU [in brackets] separately for each sample in cores P10 and P11.

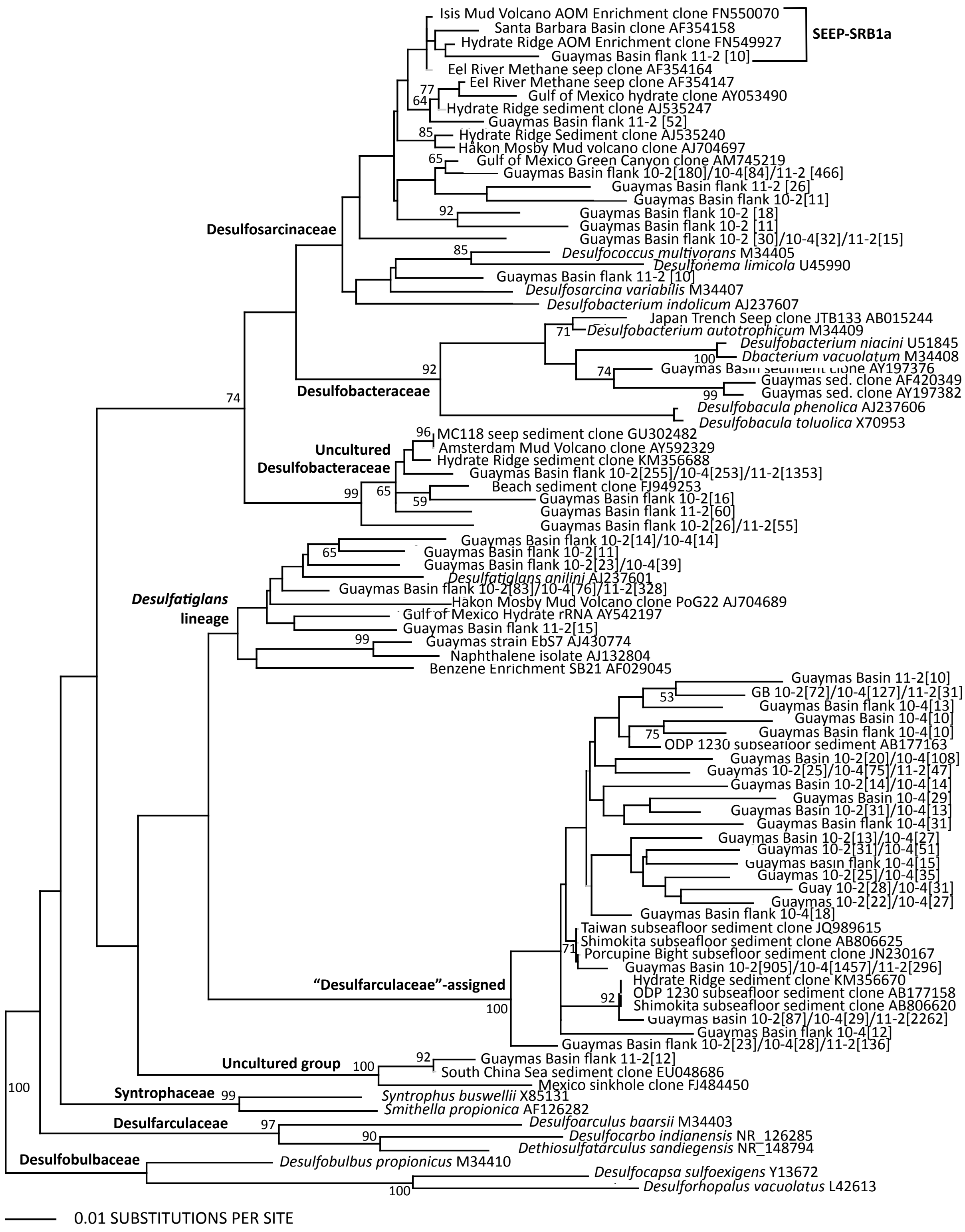

Supplementary Figure 10. Distance tree of representative deltaproteobacterial partial 16S rRNA gene sequences (*E. coli* positions 517–958) from the Guaymas Basin v4/v5 amplicon dataset, annotated with the number of sequences within each OTU [in brackets] separately for each sample in cores P10 and P11.

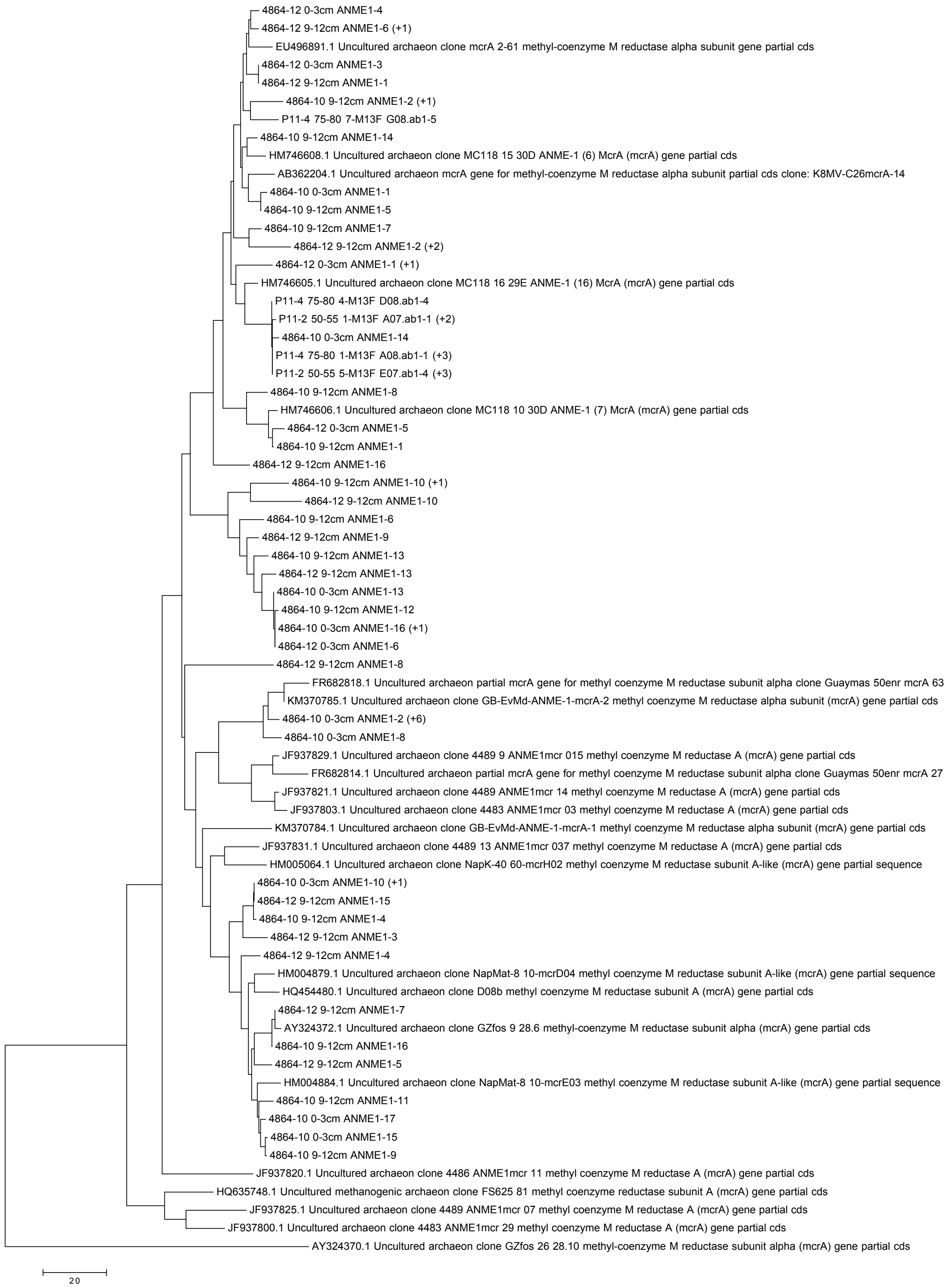

Supplementary Figure 11. Distance tree of the methyl-coenzyme M reductase alpha subunit gene, inferred with MEGA. The Ringvent sequences start with dive code 4864, followed by core number 10 or 12, and cm layer below surface. Multiple near-identical sequences representing the same phylogenetic branch are indicated in parentheses.

Supplemental Tables

Supplementary Data 1. Porewater sulfide concentrations in Ringvent sediments, measured in *Alvin* pushcores sampled during *Alvin* dive 4864 at the Mound 1 site (Supplementary Figure 2).

Sediment depth	4861-10	4864-12
0-3 cm	267 µM	8 µM
3-6 cm	No data	no data
6-9 cm	955 µM	No data
9-12 cm	No data	No data
12-15 cm	796 µM	285 µM
15-18 cm	625 µM	No data

Supplementary Data 2. XRD analysis of mineral concretions from core P11, performed with Rigaku Smart Lab XRD using 0.003º resolution, 1º/minute using a ICDD PDF4+ 2019 database (Ivano Aiello, Moss Landing Marine Labs).

Sample	Sediment depth in cm	Sample type	Composition
P11_1_42-48	42-48	Clam shell	100% aragonite
P11_1_54-60 cm	54-60	Carbonate concretion	~98% magnesian calcite, ~2% quartz
P11_2_4-9 cm	94-99	Carbonate concretion	~77% magnesian calcite, ~8% halite, ~2% pyrite, ~6% quartz, ~3% calcite; ~4% unknown
P11_2_12-15 cm	102-105	Carbonate concretion	~77% magnesian calcite, ~8% halite, ~2% pyrite, ~6% quartz, ~3% calcite; ~4% unknown
P11_2_24-31 cm	114-121	Carbonate concretion	~77% magnesian calcite, ~8% halite, ~2% pyrite, ~6% quartz, ~3% calcite; ~4% unknown
P11_3_10-11 cm	200-201	Ash	~50%quartz, ~27% orthoclase, ~17% muscovite, ~4%pyrite, ~2% anorthite
P11_3_38-39 cm	228-229	Ash	~36%albite, ~30%halite, ~25%quartz, ~5%pyrite; includes opal-A
P11_3_40-45 cm	230-235	Large 'laminated' silica concretion with microbial beads	Mainly opal-A with ~8% halite, 6% quartz
P11_3_78-79	268-269	Ash	~24% albite, ~22% halite, ~22% gaylussite, ~17% quartz, ~12% calcite, ~5% pyrite; includes opal-A

Supplementary Data 3. Isotopic data for carbonate nodules and sedimentary carbonates. Data are listed by sample identification in the left column [core No., section number, cm within section, sample type], followed by total sediment depth in cm, sample type, and $\delta^{13}C$ and $\delta^{18}O$ isotopic composition relative to VPDB, with standard deviations. Surficial minerals with fibrous aragonite veins, and the clam shell collected at the Mound 1 site of Ringvent are listed after the piston core samples. Sediments immediately adjacent to and surrounding a nodule are contrasted to sediments without direct association to nodules, matching the color and symbol code in Figure 4.

Core_section_cm	cm in core	sample type	$\delta^{13}C$ [‰]	± 1 s.d.	$\delta^{18}O$ [‰]	± 1 s.d.
P11_1_28-29	28.5	bulk sediment	0.24	0.04	-0.40	0.06
P11_1_42-48	45	Clam shell	0.52	0.02	3.71	0.05
P11_1_54-60	57	Non-skeletal nodule	-33.97	0.03	4.24	0.06
P11_1_54-60	57	sediment adjacent to nodule	-14.83	0.02	1.72	0.03
P11_1_73-74	73.5	bulk sediment	-2.89	0.03	0.15	0.05
P11_1_78-79	78.5	bulk sediment	-8.48	0.04	0.49	0.08
P11_1_83-86	84.5	Non-skeletal nodule	-37.99	0.03	3.64	0.02
P11_1_83-86	84.5	sediment adjacent to nodule	-30.5	0.02	2.62	0.04
P11_2_03-04	93.5	bulk sediment	-9.67	0.06	0.38	0.07
P11_2_04-09	96.5	Non-skeletal nodule	-38.34	0.02	3.61	0.04
P11_2_12-15	103.5	Non-skeletal nodule	-39.54	0.03	3.65	0.02
P11_2_12-15	103.5	sediment adjacent to nodule	-30.9	0.01	2.75	0.04
P11_2_15-18	106.5	Non-skeletal nodule	-36.81	0.04	3.81	0.06
P11_2_15-18	106.5	sediment adjacent to nodule	-33.38	0.01	2.97	0.02
P11_2_18-21	109.5	Non-skeletal nodule	-37.14	0.03	3.68	0.01
P11_2_18-21	109.5	sediment adjacent to nodule	-36.5	0.02	3.25	0.04
P11_2_21-24	112.5	bulk sediment	-39.81	0.04	3.78	0.03
P11_2_24-31	117.5	Non-skeletal nodule	-39.96	0.02	3.80	0.03

P11_2_34-35	124.5	bulk sediment	-3.23	0.03	0.19	0.06
P11_3_53-54	243.5	bulk sediment	-0.73	0.02	-0.09	0.08

P10_1_01-02	1.5	bulk sediment	n.d.	n.d.	n.d.	n.d.
P10_1_26-27	26.5	bulk sediment	n.d.	n.d.	n.d.	n.d.
P10_1_51-52	51.5	bulk sediment	0.25	0.03	-0.65	0.06
P10_1_77-78	77.5	bulk sediment	n.d.	n.d.	n.d.	n.d.
P10_2_01-02	100.5	bulk sediment	0.51	0.03	-0.25	0.07
P10_2_26-27	125.5	bulk sediment	n.d.	n.d.	n.d.	n.d.
P10_2_51-52	150.5	bulk sediment	0.15	0.03	-0.03	0.07
P10_2_77-78	176.5	bulk sediment	n.d.	n.d.	n.d.	n.d.
P10_3_01-02	200.5	bulk sediment	0.28	0.03	-0.24	0.04
P10_3_26-27	225.5	bulk sediment	0.1	0.03	0.45	0.03
P10_3_51-52	250.5	bulk sediment	0.04	0.04	-0.45	0.07
P10_3_77-78	276.5	bulk sediment	n.d.	n.d.	n.d.	n.d.
P10_4_01-02	299.5	bulk sediment	0.12	0.02	-0.32	0.06
P10_4_26-27	324.5	bulk sediment	0.25	0.02	-0.12	0.05
P10_4_51-52	349.5	bulk sediment	n.d.	n.d.	n.d.	n.d.
P10_4_77-78	375.5	bulk sediment	0.37	0.03	-0.07	0.08

P05_1_00-05	2.5	bulk sediment	-0.66	0.05	-1.07	0.07
P05_1_25-30	27.5	bulk sediment	n.d.	n.d.	n.d.	n.d.
P06_1_50-55	52.5	bulk sediment	-1.26	0.04	-0.32	0.07
P06_2_00-05	103	bulk sediment	n.d.	n.d.	n.d.	n.d.
P06_3_00-05	203	bulk sediment	-1.12	0.03	0.05	0.05
P06_3_50-55	253	bulk sediment	-0.64	0.10	-0.01	0.05
P06_3_75-80	278	bulk sediment	-1.18	0.01	0.66	0.02
P06_4_00-05	303	bulk sediment	n.d.	n.d.	n.d.	n.d.
P06_4_25-30	328	bulk sediment	-1.17	0.03	0.43	0.04
P06_4_50-55	353	bulk sediment	-1.03	0.03	0.00	0.08

P12_1_34-36	35	bulk sediment	9.52	2.56	0.99	0.06
P12_2_85-86	185	bulk sediment	7.44	2.42	1.46	0.18

Mound 1, surface	Fibrous Aragonite	-45.1	n.a.	3.2	n.a.
Mound 1, surface	Fibrous Aragonite	-47.2	n.a.	4.1	n.a.
Mound 1, surface	Clam shell	1.1	n.a.	3.4	n.a.

Supplementary Data 4. Radiocarbon [^{14}C] ages and sedimentation rates for Guaymas Basin and Sonora Margin sediments. Sedimentation rates were not inferred from cores P5 and P6 due to distortions by slumping. Measurements were made on bulk sediment without authigenic carbonate phases, and thus the majority of carbon in the samples is organic carbon produced by primary producers. Sedimentation Rates are based on using the youngest and oldest samples to obtain an average for the dated part of the core.

Core	Depth in core [cm]	^{14}C age [ybp]	Calendar age [ybp]	Sed. Rate [mm/year]
P03	274	6895 ± 35	6999	0.39
P10	1.5	1375 ± 30	567	
	350	6730 ± 35	6791	0.56
P11	3.5	1595 ± 25	758.5	
	47.5	3690 ± 35	3289	
	73.5	7075 ± 30	7183	
	244	10635 ± 40	11258	0.2286
P12	5.5	2200 ± 35	1367	
	35	2500 ± 30	1673	
	150	3585 ± 30	2986	
	185	3875 ± 30	3339	
	274	4330 ± 30	3905	
	321	4665 ± 30	4351	
	361	4650 ± 35	4335	0.97
P13	3.5	1620 ± 30	775	
	30	5405 ± 30*	5371*	
	93	2680 ± 35	227	
	156	3170 ± 30	400	
	226	3400 ± 30	693	
	311	4315 ± 30	1769	0.99

* outlier

Supplementary Data 5. Concentration and isotopic data for gravity core porewater chemistry. Data for each core are listed [from left to right] by sample number, actual sediment depth in cm, concentration in millimolar, $\delta^{13}C$ isotopic composition in per mil, and standard deviations of isotopic composition when available for multiple measurements. A depth of -2 cm indicates supernatant samples, collected from the top of freshly recovered piston cores.

A) Porewater methane

**CORE P6**	cm	mM	$\delta^{13}C$ [‰]	± 1 sd
1	2	0.09	-79.85	No data
2	52	0.27	-80.23	No data
3	102	0.81	-85.4	No data
4	152	6.81	-87.2	0.041
5	202	4.72	-86.91	0.215
6	252	10.73	-86.43	0.082
7	302	10.68	-86.2	0.008
8	352	9.51	-86.16	0.157
9	392	7.75	-86.42	0.479

**CORE P10**	cm	mM	$\delta^{13}C$ [‰]	± 1 sd
1	-2	0.006	-63.87	No data
2	2	0.0077	-64.77	No data
3	52	0.0091	-68.12	No data
4	101	0.0103	-68.24	No data
5	151	0.0077	-70.79	No data
6	201	0.0051	-68.5	No data
7	251	0.007	-67.55	No data
8	300	0.0137	-66.63	No data
9	350	0.0064	-69.09	No data
10	392	0.006	-67.66	No data

**Core P11**	cm	mM	$\delta^{13}C$ [‰]	± 1 sd
1	2	0.0107	-66.12	No data
2	52	0.1809	-62.62	No data
3	92	0.4873	-62.39	No data
4	142	1.01	-62.54	0.501
5	192	1.7069	-61.14	No data
6	242	1.41	-60.91	0.095
7	291.5	1.47	-60.43	0.052
8	341.5	1.24	-59.96	0.146

9	390.5	1.24	-60.06	0.351
10	440.5	1.37	-59.9	0.204
11	478.5	1.34	-60.47	0.474

**Core P12**	cm	mM	$\delta^{13}C$ [‰]	± 1 sd
1	-2	0	n.a.	No data
2	2	0.05	-75.79	No data
3	52	0.13	-75.62	No data
4	101.5	0.15	-77.78	No data
5	151.5	0.27	-81.12	No data
6	200.5	0.99	-88.81	0.356
7	250.5	4.3	-89.28	0.008
8	300.5	6.35	-88.11	0.161
9	350.5	8.03	-87.91	0.383
10	392.5	8.09	-87.97	0.214

B) Porewater DIC

**CORE P6**	cm	mM	$\delta^{13}C$ [‰]	± 1 sd
1	5	14.87	-14.54	0.141
2	55	30.47	-16.01	0.201
3	105	37.96	-16.37	0.122
4	155	49.04	-12.72	0.165
5	205	44.12	-11.29	0.076
6	255	49.42	-10.79	0.001
7	305	43.87	-11.03	0.223
8	355	46.41	-11.2	0.112
9	395	41.66	-10.98	0.057

**CORE P10**	cm	mM	$\delta^{13}C$ [‰]	± 1 sd
1	5	3.48	-6.54	0.274
2	55	4.17	-8.37	0.086
3	104	4.66	-8.8	0.146
4	154	4.23	-9.29	0.115
5	204	3.47	-9.61	0.053
6	254	4.86	-9.74	0.035
7	303	6.09	-9.69	0.107
8	353	2.95	-10.67	0.112
9	403	4.04	-9.9	0.045

**Core P11**	cm	mM	$\delta^{13}C$ [‰]	± 1 sd
1	-2	1.75	-0.35	0.385

2	5	2.65	-2.62	0.056
3	55	2.63	-2.73	0.062
4	80	3.38	-5.23	0.119
5	95	3.5	-4.64	0.181
6	145	3	-4.46	0.023
7	195	2.99	-4.65	0.039
8	240	3.43	-5.07	0.135
9	294.5	3.71	-5.65	0.037
10	344.5	4.38	-8.12	0.395
11	443.5	4.14	-8.6	0.184
12	468.5	3.9	-8.46	0.048

**Core P12**	cm	mM	$\delta^{13}C$ [‰]	± 1 sd
1	-2	1.41	-2.89	0.22
2	5	11.09	-15.38	0.238
3	55	23.22	-18.09	0.043
4	104.5	18.65	-17.81	0.005
5	154.5	31.92	-19.12	0.012
6	203.5	32.53	-18.7	0.020
7	253.5	32.98	-16.96	0.093
8	303.5	17.26	-13.64	0.042
9	353.5	25.44	-13.66	0.019
10	404.5	19.63	-15.24	0.037

C) Sulfate

Data for each core are listed [from left to right] by core segment, actual sediment depth in cm, concentration in millimolar as determined by ion chromatography, concentration in millimolar as determined by gravimetry, and $\delta^{34}S$ isotopic composition of barium-precipitated sulfate in per mil relative to VCDT. $\delta^{34}S$ data in red are based on very small sulfate amounts.

**Core P6**	cm	mM	mM	$\delta^{34}S-SO_4^{2-}$
1_0_5	0-5	19.52	17.99	39.5
1_25_30	25-30	12.37	11.69	54.7
1_50_55	50-55	7.33	8.43	65.5
1_75_80	75-80	No data	4.77	76.0
2_0_5	100-105	1.94	1.54	77.5
2_25_30	125-130	No data	0.07	35.9

2_50_55	175-180	No data	0.04	23.6
2_75_80	200-205	0.06	0.06	37.6
3_0_5	250-255	0.51	No data	No data
3_25_30	275-280	0.52	0.04	21.2
3_50_55	300-305	0.48	No data	No data
3_75_80	325-330	0.19	0.03	27.6
4_0_5	350-355	0.59	0.04	28.4
4_25_30	375-380	0.19	No data	No data
Core catcher	401	No data	0.06	25.9

**Core P10**	cm	mM	mM	$\delta^{34}S\text{-}SO_4^{2-}$
supernatant	0	29.20	25.88	21.2
1_0_5	0-5	28.03	26.24	23.2
1_25_30	25-30	27.66	26.13	24.3
1_50_55	50-55	27.66	24.52	24.6
1_75_80	75-80	27.45	25.68	24.8
2_0_5	99-104	27.36	25.17	24.9
2_25_30	124-129	27.24	24.87	24.9
2_50_55	149-154	27.25	25.71	25.1
2_75_80	174-179	27.31	26.96	25.1
3_0_5	199-204	27.36	22.64	25.1
3_25_30	224-229	28.01	25.85	25.3
3_50_55	249-254	27.42	26.54	25.2
3_75_80	274-279	27.39	24.77	25.3
4_0_5	298-303	27.40	25.52	25.2
4_25_30	323-328	27.40	25.57	25.4
4_50_55	348-353	27.23	26.49	25.4
4_75_80	373-378	27.82	25.51	25.4
Core catcher	393-403	27.43	26.23	25.4

**Core P11**	cm	mM	mM	$\delta^{34}S\text{-}SO_4^{2-}$
supernatant	0	29.56	24.86	21.3
1_0_5	0-5	28.90	27.10	21.5
1_25_30	25-30	28.87	27.71	21.4
1_50_55	50-55	29.26	26.56	21.7
1_75_80	75-80	28.99	26.99	21.9
1_0_5	90-95	No data	27.73	21.8
2_25_30	115-120	28.93	27.42	21.9
2_50_55	140-145	28.81	26.96	22.1
2_75_80	165-170	28.80	26.06	22.1
3_0_5	190-195	28.74	26.95	22.1

3_25_30	215-220	28.80	29.59	22.2
3_45_50	240-245	28.78	27.33	22.3
3_75_80	265-270	28.89	26.54	22.4
4_0_5	289.5-294.5	28.68	28.19	22.4
4_25_30	314.5-319.5	29.23	28.43	22.6
4_50_55	339-5-344.5	28.70	28.69	23.1
4_75_80	364.5-369.5	28.67	26.07	23.0
5_0_5	388.5-393.5	28.34	28.90	23.1
5_25_30	413.5-418.5	28.02	26.92	23.0
5_50_55	438.5-443.5	28.19	28.03	23.1
5_75_80	463.5-468.5	28.31	28.74	23.2

**Core P12**	cm	mM	mM	$\delta^{34}S\text{-}SO_4^{2-}$
Supernatant	0	28.95	29.13	20.9
1_0_5	0-5	20.03	20.03	37.2
1_25_30	25-30	13.88	14.11	51.6
1_50_55	50-55	11.35	11.13	55.2
1_75_80	75-80	7.91	9.03	62.0
2_0_5	99.5-104.5	6.12	0.07	16.8
2_25_30	124.5-129.5	4.33	4.36	70.7
2_50_55	149.5-154.5	0.41	2.70	78.4
2_75_80	174.5-179.5	1.68	1.99	83.9
3_0_5	198.5-203.5	0.74	0.09	44.0
3_25_30	223.5-228.5	0.71	0.03	24.6
3_50_55	248.5-253.5	2.54	0.08	28.0
3_75_80	273.5-278.5	0.69	No data	No data
4_0_5	298.5-303.5	No data	0.15	20.1
4_25_30	323.5-328.5	0.79	0.03	29.2
4_50_55	348.5-353.5	0.67	0.02	30.1
4_75_80	373.5-378.5	0.54	No data	No data
Core catcher	394.5-404.5	0.37	0.02	18.8

D) Sulfide

Data for each core are listed [from left to right] by sample number, actual sediment depth in cm, and concentration in micromolar.

**CORE P6**	cm	µM
1	2.5	1310
2	27.5	2553
3	52.5	4161

4	77.5	3173
5	102.5	4151
6	127.5	2551
7	152.5	2818
8	177.5	5196
9	202.5	5453
10	227.5	5539
11	252.5	5853
12	277.5	3440
13	302.5	6196
14	327.5	5696
15	352.5	5810
16	377.5	3173
17	405.5	4796

**CORE P10**	cm	µM
1	-2	2.5
2	2.5	27.5
3	27.5	52.5
4	52.5	16.25
5	77.5	40
6	101.5	48.75
7	126.5	91.25
8	151.5	40
9	176.5	92.5
10	201.5	38.75
11	226.5	61.25
12	251.5	60
13	276.5	58.75
14	300.5	65
15	325.5	30
16	350.5	32.5
17	375.5	80
18	398	83.75

**Core P11**	cm	µM
1	-2	not detected
2	2.5	not detected
3	27.5	not detected
4	52.5	1.5

5	77.5	not detected
6	92.5	not detected
7	97.5	not detected
8	142.5	not detected
9	167.5	not detected
10	192.5	1.5
11	217.5	not detected
12	242.5	not detected
13	267.5	5.25
14	292	0.25
15	317	1.5
16	342	25.25
17	367	50.25
18	400	65.25
19	416	69
20	441	72.75
21	466	82.75

**Core P12**	cm	μM
1	-2	632
2	2.5	768
3	27.5	5428
4	52.5	6188
5	77.5	7848
6	102	3848
7	127	9548
8	152	9468
9	177	10588
10	201	10248
11	226	10308
12	251	9148
13	276	8708
14	301	1988
15	326	8008
16	351	8728
17	376	8748
18	399.5	7048

Supplementary Data 6. Numbers and relative proportions (%) for sequences of methanogenic, methane-oxidizing and sulfate-reducing microbial lineages in the v4/v5 Miseq 16S rRNA gene dataset obtained from Guaymas Basin piston-cored sediments, based on the taxonomy pipeline SILVA v. 119 in VAMPS (*Huse et al.* 2014a) and on reanalysis with MOTHUR and SILVA v. 132 (see methods). "Desulfarculaceae" indicates a heterogeneous and variable pipeline classification that is not supported by phylogenetic analysis of representative sequences using PAUP (*Swofford* 2000); members of the *Desulfatiglans* lineage are also subsumed under this designation.

**MOTHUR (SILVA 132)**	**P03_4**	**P04_3**	**P06_3**	**P06_4**	**P10_2**	**P10_4**	**P11_2**	**P11_5**	**P12_4**	**P13_4**
Methanocellales	146 (0.14)	54 (0.06)	103 (0.11)	60 (0.06)	156 (0.16)	47 (0.05)	39 (0.04)	0 (0)	19 (0.02)	46 (0.03)
Methanosarcinaceae	29 (0.03)	51 (0.06)	37 (0.03)	95 (0.10)	18 (0.02)	0 (0)	0 (0)	0 (0)	3 (<0.01)	58 (0.04)
Methermicoccaceae	9 (<0.01)	1 (<0.01)	0 (0)	0 (0)	29 (0.10)	9 (0.01)	6 (<0.01)	0 (0)	0 (0)	7 (<0.01)
Methanosarcinales [ANME -2a,b,c]	0 (0)	0 (0)	0 (0)	2 (<0.01)	9 (<0.01)	0 (0)	20 (0.02)	0 (0)	0 (0)	27 (0.02)
Methanomicrobia [ANME-1]	5 (<0.01)	7 (<0.01)	11971 (12.33)	16646 (17.15)	10 (0.01)	0 (0)	4215 (4.00)	35 (0.05)	4607 (5.12)	15 (0.01)
"Desulfarculaceae" incl. Desulfatiglans	3320 (2.38)	No data	1183 (0.86)	1147 (0.96)	1881 (2.27)	3566 (2.72)	3438 (2.33)	No data	1351 (1.00)	3112 (2.11)
Desulfobacteraceae	530 (0.40)	No data	638 (0.47)	588 (0.50)	570 (0.69)	381 (0.29)	2214 (1.50)	No data	76 (0.06)	1133 (0.79)
**VAMPS (SILVA 119)**										
Methanocellales	101 (0.14)	41 (0.06)	51 (0.06)	40 (0.05)	104 (0.15)	33 (0.05)	36 (0.05)	0 (0)	12 (0.02)	26 (0.03)
Methanosarcinaceae	18).02)	30 (0.05)	16 (0.02)	42 (0.05)	12 (0.02)	0 (0)	0 (0)	0 (0)	2 (<0.01)	32 (0.04)
Methermicoccaceae	71 (0.10)	45 (0.07)	0 (0)	1 (<0.01)	70 (0.10)	45 (0.07)	103 (0.13)	21 (0.04)	17 (0.02)	6 (<0.01)
Methanosarcinales [ANME-2a,b,c]	0 (0)	0 (0)	0 (0)	2 (<0.01)	7 (0.01)	0 (0)	19 (0.02)	0 (0)	0 (0)	19 (0.02)
Methanomicrobia [ANME-1]	14 (0.02)	14 (0.02)	9798 (12.15)	13592 (16.84)	11 (0.01)	0 (0)	3567 (4.65)	44 (0.07)	4591 (6.13)	11 (0.01)
"Desulfarculaceae" incl. Desulfatiglans	1461 (1.39)	No data	811 (0.72)	762 (0.78)	791 (1.25)	1613 (1.64)	2534 (2.16)	No data	641 (0.60)	1353 (1.23)
Desulfobacteraceae	578 (0.55)	No data	646 (0.58)	583 (0.59)	542 (0.86)	388 (0.39)	1941 (1.66)	No data	153 (0.14)	1089 (0.99)

Supplementary Data 7. Listing of representative partial 16S rRNA gene sequences used for inferring ANME and sulfate reducer phylogenies in Supplementary figures 9 and 10, in order of appearance from the top to the base of each tree. The sequence number [denovoXXXX] is followed by sediment sample [P11_2 and P11_5; P10_2 and P10_4], and the number of sequences within the same OTU [CT = counts], and phylogenetic affiliation. If an OTU occurs in multiple samples, these samples and their corresponding sequence counts are listed separately.

ANME/Methanogen phylogeny, Suppl. Figure 9

>denovo3767 P11_2 CT14 [**ANME-2ab**]

GTCTAAAGGGTCTGTAGCCTGTTTAATAAGTTCTTTGGGAAATCTGGCAGCTTAACTGTCAGGCTGCTAAAGGATAC
TGTTAAACTTGGGACCGGGGGACGTAGGGGGTACTCCAGGGGTAGGAGTGAAATCTTGTAATCCCTGGGGGACCA
TCTGTGGCGAAGGCGCCTTACGAGAACGGGTCCGACGGTGAGGGACGAAAGCTAGGGGAGCAAACCGGATTAGA
TACCCGGGTAGTCCTAGCTGTAAACGATGCTCGCTAGGTGTCCGGCACGGTGCGTCCGTGTCTGGTGCCGCAGGGA
AGCCGTTAAGCGAGCCACCTGGGAAGTACGGTCGCAAGGCTGAAACTTAAAGGAATTGGCGGGGGGGCACTACAA
CGGGTGGAGCCTGCGGTGTAATTGGACTCAACGCCGG

>denovo2035 P10_2 CT09 / P11_2 CT4 [**ANME-2c**]

GTCTAAAGCGTTCGTAGCCGGTTTGGTAAGTCCTCTGGAAAATCTGGTTGCTCAACAATCAGACTGCCAAGGGATAC
TGTCGAACTTGAGACCGGGAGAGGTAAGAGGTACTTCAGGGGTAGGAGTGAAATCTTGTAATCCCTGGGGGACCA
TCTGTGGCGAAGGCGTCTTACCAGAACGGGTCTGACGGTGAGGGACGAAAGCTGGGGGCGCGAACCGGATTAGAT
ACCCGGGTAGTCCCAGCCGTAAACGATGCTCGCTATGTGTCAGGTACGGTGCGACCGTATCTGGTGCCGTAGGGAA
GCCGTGAAGCGAGCCACCTGGGAAGTACGGCCGCAAGGCTGAAACTTAAAGGAATTGGCGGGGGAGCACTACAAC
GGGTGGAGCCTGCGGTTTAATTGGAATCAACGCCGG

>denovo13073 P10_2 CT11 [**genus *Methanosarcina*]**

GTCTAAAGGGTCTGTAGCCGGTTTGGTCAGTCCTCCGGGAAATCTGATAGCTCAACTATTAGGCTTTCGGGGGATAC
TGCCAGACTTGGAACCGGGAGAGGTAAGAGGTACTACAGGGGTAGGAGTGAAATCTTGTAATCCCTGTGGGACCA
CCTGTGGCGAAGGCGTCTTACCAGAACGGGTTCGACGGTGAGGGACGAAAGCTGGGGGCACGAACCGGATTAGAT
ACCCGGGTAGTCCCAGCCGTAAACGATGCTCGCTAGGTGTCAGGCATGGCGCGACCGTGTCTGGTGCCGCAGGGAA
GCCGTGAAGCGAGCCACCTGGGAAGTACGGCCGCAAGGCTGAAACTTAAAGGAATTGGCGGGGGAGCACAACAA
CGGGTGGAGCCTGCGGTTTAATTGGATTCAACGCCGG

>denovo13373 P11_2 CT37 [**Methanocellales**]

GTCTAAAGTGTCTGTAGCCGGTTCGGCAAGTCCCTTGGGAAATCTGACGGCTTAACCGTTAGGCGTCCAGGGGATA
CTACCGAACTTGGGACCGGGAGAGGTAGGAGGTACTCCGGGGGTAGGGGTGAAATCCTGTAATCCTTGGGGGACC
ACCGGTGGCGAAGGCGTCCTACCAGAACGGCTCCGACGGTGAGGGACGAAAGCTAGGGGCACGAACCGGATTAG
ATACCCGGGTAGTCCTAGCCGTAAACGATGCGGGCTAGGTGTCACAGTAGCCATGTGTTGCTGTGGTGCCGCAGGG
AAACCGTGAAGCCTGCCGCTTGGGAAGTACGTCCGCAAGGATGAAACTTAAAGGAATTGGCGGGGGAGCACTACA
ACGGGTGGAGCCTGCGGTTTAATTGGACTCAACGCCGG

>denovo3955 BSKE9 P10_2 CT122 / P10_4 CT32 [**Methanocellales**]

GCCTAAAGCATCCGTAGCCGGCTCGGCAAGTCTCTTGGGAAATCTGACGGCTTAACCGTCGGGCGTCCAGGGGATA
CTACCGGGCTTGGGACCGGGAGAGGTAGGAGGTACTCCGGGGGTAGGGGTGAAATCCTGTAATCCTCGGGGGACC
ACCGGTGGCGAAGGCGTCCTACCAGAACGGCTCCGACGGTGAGGGACGAAAGCTAGGGGCACGAACCGGATTAG
ATACCCGGGTAGTCCTAGCCGTAAACGATGCGGGCTAGGTGTCACGGTAGCCATGTGCTGCCGTGGTGCCGCAGGG
AAACCGTGAAGCCTGCCGCTTGGGAAGTACGTCCGCAAGGATGAAACTTAAAGGAATTGGCGGGGGAGCACTACA
ACGGGTGGAGCCTGCGGTTTAATTGGACTCAACGCCGG

>denovo12316 P10_4 CT13 [**Methanocellales**]

GTCTAAAGGGTCTGTAGCCGGCCCGGAAAGTCCCTTGGGAAATCTGTCGGCTTAACCGACAGGCGTTCAGGGGATA
CTACCGGGCTTGGGACCGGGAGAGGTAGGAGGTACTTCGGGGGTAGGGGTGAAATCCTGTAATCCCCGGGGGACC
ACCGGTGGCGAAGGCGTCCTACCAGAACGGCTCCGACGGTGAGGGACGAAAGCTAGGGGCACGAACCGGATTAG
ATACCCGGGTAGTCCTAGCCGTAAACGATGCGGGCTAGGTGTCACAGTAGCCATGTGCTGCTGTGGTGCCGAAGGG
AAACCGTGAAGCCTGCCGCTTGGGAAGTACGTCCGCAAGGATGAAACTTAAAGGAATTGGCGGGGGAGCACTACA
ACGGGTGGAGCCTGCGGTTTAATTGGATTCAACGCCGG

>denovo10768 P10_2 CT12 [**Methanocellales**]

GTCTAATTCGTAGCCGGCCTGGCCAGTCCCTTGGGAAATCTAGCGGCTTAACCGTTAGGCGTCCAGAGGATACTACC
AGGCTTGGGACCGGGAGAGGTAGGAGGTACCCCAGGGGTAGGGGTGAAATCCTGTAATCCTTGGGGGACCACCG
GTGGCGAAGGCGTCCTACCAGAACGGCTCCGACGGTGAGGGACGAAAGCTAGGGGCACGAACCGGATTAGATACC
CGGGTAGTCCTAGCCGTAAACGATGCAGGCTAGGTGTCACAGTAGCCATGAGCTATTGTGGTGCCGCAGGAAAACC
GTGAAGCCTGCCGCTTGGGAAGTACGTCCGCAAGGATGAAACTTAAAGGAATTGGCGGGGGAGCACTACAACGGG
TGGAGCCTGCGGTTTAATTGGAATCAACGCCGG

>denovo13609 Methermicoccus P11_2 CT63 [**uncultured lineage**]

GTCTAAAGGGTCTGTAGCCTGTCCAGCCAGTCCGTTGGGAAATCTGACAGCTTAACTGTCAGGCTGCTAAGGGATAC
TGTTGGACTTGGGACCGGGAGAGGCTAGAGGTACTTCAGGGGTAGGAGTGAAATCCTGTGATCCTTGAAGGACCA
CCGGTGGCGAAGGCGTCTAGCTAGAACGGCTCCGACGGTGAGGGACGAAAGCTAGGGGCACGAACGGGATTAGA
TACCCCGGTAGTCCTAGCCGTAAACGATGTGAGCTAGGTGTCAGCCTTTCTGCGAGAGAGGCTGGTGCCGTAGGGA
AGCCGTGAAGCTCACCACCTGGGAAGTACGGTCGCAAGGCTGAAACTATATGAAGTGGACTTCATGCGTTTGCTTCA
TTAGCAGCAAACTTAAAGGAATTGGCGGGGGAGCACTACAACGGGTGGATGCTGCGGTTTAATTGGAATCAACGCC
GG

>denovo12503 P10_2 CT10 [**uncultured lineage**]

GCTTAAAGCGTTCGTAGCCGGTCCAGCCAGTCCGTTGGGAAATCTGCCGGCTTAACCGTCAGGCTGCTCAGGTATAC
TGCTGGACTTGGGACCGGGAGAGGCTAGAGGTACTTCAGGGGTAGGAGTGAAATCCTGTGATCCTTGAAGGACCG
CCGGTGGCGAAGGCGTCTAGCTAGAACGGGTCCGACGGTGAGGGACGAAAGCTAGGGGCACGAACGGGATTAGA
TACCCCGGTAGTCCTAGCCGTAAACGATGTGAGCTAGGTGTCAGCCTTTCTGCGAGGGAGGCTGGTGCCGTAGGGA
AGCCGTGAAGCTCACCGCCTGGGAAGTACGGTCGCAAGGCTGAAACTTAAAGGAATTGGCGGGGGAGCACTACAA
CGGGTGGATGCCGCGGTTTAATTGGAATCAACGCCGG

>denovo13341 P10_2 CT35 / P10_4 CT12 / P11_2 CT42 [**uncultured lineage**]

GCCTAAAGCGTTCGTAGCCGGTCCAGCCAGTCCTTTGGGAAATCTGACGGCTTAACCGTCAGGCTGCTAAGGGATAC
TGCTGGACTTGGGACCGGGAGAGGCTAGAGGTATTTCAGGGGTAGGAGTGAAATCCTGTGATCCTTGAAGGACCA
CCGGTGGCGAAGGCGTCTAGCTAGAACGGCTCCGACGGTGAGGGACGAAAGCTAGGGGCACGAACGGGATTAGA
TACCCCGGTAGTCCTAGCCGTAAACGATGCGAGCTAGGTGTCAGCCTTTCTGCGAGAGAGGCTGGTGCCGTAGGGA

AGCCGTGAAGCTCGCCACCTGGGAAGTACGGTCGCAAGGCTGAAACTTAAAGGAATTGGCGGGGGAGCACTACAA
CGGGTGGATGCTGCGGTTTAATTGGACTCAACGCCGG

>denovo11053 P11_5 CT23 [**uncultured lineage**]

GTCTAAAGTGTCCGTAGCCTGTCCAGCCAGTCCTTTGTGAAATCTGACCGCTTAACTGTCAGGCTGCTAGGGGATAC
TGCTGGACTCGGGACCGGGAGAGGCTAGAGGTATTTCAGGGGTAGGAGTGAAATCCTGTAATCCTTGAAGGACCG
CCGGTGGCGAAGGCGTCTAGCTAGAACGGGTCCGACGGTGAGGGACGAAAGCTAGGGTCACGAACGGGATTAGA
TACCCCGGTAGTCCTAGCCGTAAACGATGCGAGCTAGGTGTCAGCCTTTCTGCGAGAGAGGCTGGTGCCGTAGGGA
AGCCGTGAAGCTCGCCACCTGGGAAGTACGGTCGCAAGGCTGAAACCACATGAAGTATACTTTATGAGCTTGCGTC
ATTGGCAGCAAACTTAAAGGAATTGGCGGGGGAGCACTACAACGGGTGGATGCTGCGGTTTAATTGGATTCAACGC
CGG

>denovo9206 P11_2 CT59 [**uncultured lineage**]

GTCTAAAGGGTCTGTAGCCGGCCTAGCAAGTCCTTTGGGAAATCTGGCAGCTTAACTGTCAGGATGCTAAAGGATA
CTACTGGGCTTGGGACCGGGTGAGGCTAGAGGTACCCCAGGGGTAGCGGTGAAATGCTGTAATCCTTGGGGGACT
ATCAGTGGCGAAGGCGTCTAGCCAGAACGGGTCCGACGGTGAGGGACGAAAGCTAGGGGCGCGAACGGGATTAG
ATACCCCGGTAGTCCTAGCCGTAAACGATGCGAGCTTTGTGTCGGCCTCGACGCGATCGGGGTCGGTGCCGTAGGG
AAGCTGTTAAGCTCGCCACCTGGGGAGTATGGTCGCAAGACTGAAACTTAAAGGAATTGGCGGGGGAGCACCACA
ACGGGTGGAGCCTGCGGTTCAATTGGAATCAACGCCGG

>denovo17179 P11_2 CT366 **[ANME-1 / ANME-1b]**

GTCTAAAGGGTCTGTAGCCGGCCAAGTAAGTTCTTTGGGAAATTTGACCGCTTAACGGTCAAGCTATCAGGGAATAC
TGCTTGGCTTGGGACCGGGAGAGGTCAGAGGTACTCCAAGGGTAGGGGTGAAATCCGTTAATCCTTGGGGGACCA
CCGGTAGCGAAGGCGTCTGACCAGACCGGGTCCGACGGTGAGGGACGAAGGCTAGGGTCGCGAACCGGATTAGA
TACCCGGGTAGTCCTAGCTGTAAACGATGCGGGCCAGGTGTTGGCATTACTGCGAGTGATGCCAGTGCCGAAGGGA
AGCCGTTAAGCCCGCCATCTGGGGAGTACGGTCGCAAGGCTGAAACTTAAAGGAATTGGCGGGGGAGCACCACAA
CGGGTGGAGCTTGCGGTTCAATTGGAATCAACGCCGG

>denovo6418 P11_2 CT115 **[ANME-1 / ANME-1b]**

GCTTAAAGTGTTCGTAGTCGGCCGGGTAAGTTCCTTGGGAAATTTGACCGCTTAACTGTCAAGCTTTCAGGGAATAC
TGCCTGGCTTGGGACCGGGAGAGGTCAGAGGTACTCCAAGGGTAGGGGTGAAATCCATTAATCCTTGGGGGACCA
CCGGTAGCGAAGGCGTCTGACCAGACCGGGTCCGACGGTGAGGGACGAAGGCTAGGGTCGCGAACCGGATTAGA
TACCCGGGTAGTCCTAGCTGTAAACGATGCGGGCCGGGTGTTGGCATTACTGCGAGTGATGCCAGTGCCGAAGGG
AAGCCGTTAAGCCCGCCATCTGGGGAGTACGGTCGCAAGGCTGAAACTTAAAGGAATTGGCGGGGGAGCACCACA
ACGGGTGGAGCTTGCGGTTCAATTGGACTCAACGCCGG

>denovo15530 P11_2 CT71 **[ANME-1 / ANME-1b]**

GTCTAACGCGTTCGTAGCCGGCCGGGTAAGTTCCTTGGGAAATTTGACCGCTTAACGGTCAAGCTTTCAGGGAATAC
TGCTTGGCTTGGGACCGGGAGAGGTCAGAGGTACTCCACGGGTAGGGGTGAAATCCATTAATCCTTGGGGGCCCA
CCGGTAGCGAAGGCGTCTGACCAGACCGGGTCCGACGGTGAGGGACGAAGGCTAGGGTCGCGAGCCGGATTAGA
TACCCGGGTAGTCCTAGCTGTAAACGATGCGGGCCAGGTGTTGGCATTACTGCGAGTGATGCCAGTGCCGAAGGGA
AGCCGTTAAGCCCGCCATCTGGGGAGTACGGTCGCAAGGCTGAAACTGAAAGGAATTGGCGGGGGAGCACCACAA
CGGGTGGAGCTTGCGGTTCAATTGGAATCAACGCCGG

>denovo4844 P11_2 CT42 **[ANME-1/ANME-1b]**

GCCTAAAGCATCCGTAGCCGGCCGGGTAAGTTCCTTGTGAAATTTGACCGCTTAACGGTCAAGCTTTCAGGGAATAC
TGCTTGGCTTGGGACCGGGAGAGGTCAGCGGTACTCCAAGGGTAGGGGTGAAATCCATTAATCCTTGGGGGACCA
CCGGTAGCGAAGGCGTCGGACCAGACCGGGTCCGACGGTGAGGGGCGAAGGCTAGGGTCGCGAACCGGATTAGA
TACCCTGGTAGTCCTAGCTGTAAACGATGCGGGCCAGGTGTTGGCATTACTGCGAGTGATGCCAGTGCCGAAGGGA
AGCCGTTAAGCCTGCCATCTGGGGAGTACGGTCGCAAGGCTGAAACTTAAAGGAATTGGCGGGGGAGCACCACAA
CGGGTGGAGCTTGCGGTTCAATTGGAATCAACGCCGG

>denovo9885 P11_2 CT3424 P11_5 CT47 [**ANME-1**]

GCCTAAAGCATCCGTAGCCGGCTGAGTAAGTTCCTTGGGAAATTTGACCGCTTAACGGTTAAGCTATCAGGGAATAC
TGCTTGGCTTGGGACCGGGAGAGGTCAGAGGTACTCCAGGGGTAGGGGTGAAATCTATTAATCCTTGGGGGACCA
CCGGTAGCGAAGGCGTCTGACCAGACCGGGTCCGACGGTGAGGGACGAAGGCTGGGGTCGCGAACCGGATTAGA
TACCCGGGTAGTCCCAGCTGTAAACGATGCGGGCCAGGTGTTGGCATTACTGCGAGTGATGTCAGTGCCAAAGGGA
AGCCGTTAAGCCCGCCATCTGGGGAGTACGGTCGCAAGGCTGAAACTTAAAGAAATTGGCGGGGGAGCACCACAA
CGGGTGGAGCCTGCGGTTCAATTGGACTCAACGCCGG

Sulfate-reducing deltaproteobacterial phylogeny, Suppl. Figure 10

>denovo7591 P11_2 CT10 **[Seep-SRB1a]**

CCAGCAGCTGCGGTAACACGGAGGGTGCAAGCGTTATTCGGAATTATTGGGCGTAAAGGGCGCGCAGGCGGTCTT
TTAAGTCAGCTGTGAAAGCCCGGGGCTCAACCCCGGAAGTGCATTTGAAACTAAGGGACTTGAGTATGGGAGAGG
GAAGTGGAATTCCTGGTGTAGAGGTGAAATTCGTAGATATCAGGAGGAACACCGGTGGCGAAGGCGACTTCCTGG
ACCAATACTGACGCTGAGGCGCGAAGGCGTGGGGAGCAAACAGGATTAGATACCCTGGTAGTCCACGCAGTAAAC
GGGGAACACTAGGTGTAGCGGGTATTGACCCCTGCTGTGCCGCAGTTAACGAATTAAGTGTTCCGCCTGGGGAGTA
CGACCGCAAGGTTAAAACTCAAAGGAAGTGACGG

>denovo2662 P11_2 CT52 **[Seep-SRB1]**

CCAGCAGCTGCGGTACTACGGAGGGTGCAAGCGTTATTCGGAATTATTGGGCGTAAAGGGCGCGTAGGCGGTCTTT
TAAGTCAGATGTGAAAGCCCGGGGCTTAACCCTGGAAGTGCATTTGAAACTGAAGGACTTGAGTATGGGAGAGGG
AAGTGGAATTCCTGGTGTAGAGGTGAAATTCGTAGATATCAGGAGGAACACCGGTGGCGAAGGCGACTTCCTGGA
CCAATACTGACGCTGAGGCGCGAAGGCGTGGGGAGCAAACAGGATTAGATACCCTGGTAGTCCACGCAGTAAACG
ATGAACACTAGGTGTAGCGGGTATTGACCCCTGCGGTGCCGCAGTTAACGCATTAAGTGTTCCGCCTGGGGAGTAC
GACCGCAAGGTTAAAACTCAAACGAATTGACGG

>denovo6456 P10_2 CT180 / P10_4 CT84 / P11_2 CT446 **[Desulfosarcinaceae]**

CCAGCAGCTGCGGTAACACGGGGGGTGCAAGCGTTATTCGGAATTATTGGGCGTAAAGGGCGCGTAGGCGGTCTC
TTAAGTCAGATGTGAAAGCCCGGGGCTTAACCCCGGAAGTGCATTTGAAACTAAGGGACTTGAGTATGGGAGAGG
GAAGTGGAATTCCTGGTGTAGAGGTGAAATTCGTAGATATCAGGAGGAACACCGGTGGCGAAGGCGACTTCCTGG
ACCAATACTGACGCTAAGGCGCGAAGGCGTGGGGAGCAAACAGGATTAGATACCCTGGTAGTCCACGCAGTAAAC
GGTGAACACTAGGTGTAGCGGGTATTGACCCCTGCTGTGCCGCAGCTAACGCATTAAGTGTTCCGCCTGGGGAGTA
CGGTCGCAAGATTAAAACTCAAACGAATTGACGG

>denovo8687 P11_2 CT26 **[Desulfosarcinaceae]**

CGAGCAGCCGCGGTAATACGGGGGGTGCAAGCGTTATTCGGAATTATTGGGCGTAAAGGGCGCGCAGGCGGCCTC
TTAAGTCAGATGTGAAAGCCCGGGGCTTAACCCCGGAAGTGCATTTGAAACTAAGGGGCTTGAGTATGGGAGAGG
GAAGTGGAATTCCTGGTGTAGCGGTGAAATGCGTAGATATCAGGAGGAACACCGGTGGCGAAGGCGGCTTCCTGG
ACCAATACTGACGCTGAGGCGCGAAGGCGTGGGGAGCAAACAGGATTAGATACCCTGGTAGTCCACGCAGTAAAC

GGTGAACACTAGGTGTAGCGGGTATTGACCCCTGCTGTGCCGCAGCTAACGCATTAAGTGTTCCGCCTGGGGAGTA
CGATCGCAAGATTAAAACTCAAAGAAATTGACGG

>denovo7466 P10_2 CT11 **[Desulfosarcinaceae]**

CCAGCAGCTGCGGTAATACGGGGGGTGCGAGCGTTATTCGGAATTATTGGGCGTAAAGGGCGCGTAGGCGGCTTC
TTAAGTCAGATGTGAAAGACCGGGGCTTAACCCCGGAAGTGCATTTGAAACTAAGAGGCTTGAGTTTGGGAGAGG
GAAGTGGAATTCCTGGTGTAGAGGGTAAATTCGTAGATATCAGGAGGAACACCGGTGGCGAAGGCGACTTCCTGG
ACCAATACTGACGCTGATGCGCGAAGGCGTGGGGAGCAAACAGGATTAGATACCCTGGTAGTCCACGCAGTAAAC
GGTGAACACTAGGTGTAGCGGGTATTGACCCCTGCGGTGCCGCAGTTAACGCATTAAGTGTTCCGCCTGGGGAGTA
CGACCGCAAGGTTAAAACTCAAAGAAATTGACGG

>denovo11126 P10_2 CT18 **[Desulfosarcinaceae]**

CCAGCAGCTGCGGTAATACGGGGGGTGCAAGCGTTATTCGGAATCACTGGGCGTAAAGAGCGCGTAGGCGGTCTC
TTAAGTCAGATGTGAAAGCCCGGGGCTCAACCCTGGAAGTGCATTTGAAACTAAGGGACTTGAGTATGGGAGAGG
GAAGTGGAATTCCTGGTGTAGCGGTGAAATGCGTAGATATCAGGAGGAACACCGGTGGCGATGGCGACTTCCTGG
ACCAATACTGACGCTGAGGCGCGAAGGCGTGGGGAGCAAACAGGATTAGATACCCTGGTAGTCCACGCAGTAAAC
GTTGAACACTAGGTGTAGCGGGTATTGACCCCTGCTGTGCCGCAGCTAACGCATTAAGTGTTCCGCCTGGGGAGTA
CGGCCGCAAGGCTAAAACTCAAAAGAATTGACGG

>denovo7630 P10_2 CT11 **[Desulfosarcinaceae]**

CCAGCAGCTGCGGTAACACGGAGGGTGCAAGCGTTATTCGGAATCACTGGGCGTAAAGAGCGCGTAGGCGGTTTC
TAAAGTCAGATGTGAAAGCCCGGGGCTTAACCCCGGAAGAGCATTTGAAACTTAGGGACTTGAGTATGAGAGAGG
GAAGTGGAATTCCTGGTGTAGCGGTGAAGTGCGTAGATATCAGGAGGAACACCGGTGGCGAAGGCGACTTCCTGG
ACCAATACTGACGCTGAGGCGCGAAGGCGTGGGGAGCAAACAGGATTAGATACCCTGGTAGTCCACGCAGTAAAC
GTTGAACACTAGGTGTAGCGGGTATTGACCCCTGCTGTGCCGCAGCTAACGCATTAAGTGTTCCGCCTGGGGAGTA
CGGCCGCAAGGCTAAAACTCAAAGGAATAGACGG

>denovo10919 P10_2 CT30 / P10_4 CT32 / P11_2 CT15 **[Desulfosarcinaceae]**

CCAGCAGCTGCGGTAACACGGAGGGTGCAAGCGTTATTCGGAATTATTGGGCGTAAAGAGCGCGTAGGTGGTCTCT
TAAGTCAGATGTGAAAGCCTTCCGCTTAACGGAAGAAGTGCATTTGAAACTAAGAGACTTGAGTATGGGAGAGGGA
AGTGGAATTCCTGGTGTAGAGGTGAAATTCGTAGATATCAGGAGGAACACCGGTGGCGAAGGCGACTTCCTGGAC
CAATACTGACGCTGAGGCGCGAAGGCGTGGGGAGCAAACAGGATTAGATACCCTGGTAGTCCACGCAGTAAACGG
TGAACACTAGGTGTAGCGGGTATTGACCCCTGCTGTGCCGCAGTTAACGCATTAAGTGTTCCGCCTGGGGAGTACG
ACCGCAAGGTTAAAACTCAAACGAATTGACGG

>denovo2205 P11_2 CT10 **[Desulfosarcinaceae]**

CCAGCAGCCGCGGTAATACGGGGGGTGCAAGCGTTATTCGGAATTATTGGGCGTAAAGAGCGCGTAGGCGGTTTT
GTAAGTCAGATGTGAAAGCCCGGGGCTTAACCCCGGAAGTGCATTTGAAACTACAGGACTTGAGTATGGGAGAGG
GAAGTGGAATTCCTGGTGTAGAGGTGAAATTCGTAGATATCAGGAGGAACACCGGTGGCGAAGGCGGCTTCCTGG
ACCAATACTGACGCTGAGGCGCGAAGGCGTGGGTAGCAAACAGGATTAGATACCCTGGTAGTCCACGCAGTAAAC
GGTGATCACTAGGTGTAGCGGGTATTGACCCCTGCTGTGCCGCAGTTAACGCATTAAGTGATCCGCCTGGGAAGTA
CGATCGCAAGATTAAAACTCAAAGAAATTGACGG

>denovo7484 P10_2 CT225 / P10_4 CT253 / P11_2 CT1353 **[uncultured Desulfobacteraceae]**

CCAGCAGCTGCGGTAATACGGAGGGTGCAAGCGTTATTCGGAATTACTGGGCGTAAAGGGCGCGTAGGCGGCTCC
TTAAGTCAGGTGTGAAAGCCCGGGGCTCAACCCCGGAAGTGCGTTTGAAACTAAGGAGCTTGAGTACGGGAGAGG
GAAGTGGAATTCCTGGTGTAGAGGTGAAATTCGTAGATATCAGGAGGAACACCGGTGGCGAAGGCGACTTCCTGG
ACCGATACTGACGCTAAGGCGCGAAAGCGTGGGGAGCAAACAGGATTAGAGACCCTGGTAGTCCACGCCGTAAAC
GATGAACACTAGGTGTAGCGGGTATTGACCCCCGCTGTGCCGTAGCTAACGCATTAAGTGTTCCGCCTGGGGACTA
CGGTCGCAAGGCTAAAACTCAAACGAATTGACGG

>denovo8688 P10_2 CT16 **[uncultured Desulfobacteraceae]**

CCAGCAGCTGCGGTAAGACGGAGGGTGCAAGCGTTATTCGGAATTACTGGGCGTAAAGGGCGCGTAGGCGGTTCA
TTAAGTCAGGCGTGAAAGACCGGGGCTCAACCCCGGAAGTGCGGTTGAAACTGAGGAGCTTGAGTACGGGAGAGG
GAAGGGGAATTCCTGGTGTAGAGGTGAAATTCGTAGATATCAGGAGGAACACCGGTGGCGAAGGCGACTTCCCGG
ACCGATACTGACGCTGAGGCGCGAAAGCGTGGGGAGCAAACAGGATTAGAGACCCTGGTAGTCCACGCCGTAAAC
GATGAACACTAGGTGTAGCGGGTATTGACCCCCGCTGTGCCGTAGCTAACGCATTAAGTGTTCCGCCTGGGGACTA
CGGCCGCAAGGCTAAAACTTAAAGGAATTGACGG

>denovo8573 P11_2 CT60 **[uncultured Desulfobacteraceae]**

CCAGCAGCTGCGGTAACACGGAGGGTGCAAGCGTTATTCGGAATTACTGGGCGTAAAGGGCGCGTAGGCGGCTCT
TTAAGTCAGGCGTGAAAGCCCAGGGCTCAACCCTGGAAGTGCGTTTGAAACTGAAGAGCTTGAGTACGGGAGAGG
GAAGTGGAATTCCTGGTGTAGAGGTGAAATTCGTAGATATCAGGAGGAACACCGGTGGCGAAGGCGACTTCCTGG
ACCGATACTGACGCTGAGGCGCGAAAGCGTGGGGAGCAAACAGGATTAGAGACCCTGGTAGTCCACGCCGTAAAC
GATGAACACTAGGTGTAGCGGGTATTGACCCCTGCTGTGCCGCAGCTAACGCACTAAGTGTTCCGCCTGGGGACTA
CGGTCGCAAGGCTAAAACTCAAAGAAATTGACGG

>denovo10909 P10_2 CT26 / P11_2 CT55 **[uncultured Desulfobacteraceae]**

CCAGCAGCTGCGGTAATACGGAGGGTGCAAGCGTTATTCGGAATTACTGGGCGTAAAGGGCGCGCAGGCGGTTCC
TTAAGTCAGGCGTGAAAGCCCGGGGCTAAACCTCGGAAGTGCGTCTGAAACTAAGGGACTTGAGTACGGGAGAGG
GAAGTGGAATTCCTGGTGTAGAGGTGAAATTCGTAGATATCAGGAGGAACACCGGTGGCGAAGGCGACTTCCTGG
ACCGATACTGACGCTGAGGCGCGAAAGCGTGGGGAGCAAACAGGATTAGAGACCCTGGTAGTCCACGCCGTAAAC
GATGAACACTAGGTGTAGCGGGTATTGAATCCTGCTGTGCCGCAGCTAACGCATTAAGTGTTCCGCCTGGGGACTA
CGGTCGCAAGGCTAAAACTCAAACGAATTGACGG

>denovo5431 P10_2 CT14 / P10_4 CT14 **[Desulfatiglans lineage]**

CCAGCAGCTGCGGTAACACGGAGGGTGCAAGCGTTGTTCGGAATTACTGGGCGTAAAGAGCGTGTAGGCGGTTTG
GCAAGTCAGATGTGAAAGCCCTGGGCTCAACCCCGGAAGTGCATTTGAAACTGCCATTTTAGAGTATGGGAGAGGA
GAGTGGAATTCCCAGTGTAGAGGTGAAATTCGTAGATATTGGGAGGAACACCCGTGGCGAAAGCGACTCTCTGGAC
CAATACTGACGCTGAGACGCGAAAGCGTGGGGAGCAAACAGGATTAGATACCCTGGTAGTCCACGCCGTAAACGA
TGAGAACTAGGTGTAGCGGGTATTGACCCCTGCTGTGCCGTAGCTAACGCATTAAGTACTCCGCCTGGGGAGTACG
GTCGTAAGGCTAAAACTTAAAGGAATTGACGG

>denovo16261 P10_2 CT11 **[Desulfatiglans lineage]**

CCAGCAGCTGCGGTAATACGGAGGGTGCAAGCGTTGTTCGGAATTACTGGGCGTAAAGAGCGTGTAGGCGGTTTG
GCAAGTCAGATGTGAAAGCCCTGGGCTCAACCCAGGAAGTGCATTTGAAACTGCCATACTAGAGTATGGGAGAGG
AGAGTGGAATTCCCGGTGTAGCGGTGAAATTCGTAGATAGTGGGAGGAACACCCGTGGCGAAGGCGACTCTCTGG
ACCAATACTGACGCTGGGACGCGAAAGCGTGGGGAGCAAACAGGATTAGATACCCTGGTAGTCCACGCCGTAAAC
GATGAGAACTAGGTGTAGCGGGTATTGACCCCTGCTGTGCCGTAGCTAACGCATTAAGTCCTCCGCCTGGGGAGTA
CGGTCGCAAGGCTAAAACTCAAAAGAATTGACGG

>denovo14186 P10_2 CT23 / P10_4 CT39 [**Desulfatiglans lineage]**

CCAGCAGCCGCGGTAACACGGAGGGTGCAAGCGTTGTTCGGAATTACTGGGCGTAAAGGGCGTGTAGGCGGTTTG
GCAAGTCAGATGTGAAAGCCCTGGGCTCAACCCAGGAAGTGCATTTGAAACTGCCATACTTGAGTATGGGAGAGGA
GAGTGGAATTCTCGGTGTAGAGGTGAAATTCGTAGATATTGAGAGGAACACCCGTGGCGAAGGCGACTCTCTGGAC
CAATACTGACGCTGAGACGCGAAAGCGTGGGGAGCAAACAGGATTAGATACCCTGGTAGTCCACGCTGTAAACGAT
GAGAACTAGGTGTAGCGGGTATTGACCCCTGCTGTGCCGCAGCTAACGCATTAAGTTCTCCGCCTGGGGAGTACGG
CCGCAAGGCTAAAACTTAAATGAATTGACGG

>denovo2861 P10_2 CT83 / P10_4 CT76 / P11_2 CT328 **[Desulfatiglans lineage]**

CCAGCAGCTGCGGTAATACGGAGGGTGCAAGCGTTGTTCGGAATTACTGGGCGTAAAGAGCGTGTAGGCGGTTTG
GTAAGTCAGATGTGAAAGCCCTGGGCTCAACCCAGGAAGTGCATTTGAAACTGCCTTTCTTGAGTATGGGAGAGGA
GAGTGGAATTCCCAGTGTAGAGGTGAAATTCGTAGATATTGGGAGGAACACCCGTGGCGAAGGCGACTCTCTGGA
CCAATACTGACGCTGAGACGCGAAAGCGTGGGGAGCAAACAGGATTAGATACCCTGGTAGTCCACGCCGTAAACG
ATGAGAACTAGGTGTAGCGGGTATTGACCCCTGCTGTGCCGAAGTTAACGCATTAAGTTCTCCGCCTGGGGAGTAC
GGCCGCAAGGCTAAAACTCAAACGAATTGACGG

>denovo11218 P11_2 CT15 **[Desulfatiglans lineage]**

CCAGCAGCTGCGGTAATACGGAGGGTGCGAGCGTTGTTCGGAATTATTGGGCGTAAAGGGCGTGCAGGCGTTATG
GTAAGTCAGATGTGAAAGCCTTCTGCTCAACGGAAGAAGTGCATTTGAAACTGCCATGCTTGAGTATGGGAGAGGA
GAGTGGAATTCCCAGTGTAGAGGTGAAATTCGTAGATATTGGGAGGAACACCCGTGGCGAAGGCGACTCTCTGGA
CCAATACTGACGCTGAGACGCGAAAGCGTGGGGAGCAAACAGGATTAGATACCCTGGTAGTCCACGCCGTAAACG
ATGAGAACTAGGTGTAGCGGGTATTGACCCCTGCTGTGCCGTAGCTAACGCACTAAGTTCTCCGCCTGGGGAGTAC
GGCCGCAAGGCTAAAACTTAAATGAATTGACGG

>denovo12378 P11_2 CT10 **[Desulfarculaceae-assigned]**

GCGGTAACACGGAGGGTGCAAGCGTTATTCGGATTTATTGGGCGTAAAGAGCGTGTAGGCGGTTGGGTTAGTCAG
ATGTGAAATCCTTCTGCTCAACAGAAGAAGTACGTCTGAAACTGCCCAGCTTGAGTACGAGAGAGGAAAGTGGAGT
TCCCAGTGTAGAGGTGAAATTCGTAGATATTGGGAGGAACACCTGTGGCGAAGGCGACTTTCTGGATCGATACTGA
CGCTGAGACGCGAAAGCGTGGGGAGCAAACAGGATTGGATACCCGGGTGGTCCACGCCGTAAACGATGGGAACTG
GGTGTAGCGGGTATTGATCCCTGCTGTGCCGAAGCTAACGCATTAAGTTCCCCGCCTGGGGAGTACGGTCGCAAGG
CTAAAACTTAAATGAATTGACGG

>denovo3371 P10_2 CT72 / P10_4 CT127 / P11_2 CT31 **[Desulfarculaceae-assigned]**

GCGGTAACACGGAGGGTGCAAGCGTTATTCGGATTTATTGGGCGTAAAGCGCGTGTAGGCGGTCGGGTTAGTCAG
ATGTGAAATCCTTCTGCTCAACAGAAGAAGTACGTCTGAAACTGCCCAACTAGAGTACGAGAGAGGAAAGTGGAAT
TCCCAGTGTAGAGGTGAAATTCGTAGATATTGGGAGGAACACCTGTGGCGAAGGCGACTTTCTGGATCGATACTGA
CGCTGAGACGCGAAAGCGTGGGGAGAAAACAGGATTAGATACCCTGGTAGTCCACGCCGTAAACGATGGGAACTA
GGTGTAGCGGGTATTGATCCCTGCGGTGCCGAAGCTAGCGCATTAAGTTCCCCGCCTGGGGAGTACGGTCGCAAGG
CTAAAACTTAAACGAATTGACGG

>denovo2296 P10_4 CT13 **[Desulfarculaceae-assigned]**

CCAGCAGCTGCGGTAGTACGGAGGGTGCAAGCGTTATTCGGATTTATTGGGCGTAAAGAGCGTGTAGGCGGTTGG
GTTAGTCAGATGTGAAATCCTTCTGCTCAACAGAAGAAGTACGTCTGAAACTGCCCAACTCGAGTACGAGAGAGGA
AAGTGGAATTCCTAGTGTAGAGGTGAAATTCGTAGATATTGGGAGGAACACCTGGGGCGAAGGCGACTTTCTGGAT
CGATACTGACGCTGAGACGCGAAAGCGTGGGGAGCAAACAGGATTAGATACCCTGGTAGTCCACGCCGTAAACGA

TGGGAACTAGGTGTAGCGGGTATTGATCCCTGCGGTGCCGAAGCTAACGCATTAAGTTCCCCGCCTGGGGAGTACG
GTCGCAAGGCTAAAATTCAAAGGAATAGACGG

>denovo2942 P10_4 CT10 **[Desulfarculaceae-assigned]**

CCAGCAGCTGCGGTAATACGGAGGGTGCAAGCGTTATTCGGATTTATTGGGCGTAAAGAGCGTGTAGGCGGTTGG
GTTAGTCAGATGTGAAATCCTTCTGCTCAACAGAAGAAATACGTCTGAAACTACCCAACTTGAGTACGAGAGAGGA
AAGTGGAATTCCCAGTGTAGAGGTAAAATTCGTAGATATTGGGAGGAACACCTGTGGCGAAGGCGACTTTCTGGAT
CGATACTGACGCTGGGACGCGAAAGCGTGGGTAGCAAACAGGATTAGATACCCTGGTAGTCCACGCCGTAAACGAT
GGGAACTAGGTTTAGCGGGGATTGATCCCTGCTGTGCCGGAGTTAACGCATTAAGTTCCCCGCCTGGGGAGTACGG
TCGCAAGGCTAAAAATCAAAGGAATAGACGG

>denovo7215 P10_4 CT10 **[Desulfarculaceae-assigned]**

CCAGCAGCCGCGGTAACACGGAGGGTGCAAGCGTTATTCGGATTTATTGGGCGTAAAGAGCGTGTAGGCGGTTGG
GTTAGTCAGATGTGAAATCCTTCTGCTCAACAGAAGAAGTACGTCTGAAACTACCCAACTTGAGTACGAGAGAGGG
AAGTGGAATTCCCAGTGTAGAGGTGAAATTCGGAGATATTGGGAGGAACACCTGTGGCGAAGGCGACTTTATGGA
TTGATACTGACGCTGAGACGCGAAAGCGTGGGTAGCAAACAGGATTAGATACCCTGGTAGTCCACGCCGTAAACGA
TGGGAACTAGGTGTAGCGGGGATTGATCCCTGCTGTGCCGGAGTTAACGCATTAAGTTCCCCGCCTGGGGAGTACG
GTCGCAAGGCTAAAACTCAAAGAAATTGACGG

>denovo5906 P10_2 CT20 / P10_4 CT108 **[Desulfarculaceae-assigned]**

CCAGCAGCTGCGGTAACACGGAGGGTGCAAGCGTTATTCGGATTTATTGGGCGTAAAGAGCGTGTAGGCGGTTTG
GTTAGTCAGATGTGAAATCCTTCTGCTCAAGTGAAGAAGTACGTCTGAAACTACCTAACTTGAGTACGAGAGAGGA
AAGTGGAATTCCCAATGTAGAGGTGAAATTCGTAGATATTGGGAGGAACACCTGTGGCGAAGGCGACTTTCTGGAT
CGATACTGACGCTGAGACGCGAAAGCGTGGGGAGCAAACAGGATTAGATACCCTGGTAGTCCACGCCGTAAACGA
TGGGAACTAGGTGTAGTGGGTATTGATCCCTGCGGTGCCGAAGCTAACGCATTAAGTTCCCCGCCTGGGGAGTACG
GTCGCAAGGCTAAAACTCAAAAGAATTGACGG

>denovo2340 P10_2 CT25 / P10_4 CT75 / P11_2 CT47 **[Desulfarculaceae-assigned]**

CCAGCAGCCGCGGTAATACGGAGGGTGCAAGCGTTATTCGGATTTATTGGGCGTAAAGAGCGTGTAGGTGGTCAG
GTCAGTCAGATGTGAAATCCTTCCGCTCAAGGGAAGAAGTACGTCTGAAACTACCTGACTTGAGTACGAGAGAGGA
AAGTGGAATTCCCAGTGTAGAGGTGAAATTCGTAGATATTGGGAGGAACACCTGTGGCGAAGGCGACTTTCTGGAT
CGATACTGACGCTGAGACGCGAAAGCGTGGGGAGCAAACAGGATTAGATACCCTGGTAGTCCACGCCGTAAACGA
TGGGAACTAGGTGCAGCGGGTATTGATCCCTGCTGTGCCGAAGCTAACGCATTAAGTTCCCCGCCTGGGGAGTACG
GTCGCAAGGCTAAAACTCAAAAGAATTGACGG

>denovo15603 P10_2 CT14|P10_4 CT14 **[Desulfarculaceae-assigned]**

TCAGCAGCCGCGGTAATACGGAGGGTGCGAGCGTTATTCGGATTTATTGGGCGTAAAGAGCGTGTAGGCGGTTGG
GTTAGTCAGATGTGAAATCCTTCTGCTCAAGTGAAGAAGTGCATCTGAAACTACCCAACTTGAGTACGAGAGAGGA
AAGTGGAATTCCCAGGGTAGAGGTGAAATTCGTAGATATTGGGAGGAACACCTGTGGAGAAGGCGACTTTCTGGA
TCGATACTGACGCTGAGACGCGAAAGCGTGGGGAGCAAACAGGATTAGATACCCTGGTAGTCCACGCCGTAAACG
ATGGGAACTAGGTGTAGTGGGTATTGAGCCCTGCTGTGCCGAAGCTAACGCATTAAGTTCCCCGCCTGGGGAGTAC
GGTCGCAAGGCTAAAACTTAAACGAATTGACGG

>denovo5856 P10_4 CT29 **[Desulfarculaceae-assigned]**

GCGGTAACACGGAGGGTGCGAGCGTTATTCGGATTTATTGGGCGTAAAGAGCGTGTAGGCGGTTGGGTTAGTCAG
ATGTGAAATCCTTCTGCTCAACGGAAGAAGTGCATCTGAAACTACCCGACTTGAGTACGAGAGAGGAAGGTGGAAT
TCCCAGTGTAGAGGTGAAATTCGTAGATATTGGGAGGAACACCTGTGGCGAAGGCGACTTTCTAGATCGATACTGA
CGCTGAGATGCGAAAGCGTGGGGAGAAAACAGGATTAGATACCCTGGTAGTCCACGCCGTAAACGATGGGAACTA
GGTGTAGCGGGTATTGATCCCTGCGGTGCCGGAGTTAACGCATTAAGTTCCCCGCCGGGGGAGTACGGTCGCAAG
GCTAAAACTTAAATGAATTGACGG

>denovo11131 P10_2 CT31 / P10_4 CT13 **[Desulfarculaceae-assigned]**

CCAGCGCCGCGGTAATACGGAGGGTGCGAGCGTTATTCGGATTTATTGGGCGTAAATAGCGTGTAGGCGGTTTGGT
TAGTCAGATGTGAAATCCTTCTGCTCAACGGAAGAAGGGCATCTGAAACTACCTGACTTGAGTACGAGAGAGGAAA
GTGGAATTCCCAGTGTAGAGGTGAAATTCGTAGATATTGGGAGGAACATCTGTGGCGAAGGCGACTTTCTAGATCG
ATACTGACGCTGAGACGCGAAAGCGTGGGTAGCAAACAGGATTAGATACCCTGGTAGTCCACGCCGTAAACGATG
GGAACTAGGTGTAGCGGGTATTGATCCCTGCTGTGCCGGAGTTAACGCATTAAGTTCCCCGCCTGGGGAGTACGGT
CGCAAGGCTAAAACTCAAATGAATTGACGG

>denovo13505 P104B CT31 **[Desulfarculaceae-assigned]**

CCAGCAGCTGCGGTAATACGGAGGGTGCGAGCGTTATTCAGATTTATTGGGCGTAAAGAGCGTGTAGGCGGTTTAG
TTAGTCAGATGTGAAATCCTTCTGCTCAACGGAAGAAGTGCATCTGAAACTGCCCAACTTGAGTACGAGAGGGGAA
AGTGGAATTCCCAGTGTAGAGGTGAAATTCGTAGATATTGGGAGGAACACCTGTAGCGAAGGCGACTTTCTGGATC
GATACTGACGCTGAGATGCAAAAGCGTGGGGAGCAAACAGGATTAGATACCCGGGTAGTCCACGCCGTAAACGAT
GGGAACTAGGTGTAGCGGGTATTGATCCCTGCTGTGCCGGAGTTAACGCATTAAGTTCCCCGCCTGGGGAGTACGG
TCGCAAGGCTAAAACTCAAAAGAATTGACGG

>denovo8635 P10_2 CT13 / P10_4 CT27 **[Desulfarculaceae-assigned]**

CCAGCAGCTGCGGTAATACGGAGGGTGCAAGCGTTATTCGGATTTATTGGGCGTAAAGAGCGTGTAGGCGGTTTG
GTTAGTCAGATGTGAAATCCTTCTGCTCAACAGAAGAAGTACGTCTGAAACTGCTCAACTTGAGTACGGGAGAGGA
AAGTGGAATTCCCAGTGTAGAGGTGAAATTCGTAGATATTGGGAGGAACACCTGTGGCGAAGGCGACTTTCTGGAT
CGATACTGACGCTGGGACGCGAAAGCGTGGGGAGCAAAGGGGATTAGATACCCGGGTAGTCCACGCCGTAAACGA
TGGGAACTAGGTGTAGCGGGTATTGATCCCTGCGGTGCCGAAGTTAACGCATTAAGTGCCCCGCCTGGGGAGTACG
GTCGCAAGGCTAAAACTCAAATGAATTGACGG

>denovo3340 P10_2 CT31 / P10_4 CT51 **[Desulfarculaceae-assigned]**

CCAGCAGCTGCGGTAATACGGAGGGTGCAAGCGTTATTCGGATTTATTGGGCGTAAAGAGCGTGTAGGCGGTTTG
GTTAGACAGATGTGAAATCCTTCTGCTCAACAGAAGAAGTACGTCTGAAACTGCTCAACTTGAGTACGAGAGAGGA
AAGTGGAATTCCCAGTGTAGAGGTGAGATTCGTAGATATTGCGAGGAACACCTGTGGCGAAGGCGACTTTCTGGAT
CGATACTGACGCTGAGACGCGAAAGCGGGGGGAGAAAACAGGATTAGATACCCTGGTAGTCCACGCCGTAAACGA
TGGGAACTAGGTGTAGCGGGTATTGATCCCTGCGGTGCCGAAGTTAACGCATTAAGTTCCCCGCCTGGGGAGTACG
GTCGCAAGGCTAAAACTCAAAGAAATTGACGG

>denovo8703 P10_4 CT15 **[Desulfarculaceae-assigned]**

CCAGCAGCCGCGGTAACACGGAGGGTGCAAGCGTTATTCGGATTTATTGGGCGTAAAGAGCGTGTAGGCGGTTGG
GTTAGTCAGATGTGAAATCCTTCTGCTCAACAGAAGAAGTACGTCTGAAACTGCTCAACTTGAGTACGGGAGAGGA
AAGTGGAATTCCCAGGGTAGAGGGGAAATTCGTAGATATTGGGAGGAACACCTGTGGCGAAGGGGACTTTCTGGA
TCGATACTGACGCTGGGACGCGAAAGGGTGGGGAGCAAACAGGATTAGATACCCTGGTAGTCCACGCCGTAAACG
ATGGGAACTAGGTGTAGCGGGTATTGATCCCTGCGGTGCCGAAGTTAACGCATTAAGTTCCCCGCCTGGGGAGTAC
GGTCGCAAGGCTAAAACTCAAAGAAATTGACGG

>denovo15352 P10_2 CT25 / P10_4 CT35 **[Desulfarculaceae-assigned]**

CCAGCAGCCGCGGTAACACGGAGGGTGCAAGCGTTATTCGGGTTTATTGGGCGTAAAGAGCGTGTAGGCGGTTTG
GTTAGTCAGATGTGAAATCCTTCTGCTCAACAGAAGAAGTACGTCTGAAACTGCTCAACTTGAGTACGAGAGAGGA
AAGTGGAATTCCCAATGTAGAGGTGAAATTCGTAGATATTGGGAGGAACACCTGTGGCGAAGGCGACTTTCTGGAT
CGATGCTGACGCTGAGCCGCGAAAGCGTGGGGAGCAAACAGGATTATATACCCTGGTAGCCCACGCCGTAAACGAT
GGGAACTAGGTGTAGCGGGTATTGATCCCTGCTGTGCCGAAGTTAACGCATTAAGTTCCCCGCCTGGGGAGTACGG
TCGCAAGGCTAAAACTCAAAGGAATTGACGG

>denovo2122 P10_2 CT28 / P10_4 CT31 **[Desulfarculaceae-assigned]**

CCAGCAGCTGCGGTAACACGGAGGGTGCAAGCGTTATTCGGATTTATTGGGCGTAAAGAGCGTGTAGGCGGTTGG
GTTAGTCAGATGTGAAATCCTTCTGCTCAACAGAAGAAGTACGTCTGAAGCTGCTCAACTTGAGTACGAGAGAGGA
AAGTGGAATTCCCAATGTAGAGGTGAAATTCGTAGATATTGGGAGGAACACCTGTGGCGAAGGCGACTTTCTGGAT
CGATACTGACGCTGGGACGCGAAAGCGTGGGGAGCAAACAGGTTTAAAATCCCTGGTAGTCCACGCCGTAAACGAT
GGGAACTAGGTGTAGCGGGTATTGATCCCTGCTGTGCCGAAGTTAACGCATTAAGTTCCCCGCCTGGGGAGTACGG
TCGCAAGGCTAAAACTCAAACGAATTGACGG

>denovo5131 P10_2 CT22 / P10_4 CT27 **[Desulfarculaceae-assigned]**

CCAGCAGCTGCGGTAAGACGGAGGGTGCAAGCGTTATTCGGATTTATTGGGCGTAAAGAGCGTATAGGCGGTTTG
GTTAGTCAGATGTGAAATCCTTCTGCTCAACAAAAGAAGTACGTCTGAAACTGCTCAACTTGAGTACGAGAGAGGA
AAGTGGAATTCCCAATGTAGAGGTGAAACTCGTAGATATTGGGAGGAACACCTGTGGCGAAGGCGACTTTCTGGAT
CGATACTGACGCTGAGACGCGAAAGCGTGGGGAGCAAACAGGATTAGATACCCTGTTAGTCCACGCCGTAAACGAT
GGGAACTAGGTGTAGCGGGTATTGATCCCTGCTGTGCCGAAGTTAACGCATTAAGTTCCCCGCCTGGGGAGTACGG
TCGCATGGCTAAAACTCAAAAGAATTGACGG

>denovo9277 P10_4 CT18 **[Desulfoarculaceae-assigned]**

CCAGCAGCTGCGGTAACACGGAGGGTGCAAGCGTTATTCGGATTTATTGGGCGTAAAGAGCGTGTAGGCGGTTTG
GTTAGTCAGATGTGAAATCCTTCTGCTCAACCGCAGAAGTACGTCTGAAACTGCTCAACTTGAGTACGAGAGAGGAA
AGTGGAATTCCCAGTGTAGCGGTGAAATTCGTAGATATTGGGAGGAACACCTGTGGCGAAGGCGACTTACTGGATC
GATACTGACGCTGAGACGCGAAAGCGTGGGGAGCAAACAGGATTAGATACCCTGGTAGTCCACGCCGTAAACGAT
GGGAACTAGGTGTAGCGGGTATTGACCCCTGCTGTGCCGAAGTTAACGCATTAAGTGCCCCGCCTGGGGAGTACGG
TCGCAAGGCTAAAACTCAAACGAATTGACGG

>denovo14994 P10_2 CT905 / P10_4 CT1457 / P11_2 CT296 **[Desulfarculaceae-assigned]**

CCAGCAGCCGCGGTAATACGGAGGGTGCAAGCGTTATTCGGATTTATTGGGCGTAAAGAGCGTGTAGGCGGTTGG
GTTAGTCAGATGTGAAATCCTTCTGCTCAACGGAAGAAGTGCGTCTGAAACTGCTCAACTTGAGTACGAGAGAGGA
AAGTGGAATTCCCAGTGTAGAGGTGAAATTCGTAGATATTGGGAGGAACACCTGTGGCGAAGGCGACTTTCTGGAT
CGATACTGACGCTGAGACGCGAAAGCGTGGGGAGCAAACAGGATTAGATACCCTGGTAGTCCACGCCGTAAACGA
TGGGAACTAGGTGTAGCGGGTATTGATCCCTGCTGTGCCGGAGTTAACGCATTAAGTTCCCCGCCTGGGGAGTACG
GTCGCAAGGCTAAAACTTAAAAGAATTGACGG

>denovo14641 P10_2 CT87 / P10_4 CT291 / P11_2 CT2262 **[Desulfarculaceae-assigned]**

CCAGCAGCTGCGGTAATACGGAGGGTGCAAGCGTTATTCGGATTTATTGGGCGTAAAGAGCGTGTAGGTGGTTGG
GATAGTCAGATGTGAAAGCCTTCTGCTCAACAGAAGAAGTACGTCTGAAACTACCCAACTTGAGTACGAGAGAGGA
AAGTGGAATTCCCAGTGTAGAGGTGAAATTCGTAGATATTGGGAGGAACACCTGTGGCGAAGGCGACTTTCTGGAT
CGATACTGACGCTGAGACGCGAAAGCGTGGGGAGCAAACAGGATTAGATACCCTGGTAGTCCACGCTGTAAACGA

TGGGAACTAGGTGTAGCGGGTATTGATCCCTGCTGTGCCGAAGCTAACGCATTAAGTTCCCCGCCTGGGGAGTACG
GTCGCAAGGCTAAAACTCAAACGAATTGACGG

>denovo9700 P10_4 CT12 **[Desulfarculaceae-assigned]**

CCAGCAGCTGCGGTAATACGGAGGGTGCAAGCGTTATTCGGATTCATTGGGCGTAAAGCGCGTGTAGGCGGTTGG
GGTAGTCAGGTGTGAAAGCCTTCTGCTCAACAGAAGAAGTACATCTGGAACTGCCCGACTTGAGTACGAGAGAGGA
AAGTGGAATTCCCAGTGTAGAGGTGAAATTCGTAGATATTGGGAGGAAGACCTGTGGCGAAGGCGACTTTCTGGAT
CGTTACTGACGCTGAGCCGCGAAAGCGTGGGGAGCAAACAGGATTAGATACCCTGGTAGTCCACGCCGTAAACGAT
GGGAACTAGGTGTAGCGGGTATTGATCCCTGCTGTGCCGAAGCTAACGCATTAAGTTCCCCGCCTGGGGAGTACGG
TCGCAAGGCTAAAACTCAAAAGAATTGACGG

>denovo16628 P10_2 CT23 / P10_4 CT281 / P11_2 CT136 **[Desulfarculaceae-assigned]**

CCAGCAGCTGCGGTAACACGGAGGGTGCAAGCGTTATTCGGATTTATTGGGCGTAAAGAGCGTGTAGGCGGTTAG
GTCAGTCAGATGTGAAAGCCTTCTGCTCAACGGAAGAAGTGCATCTGAAACTACCTAACTTGAGTACGGGAGAGGA
AAGTGGAATTCCCAGTGTAGAGGTGAAATTCGTAGATATTGGGAGGAACACCTGTGGCGAAGGCGACTTTCTGGAT
CGATACTGACGCTGAGACGCGAAAGCGTGGGGAGCAAACAGGATTAGATACCCTGGTAGTCCACGCCGTAAACGA
TGGGAACTAGGTGTAGCGGGTATTGATCCCTGCTGTGCCGAAGCTAACGCATTAAGTTCCCCGCCTGGGGAGTACG
GTCGCAAGGCTAAAACTTAAAGGAATTGACGG

>denovo5030 P11_2 CT12 **[uncultured group]**

CCAGCAGCCGCGGTAACACGGAGGGTGCAAGCGTTGTTCGGAATCACTGGGCGTAAAGGGCGAGCAGGCGGTTG
GGTAAGTCAGGTGTGAAATCCCTAGGCTCAACCTAGGAAGTGCATTTGATACTGCCCATCTTGAGTACGGGAGAGG
GAGGCGGAATTCCCAGTGTAGAGGTGAAATTCGTAGATACTGGGAGGAACACCGGTGGCGAAGGCGGCCTCCTGG
ACCGATACTGACGCTGAGGCGCGAAAGCGTGGGGAGCAAACAGGATTAGATACCCTGGTAGTCCACGCTGTAAAC
GGTGAGCACTAGGTGTAGCGGGTATTGACCCCTGCTGTGCCGTAGCTAACGCATTAAGTGCTCCGCCTGGGGAGTA
CGACCGCAAGGTTGAAACTCAAATGAATTGACGG
